# Pharmacological blood pressure lowering for primary and secondary prevention of cardiovascular disease across different levels of blood pressure: an individual participant-level data meta-analysis

**DOI:** 10.1016/S0140-6736(21)00590-0

**Published:** 2021-05-01

**Authors:** The Blood Pressure Lowering Treatment Trialists' Collaboration, Amanda Adler, Larry Agodoa, Ale Algra, Folkert W Asselbergs, Nigel S Beckett, Eivind Berge, Henry Black, Frank P J Brouwers, Morris Brown, Christopher J Bulpitt, Robert P Byington, John Chalmers, William C Cushman, Jeffrey Cutler, Barry R Davis, Richard B Devereaux, Jamie Dwyer, Ray Estacio, Robert Fagard, Kim Fox, Tsuguya Fukui, Ajay K Gupta, Rury R Holman, Yutaka Imai, Masao Ishii, Stevo Julius, Yoshihiko Kanno, Sverre E Kjeldsen, John Kostis, Kizuku Kuramoto, Jan Lanke, Edmund Lewis, Julia B Lewis, Michel Lievre, Lars H Lindholm, Stephan Lueders, Stephen MacMahon, Giuseppe Mancia, Masunori Matsuzaki, Maria H Mehlum, Steven Nissen, Hiroshi Ogawa, Toshio Ogihara, Takayoshi Ohkubo, Christopher R Palmer, Anushka Patel, Carl J Pepine, Marc Allan Pfeffer, Bertram Pitt, Neil R Poulter, Hiromi Rakugi, Giuseppe Reboldi, Christopher Reid, Giuseppe Remuzzi, Piero Ruggenenti, Takao Saruta, Joachim Schrader, Robert Schrier, Peter Sever, Peter Sleight, Jan A Staessen, Hiromichi Suzuki, Lutgarde Thijs, Kenji Ueshima, Seiji Umemoto, Wiek H van Gilst, Paolo Verdecchia, Kristian Wachtell, Paul Whelton, Lindon Wing, Mark Woodward, Yoshiki Yui, Salim Yusuf, Alberto Zanchetti, Zhen-Yu Zhang, Craig Anderson, Colin Baigent, Barry Morton Brenner, Rory Collins, Dick de Zeeuw, Jacobus Lubsen, Ettore Malacco, Bruce Neal, Vlado Perkovic, Anthony Rodgers, Peter Rothwell, Gholamreza Salimi-Khorshidi, Johan Sundström, Fiona Turnbull, Giancarlo Viberti, Jiguang Wang

## Abstract

**Background:**

The effects of pharmacological blood pressure lowering at normal or high-normal blood pressure ranges in people with or without pre-existing cardiovascular disease remains uncertain. We analysed individual participant data from randomised trials to investigate the effects of blood pressure lowering treatment on the risk of major cardiovascular events by baseline levels of systolic blood pressure.

**Methods:**

We did a meta-analysis of individual participant-level data from 48 randomised trials of pharmacological blood pressure lowering medications versus placebo or other classes of blood pressure-lowering medications, or between more versus less intensive treatment regimens, which had at least 1000 persons-years of follow-up in each group. Trials exclusively done with participants with heart failure or short-term interventions in participants with acute myocardial infarction or other acute settings were excluded. Data from 51 studies published between 1972 and 2013 were obtained by the Blood Pressure Lowering Treatment Trialists' Collaboration (Oxford University, Oxford, UK). We pooled the data to investigate the stratified effects of blood pressure-lowering treatment in participants with and without prevalent cardiovascular disease (ie, any reports of stroke, myocardial infarction, or ischaemic heart disease before randomisation), overall and across seven systolic blood pressure categories (ranging from <120 to ≥170 mm Hg). The primary outcome was a major cardiovascular event (defined as a composite of fatal and non-fatal stroke, fatal or non-fatal myocardial infarction or ischaemic heart disease, or heart failure causing death or requiring admission to hospital), analysed as per intention to treat.

**Findings:**

Data for 344 716 participants from 48 randomised clinical trials were available for this analysis. Pre-randomisation mean systolic/diastolic blood pressures were 146/84 mm Hg in participants with previous cardiovascular disease (n=157 728) and 157/89 mm Hg in participants without previous cardiovascular disease (n=186 988). There was substantial spread in participants' blood pressure at baseline, with 31 239 (19·8%) of participants with previous cardiovascular disease and 14 928 (8·0%) of individuals without previous cardiovascular disease having a systolic blood pressure of less than 130 mm Hg. The relative effects of blood pressure-lowering treatment were proportional to the intensity of systolic blood pressure reduction. After a median 4·15 years' follow-up (Q1–Q3 2·97–4·96), 42 324 participants (12·3%) had at least one major cardiovascular event. In participants without previous cardiovascular disease at baseline, the incidence rate for developing a major cardiovascular event per 1000 person-years was 31·9 (95% CI 31·3–32·5) in the comparator group and 25·9 (25·4–26·4) in the intervention group. In participants with previous cardiovascular disease at baseline, the corresponding rates were 39·7 (95% CI 39·0–40·5) and 36·0 (95% CI 35·3–36·7), in the comparator and intervention groups, respectively. Hazard ratios (HR) associated with a reduction of systolic blood pressure by 5 mm Hg for a major cardiovascular event were 0·91, 95% CI 0·89–0·94 for partipants without previous cardiovascular disease and 0·89, 0·86–0·92, for those with previous cardiovascular disease. In stratified analyses, there was no reliable evidence of heterogeneity of treatment effects on major cardiovascular events by baseline cardiovascular disease status or systolic blood pressure categories.

**Interpretation:**

In this large-scale analysis of randomised trials, a 5 mm Hg reduction of systolic blood pressure reduced the risk of major cardiovascular events by about 10%, irrespective of previous diagnoses of cardiovascular disease, and even at normal or high–normal blood pressure values. These findings suggest that a fixed degree of pharmacological blood pressure lowering is similarly effective for primary and secondary prevention of major cardiovascular disease, even at blood pressure levels currently not considered for treatment. Physicians communicating the indication for blood pressure lowering treatment to their patients should emphasise its importance on reducing cardiovascular risk rather than focusing on blood pressure reduction itself.

**Funding:**

British Heart Foundation, UK National Institute for Health Research, and Oxford Martin School.

**Research in context**

**Evidence before this study**

We searched PubMed, MEDLINE, the Cochrane central register of controlled trials, and ClinicalTrials.gov for randomised controlled trials investigating blood pressure-lowering drug treatment, covering the period between Jan 1, 1966, and Sept 1, 2019, with no language restrictions. We searched MEDLINE using and expanding on the MeSH terms for “hypertension”, “blood pressure”, and “antihypertensive agents” including possible variations of the terms and relevant antihypertensive drug classes. We identified several individual trials and tabular meta-analyses with conflicting or little information on the effect of blood pressure-lowering treatment stratified by baseline blood pressure and previous cardiovascular disease status. In particular, information relating to heterogeneous treatment effects at normal or high-normal blood pressure categories was scant.

**Added value of this study**

In this collaborative project, we gathered individual participant-level data (IPD) from eligible large-scale trials of blood pressure-lowering treatment. With access to IPD from about 350 000 patients randomly assigned to treatment in 48 trials, this analysis is the largest and most detailed investigation of the stratified effects of pharmacological blood pressure-lowering. Participants were first divided into two groups: those with a previous diagnosis of cardiovascular disease and those without. Each group was then divided further into seven subgroups based on systolic blood pressure at study entry (<120, 120–129, 130–139, 140–149, 150–159, 160–169, and ≥170 mm Hg). Over an average 4 years of follow-up, a 5 mm Hg reduction in systolic blood pressure lowered the relative risk of major cardiovascular events by 10%. The risks for stroke, heart failure, ischaemic heart disease, and death from cardiovascular disease were reduced by 13%, 13%, 8%, and 5%, respectively. Relative risk reductions were proportional to the intensity of blood pressure-lowering. Neither the presence of cardiovascular disease or the level of blood pressure at study entry modified the effect of treatment.

**Implications of all the available evidence**

This study calls for a change in clinical practice that predominantly confines antihypertensive treatment to people with higher than average blood pressure values. On the basis of this study, the decision to prescribe blood pressure medication should not be based simply on a previous diagnosis of cardiovascular disease or an individual's current blood pressure. Rather, blood pressure medication should be viewed as an effective tool for preventing cardiovascular disease when an individual's cardiovascular risk is elevated. Recommendations that specify a minimum blood pressure threshold for initiation or intensification of treatment, or a floor level for blood pressure reduction are not substantiated by this study.

## Introduction

Randomised trials of treatments to lower blood pressure have established that pharmacological reduction of blood pressure is an effective strategy to reduce the risk of cardiovascular events in a range of at-risk populations.^1^ However, clinically important questions and uncertainties remain, as evident by conflicting guideline recommendations.^2^, ^3^, ^4^, ^5^ Two major, but related, controversies involve the potential differential effects of blood pressure-lowering treatment between individuals with and without a previous history of cardiovascular disease, and when blood pressure is within the so-called normal range or only mildly elevated. An analysis^6^ of a contemporary registry of participants with ischaemic heart disease reported a J-shaped association between cardiovascular events and blood pressure, with the lowest risk of events occurring at blood pressure levels around 130/75 mm Hg, suggesting that reducing blood pressure below this level could increase the risk of cardiovascular events. Another study^7^ that included participants with a history of cardiometabolic conditions concluded that the lowest blood pressure possible is not necessarily the optimal target for high-risk participants. These findings have not been confined to observational studies or people with pre-existing cardiovascular disease; a randomised trial came to a similar conclusion for treating individuals without cardiovascular disease who had a systolic blood pressure of less than 140 mm Hg.^8^

Evidence for blood pressure-lowering treatment interactions with previous cardiovascular disease and baseline blood pressure values from tabular meta-analyses of randomised trials has been conflicting. A large-scale tabular meta-analysis^1^ found no evidence for any interaction; by contrast, another study reported that antihypertensive treatment does not reduce the risk of cardiovascular disease in people without previous cardiovascular disease when systolic blood pressure is less than 140 mm Hg.^9^ These conflicting reports arise partly from the fact that previous meta-analyses did not have sufficient data at the level of the individual to be able to precisely classify participants based on their baseline disease status and blood pressure level. Additionally, their use of aggregate trial-level data on blood pressure values and disease status could have led to potentially misleading conclusions. Indeed, tabular meta-analyses by design are limited in cross-stratification of people, which is at the core of this controversy.

The current cycle of the Blood Pressure Lowering Treatment Trialists' Collaboration (BPLTTC; a group of the principal investigators of major randomised trials of pharmacological blood pressure-lowering treatment) provides the largest collection of individual participant-level information on blood pressure-lowering trials currently available.^10^, ^11^ It offers an opportunity to investigate the question of blood pressure-lowering effects across different baseline blood pressure thresholds and previous cardiovascular disease status in much greater depth and detail than previously possible. Therefore, we did an individual participant-level data (IPD) meta-analysis of randomised clinical trials to investigate the effects of pharmacological blood pressure-lowering treatment on the risk of major cardiovascular events in participants with and without previous cardiovascular disease at baseline, and to examine these effects by baseline levels of systolic blood pressure.

## Methods

### Study governance and data source

This meta-analysis was based on resources provided by the BPLTTC. The BPLTTC steering committee provides general oversight and scientific leadership on proposal development, analysis, interpretation, and reporting. An international network of collaborators contributes further to all scientific activities and discussions. The collaboration is coordinated at the University of Oxford (Oxford, UK), and has currently access to IPD from 51 randomised trials.^11^ The primary criteria for inclusion in the BPLTTC are trials that randomly assigned participants to pharmacological blood pressure-lowering medications versus placebo or other classes of blood pressure-lowering medications, or between more versus less intensive treatment regimens, which had at least 1000 persons-years of follow-up in each randomly allocated group.^11^ Trials that only included participants with heart failure or short-term interventions in participants with acute myocardial infarction or other acute settings were excluded. Ethics approval for the current phase was obtained from the Oxford Tropical Research Ethics Committee (OxTREC Reference 545–14) and our study protocol was approved by the BPLTTC steering committee and collaborators before releasing the data for analysis.

### Study selection criteria

For this study, we included BPLTTC trials that had collected and shared information about (1) baseline blood pressure measurements, (2) primary and secondary outcomes, with the timing of events, and (3) the presence or absence of previous cardiovascular disease at baseline. Cardiovascular disease status at baseline was defined as any reports of stroke, myocardial infarction, or ischaemic heart disease before randomisation. Participants with a known diagnosis of heart failure at baseline were excluded in our analysis from trials that had included such participants, consistent with our overall protocol of excluding trials exclusively done with participants with heart failure.

### Definition of outcomes and comparison groups

The primary outcome was a major cardiovascular event, defined as a composite of fatal or non-fatal stroke, fatal or non-fatal myocardial infarction or ischaemic heart disease, or heart failure causing death or requiring admission to hospital. The secondary outcomes were all-cause death, death due to cardiovascular disease, and each component of the primary outcome (as defined previously). Individual trials were categorised into two groups of intervention and comparator. For placebo-controlled trials, the placebo group was considered as the comparator and the active drug group as the intervention. For trials with two or more active groups, including trials comparing different drug classes, the group in which the blood pressure reduction was greater was considered as the intervention and the other treatment group(s) as the comparator. Trials that compared more intense versus less intense treatment were classified as the intervention and comparator groups, respectively. Detailed information about the comparison groups, trial design, participant characteristics, and level of blood pressure reduction for each trial have been reported previously.^10^

### Statistical analysis

We analysed the data as per intention to treat on the basis of the groups to which each participant had initially been assigned (intervention *vs* comparator). Data for each individual participant of each trial were harmonised before statistical analysis and merged into a single dataset to facilitate a one-stage IPD meta-analysis. We used stratified Cox proportional hazard models, with fixed treatment effects, and participants as the unit of analysis.^12^ Stratified models enabled us to specify the baseline hazard function for each trial to satisfy the proportional hazards assumption.^12^ Participants entered the analysis at the date of the randomisation and were followed up until the earliest occurrence of the outcome of interest, or death, or the end of the trial. The average systolic blood pressure reduction between randomised groups, excluding the first 12 months, amongt all included trials was 6·3 mm Hg (95% CI 6·1–6·4).^10^ To assess the risk reduction across blood pressure categories, we stratified the population into seven categories of baseline systolic blood pressure (<120, 120–129, 130–139, 140–149, 150–159, 160–169, and ≥170 mm Hg). We standardised the effect sizes for a 5 mm Hg reduction in systolic blood pressure between randomised groups as a convenient round number close to the average systolic blood pressure reduction (appendix p 4). Hazard ratios (HR) and their 95% CI were thus presented using forest plots with standardisation by 5 mm Hg reduction in systolic blood pressure. To test whether treatment effects varied across pre-specified subgroups by cardiovascular disease status and blood pressure levels at baseline, we used likelihood-ratio tests for interactions using Hommel's method for multiple testing adjustment.^13^, ^14^ Event rates were calculated using Kaplan-Meier estimates of cumulative incidence and separately plotted for participants with and without previous cardiovascular disease at baseline.

We did a meta-regression analysis to explore the effect of a unit decrease in blood pressure on change in the risk of major cardiovascular disease at trial-level. For these analyses, we used values for blood pressure reduction for each trial, with the overall achieved blood pressure reduction between comparison groups being calculated using linear mixed models, excluding the first-year blood pressure measurements.^10^ The corresponding HRs for each trial were estimated using Cox regression.

In response to reviewer comments, we did several complementary and sensitivity analyses. In the complementary analyses, we additionally reported the unstandardised effect and calculated the absolute risk reductions using a Poisson regression model with identity link for each stratum to investigate the heterogeneity of treatment effects on the absolute scale. In the sensitivity analyses, first we excluded the drug-class comparison trials and re-estimated the effect sizes. Second, to explore the possibility of an outlier trial having undue influence, a leave-one-out sensitivity analysis was done by removing each individual trial from the analysis in turn. Finally, we compared our chosen one-stage meta-analysis with a two-stage meta-analysis for the primary outcome for individuals with and without previous cardiovascular disease at baseline. For the two-stage meta-analysis, Cox regression models were used to obtain estimates from each trial, which were combined using fixed-effect models and standardised by a 5 mm Hg reduction in systolic blood pressure using the method previously reported.^1^

We reported the risk of bias using the revised Cochrane risk-of-bias tool,^15^ and did a sensitivity analysis excluding four trials at risk of bias. We investigated for potential data acquisition bias using a funnel plot as a visual inspection of the bias and Egger's regression test using trials included in this study. Analyses were done using R, version 3.3.

### Role of the funding source

The funders of the study had no role in study design, data collection, data analysis, data interpretation, writing of the report, or the decision to submit.

## Results

Of 51 randomised trials identified by the BPLTTC, all but three trials, involving 4967 participants, shared the necessary information for inclusion in our study (two did not provide information about cardiovascular disease status at baseline,^16^, ^17^ and one did not report the outcome of interest).^18^ We excluded data for 4138 individuals with a known diagnosis of heart failure at baseline (who had been in trials that had included such participants). Therefore, data for 344 716 randomly assigned participants from 48 trials were included in this meta-analysis (appendix pp 5–8). Of these 48 trials, 37 included both participants with and without previous cardiovascular disease at baseline, ten were restricted to participants with previous cardiovascular disease only at baseline, and one trial was done exclusively in participants without previous cardiovascular disease at baseline (appendix pp 5–8). There were no reports of heart failure outcome in five trials and no cardiovascular deaths in four trials (appendix pp 5–8). Mean age at baseline was 65 years, both in participants with and without previous cardiovascular disease (table). The proportion of women without previous cardiovascular disease was higher than the proportion of women with previous cardiovascular disease at baseline. The prevalence of atrial fibrillation and diabetes were similar in participants with and without previous cardiovascular disease, but peripheral vascular disease was numerically more common in participants with previous cardiovascular disease, and chronic kidney disease was more common in participants without previous cardiovascular disease. Mean systolic/diastolic blood pressure was lower in those with prevalent cardiovascular disease than those without, reflecting the selection criteria in those trials. However, the spread in blood pressure was similar in those with and without prevalent cardiovascular disease (SDs of 20 and 20, respectively). 31 239 (19·8%) of 157 728 participants with previous cardiovascular disease and 14 928 (8·0%) of 186 988 without previous cardiovascular disease had a systolic blood pressure of less than 130 mm Hg at baseline. Detailed characteristics of participants stratified by previous cardiovascular disease status and systolic blood pressure at baseline are in the table and the appendix (p 10).

**Figure 1:**
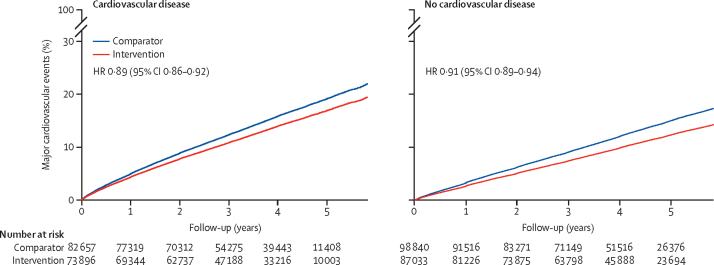
Rates of major cardiovascular events per 5 mm Hg reduction in systolic blood pressure, stratified by treatment allocation and cardiovascular disease status at baseline Major cardiovascular events were defined as a composition of fatal or non-fatal stroke, fatal or non-fatal myocardial infarction or ischaemic heart disease, or heart failure causing death or requiring admission to hospital. HR=hazard ratio.

After a median 4·15 years' follow-up (Q1–Q3 2·97–4·96), 42 324 participants (12·3%) had at least one major cardiovascular event, including 13 772 participants (4·0%) with stroke, 19 452 (5·6%) with ischaemic heart disease and 7833 (2·4%) with heart failure. Of the 28 895 participants (8·4%) who died, 10 935 (3·4%) were due to cardiovascular disease. Among participants without previous cardiovascular disease at baseline, the incidence rate for developing a major cardiovascular events per 1000 person-years was 31·9 (95% CI 31·3–32·5) in the comparator group and 25·9 (25·4–26·4) in the intervention group. In participants with previous cardiovascular disease at baseline, the corresponding rates were 39·7 (95% CI 39·0–40·5) in the comparator group and 36·0 (35·3–36·7) in the intervention group, respectively (figure 1). The HRs associated with a reduction of systolic blood pressure by 5 mm Hg for major cardiovascular events at the end of follow-up were 0·89 (95% CI 0·86–0·92) in those with previous cardiovascular disease and 0·91 (0·89–0·94) among participants without previous cardiovascular disease (figure 1). We did not find an observable difference in the patterns of events occurring over time for secondary outcomes compared with our primary outcome between participants with and without previous cardiovascular disease (appendix pp 14–18).

**Figure 2:**
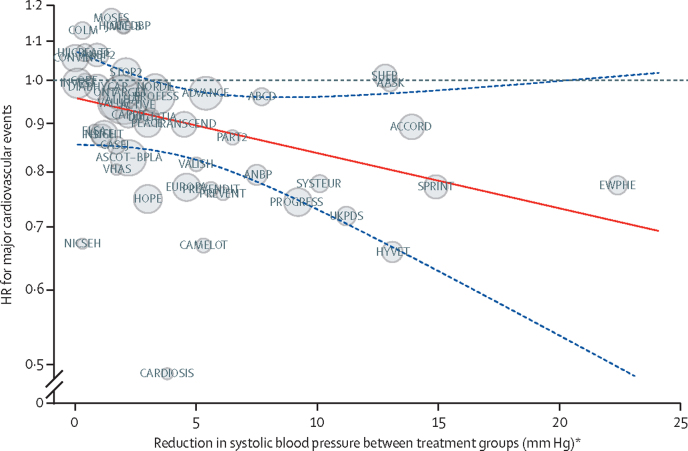
Association between the intensity of blood pressure reduction and relative treatment effects for prevention of major cardiovascular events The centre of the bubbles indicates the HR for each trial, with the size of the bubble inversely proportional to the respective SE. The solid red line is the fitted regression line; the dashed blue lines indicate 95% CI; and the dashed grey line indicates HR=1·0. HR=hazard ratio. *Excluding the first 12 months after randomisation.

**Figure 3:**
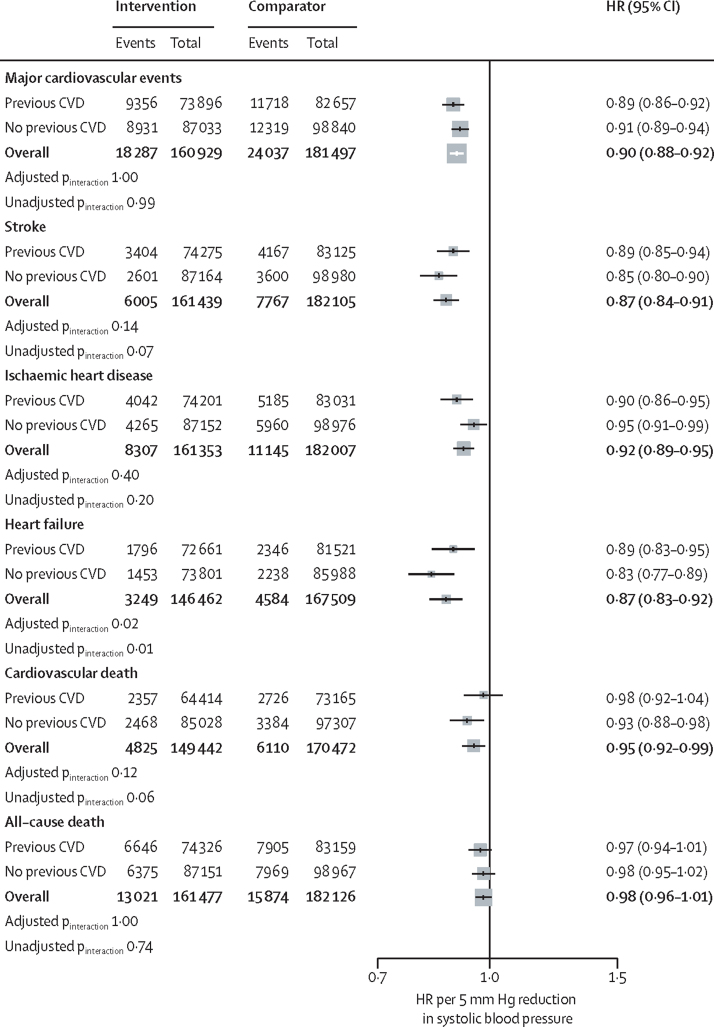
Effects of blood pressure-lowering treatment on primary and secondary outcomes, by cardiovascular disease status at baseline Forest plot shows the HRs and 95% CIs per 5 mm Hg reduction in systolic blood pressure, separately for each outcome. Adjusted p_interaction_ values were adjusted for multiple testing using Hommel's method. Unadjusted p_interaction_ values were not adjusted for multiple testing. HR=hazard ratio. CVD=cardiovascular disease.

Meta-regression analyses showed the HRs for major cardiovascular events to be proportional to the magnitude of the systolic blood pressure reduction achieved at the level of a trial (figure 2). Overall, a 5 mm Hg systolic blood pressure reduction reduced the risk of major cardiovascular events, regardless of stratification by cardiovascular disease (figure 3). There was also no evidence of any clinically meaningful heterogeneous treatment effect between groups with and without previous cardiovascular disease at baseline for the secondary outcomes (figure 3).

**Figure 4:**
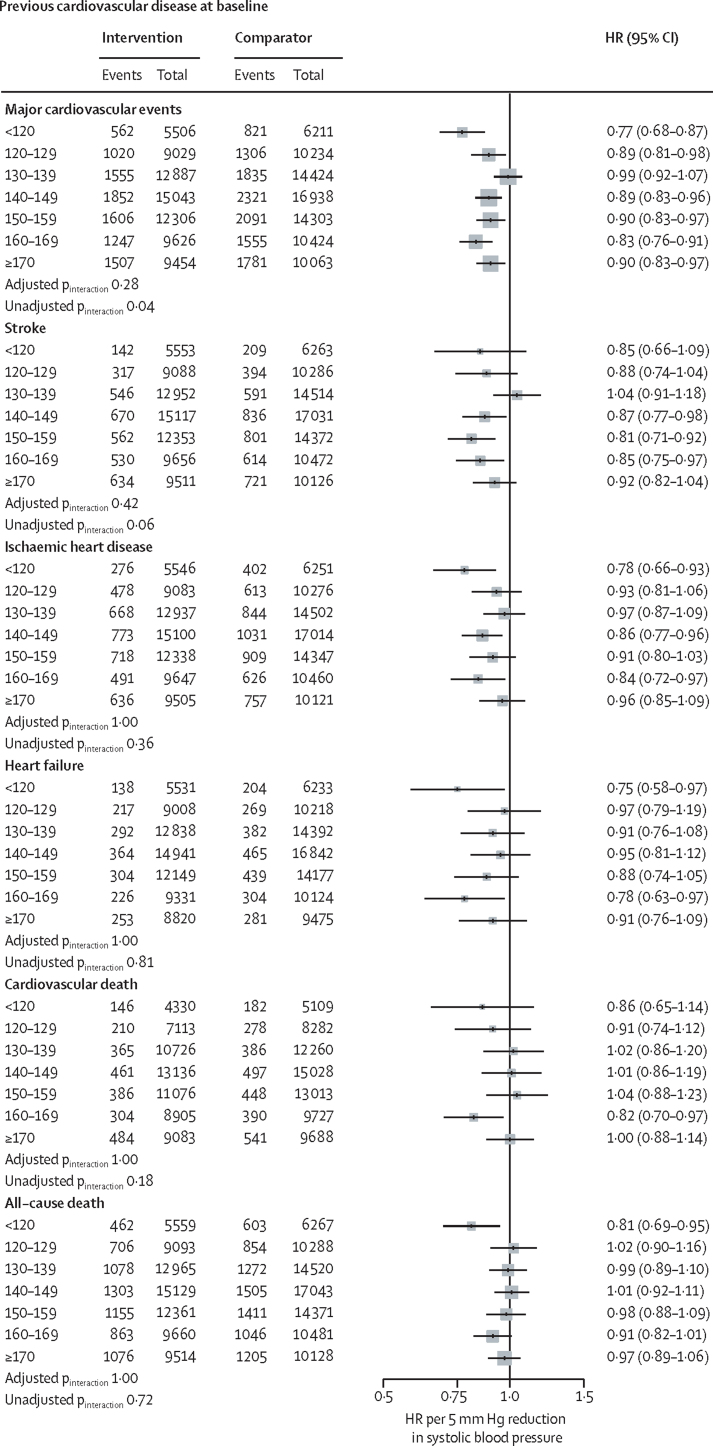

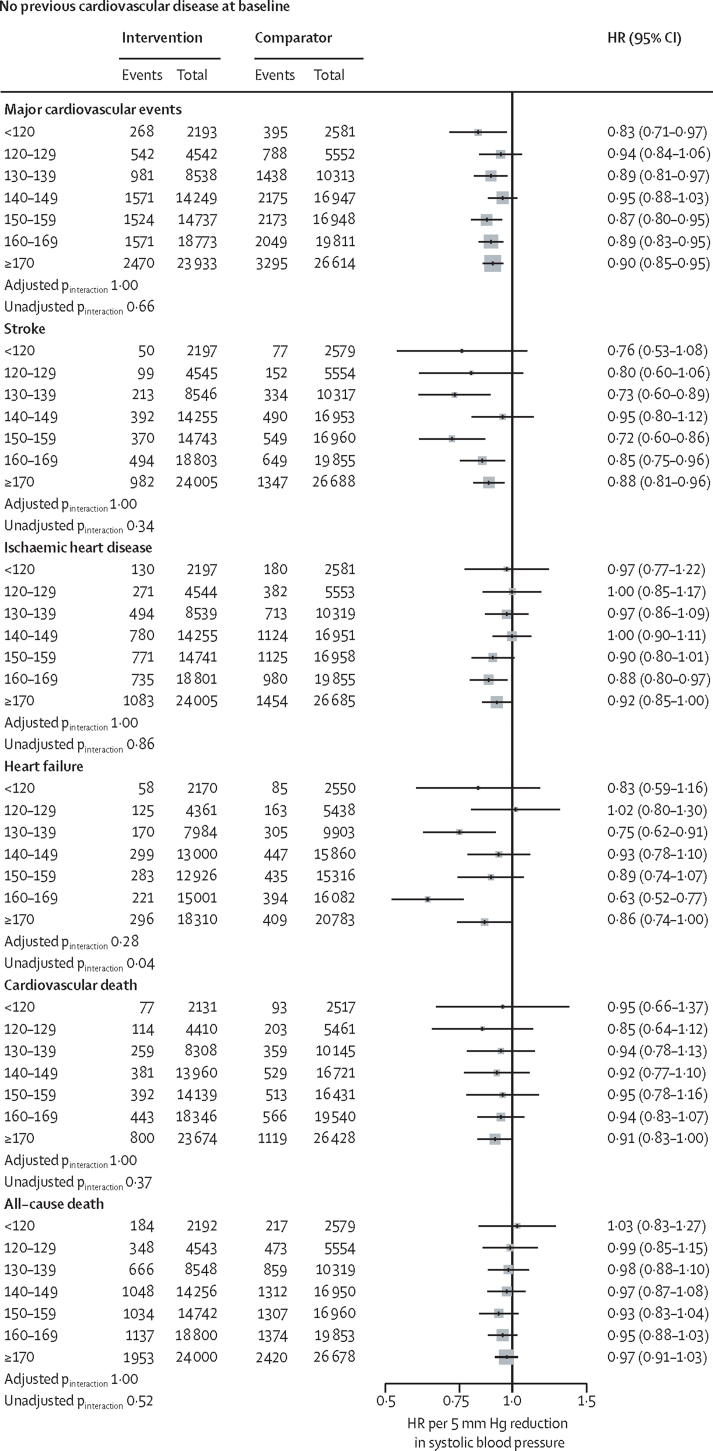
Effects of blood pressure-lowering treatment on primary and secondary outcomes, by cardiovascular disease status and systolic blood pressure at baseline Forest plot shows the HRs and 95% CIs per 5 mm Hg systolic blood pressure reduction, separately for each outcome. Adjusted p_interaction_ values were adjusted for multiple testing using Hommel's method. Unadjusted p_interaction_ values were unadjusted for multiple testing. HR=hazard ratio. CVD=cardiovascular disease.

Figure 4 shows effects stratified by baseline blood pressure categories and cardiovascular disease status; effects stratified by baseline blood pressure, with no subclassification by cardiovascular disease status for primary and secondary outcomes are in the appendix (p 19). Although there was some variation in effect sizes among subgroups, there was no strong statistical evidence of effect modification, suggesting that the variations could be entirely due to chance (all adjusted p_interaction_ >0·28). Additionally, in no subgroup was the HR above 1·0, and there was no pattern of diminishing proportional effects in subgroups with lower (or higher) baseline blood pressure.

When we repeated the analyses stratified by baseline cardiovascular disease status and blood pressure categories unstandardised for blood pressure reduction, the findings were broadly similar to the overall analysis (appendix p 20). There was also no substantial difference in the results after excluding drug class comparison trials in stratified analyses by cardiovascular disease status at baseline (appendix p 21) and stratified by baseline blood pressure categories and cardiovascular disease status (appendix p 22). The findings based on absolute risk reduction further confirmed no observable heterogeneity of effect by cardiovascular disease status and systolic blood pressure categories at baseline (appendix pp 23–24). In the leave-one-out analysis, we found that no single trial was strongly driving the estimations (appendix pp 11–13). The results of the sensitivity analysis based on the two-stage meta-analysis were in keeping with the one-stage method, with the HRs for those with and without previous cardiovascular disease being 0·89 (95% CI 0·87–0·92) and 0·92 (0·90–0·94), respectively. The sensitivity analysis excluding trials with risk of bias did not show any significant change in the effect sizes (appendix p 25). A funnel plot and Egger's regression bias test did not show evidence for the presence of data acquisition bias on the primary outcome (appendix p 26).

## Discussion

In this largest source of randomised evidence of blood pressure-lowering effects on cardiovascular disease and death, we found the proportional effects of blood pressure-lowering on cardiovascular outcomes to be similar in people with or without previous cardiovascular disease and across categories of baseline systolic blood pressure down to less than 120 mm Hg. On average, a 5 mm Hg reduction of systolic blood pressure reduced the risk of a major cardiovascular event by about 10%; the corresponding proportional risk reductions for stroke, heart failure, ischaemic heart disease, and cardiovascular death were 13%, 13%, 8%, and 5%, respectively.

Landmark epidemiological studies have provided compelling evidence for a log-linear relationship between blood pressure and risk of cardiovascular disease across the full physiological blood pressure range, with no threshold below which associations were shown to differ materially.^19^, ^20^ To mitigate the risk of confounding and reverse causality, these studies appropriately excluded people with known cardiovascular disease. However, this selection has contributed to continued controversy over possible thresholds. By 2010, more than 20 studies had reported on the existence of an optimal threshold, albeit with conflicting reports on its exact level and the type of participants and outcomes that might be affected by it.^21^ More recent publications using alternative methods and larger cohorts have not been able to resolve this issue.^22^ Likewise, randomised trials, which have typically either excluded or under-represented participants with normal and high-normal blood pressure, and their tabular meta-analyses, have not provided a reliable answer to the question of treatment effects at lower baseline blood pressure thresholds in people with or without cardiovascular disease.^1^, ^8^, ^23^, ^24^, ^25^

Our study fills the aforementioned gaps in evidence and provides compelling evidence from randomised trials for the beneficial effects of blood pressure-lowering treatment across the spectrum of systolic blood pressure in people with or without a known diagnosis of cardiovascular disease. Our findings do not substantiate concerns about a J-shaped association between blood pressure and cardiovascular outcomes in observational studies, and dismiss the suggestions that blood pressure-lowering treatment is only effective when blood pressure is above a certain threshold.

These findings have important implications for clinical practice. Currently, the approach to prescribing antihypertensives depends on an individual's previous history of cardiovascular disease and blood pressure value. Although guidelines vary on the degree of their emphasis on these two risk factors, they invariably modify recommendations based on them. For instance, New Zealand has largely abandoned the approach of treating hypertension and recommends screening adults for overall cardiovascular risk as the first stage towards clinical decision making.^5^ Nonetheless, in the second stage, those ranked to be at high risk of cardiovascular disease are additionally required to have a high blood pressure to qualify for antihypertensive treatment. Most other guidelines have an even stronger reliance on blood pressure, often with explicit criteria for diagnosis of hypertension in the first stage and then consideration of treatment at the second stage in a subset of hypertensive participants. For instance, in England, blood pressure-lowering treatment for primary prevention of cardiovascular disease is not considered as relevant when baseline systolic blood pressure is less than 140 mm Hg.^4^ Most guidelines also define a floor level for reducing blood pressure, assuming that lowering blood pressure below a common threshold would be ineffective or to have an uncertain or even detrimental effect.

Our study calls for a revision of these guidelines. The finding that a fixed and modest degree of blood pressure reduction is expected to lead to similar relative reductions in risk of cardiovascular events, regardless of current blood pressure or presence of ischaemic heart disease and stroke, calls for consideration of blood pressure-lowering treatment for any individual who has a sufficiently high absolute risk of cardiovascular disease. By considering antihypertensives as a tool for reducing cardiovascular risk, rather than simply reducing blood pressure, clinicians are no longer required to make decisions according to an arbitrary and confusing classification of hypertension. The need for and the burden of exact measurement of blood pressure is also reduced. This will not only simplify decision-making, management, and communication of treatment strategies with participants but, as shown in previous modelling studies, will also lead to more efficient care compared with alternative strategies that rely more heavily on absolute blood pressure values.^26^, ^27^

However, the fact that stratified effects were similar among the phenotypes investigated does not necessarily mean that it is worthwhile treating every patient, or that there is no particular subgroup for whom proportional risk reductions will be greater or smaller. Even in the absence of heterogeneous treatment effects by baseline blood pressure and cardiovascular disease status, clinical decisions for treating raised blood pressure will require consideration of factors such as an individual's overall risk of future cardiovascular events, potential risk of adverse effects, the cost of treatment, and patient preferences.^28^ In this context, our study emphasises the importance of using multivariable risk prediction tools,^27^, ^29^ which have also been shown to be less sensitive to random errors of individual risk factors.^30^

Our results also do not mean that it is appropriate to simply aim for blood pressure reduction to a common threshold for all individuals. Determining the optimal magnitude of blood pressure reduction ideally requires a comparison of varying intensities of blood pressure reduction at differing baseline blood pressure. The design of our study is unable to directly address this question. Rather, we showed that the same fixed level of blood pressure reduction is expected to lead to similar levels of relative risk reduction across a wide range of baseline levels of blood pressure regardless of cardiovascular disease status. In our pre-specified analysis, we report effects for a 5 mm Hg systolic blood pressure reduction. However, as our meta-regression analysis shows, greater relative risk reductions are expected with larger blood pressure reductions, as is the case with multiple blood pressure lowering drugs. Thus, the feasibility of modest systolic blood pressure reductions of about 15 mm Hg across all blood pressure strata, as shown in another BPLTTC study,^10^ together with the consistency of effects shown in the present study, questions the scientific validity of defining a common blood pressure target for all participants.

A key strength of our study is that, with access to the largest IPD resource from randomised trials to date, we were able to stratify the participants simultaneously by baseline cardiovascular disease status and systolic blood pressure, with still several thousand participants included in the lower blood pressure strata. For instance, in those with and without previous cardiovascular disease, 11 839 and 4794, respectively had a baseline systolic blood pressure value of less than 120 mm Hg. Overall, 42 324 had a major cardiovascular event during follow-up, among whom 5720 had a pre-randomisation systolic blood pressure less than 130 mm Hg. This total number of events is about 36% larger than a previous landmark tabular meta-analysis^1^ and, more importantly, five times larger when comparing the subgroups with normal or high-normal systolic blood pressure. Furthermore, unlike tabular meta-analyses, our IPD analysis accounted for loss to follow-up, and, by using a common method for measurement of change in systolic blood pressure over time across all trials, enabled a more consistent and precise adjustment of the differences in blood pressure reduction achieved across the trials.^10^ With this unprecedented investigation of stratified effects, it might be tempting to draw conclusions based on the reported effect sizes for specific subgroups. However, in the absence of strong evidence for an interaction, any variation in effect sizes among subgroups might be entirely due to chance, and therefore it is more appropriate to consider the overall effects as the best estimates of effect for all investigated subgroups.^31^ The lack of heterogeneity of treatment effects was observed on the relative as well as absolute risk scales. The consistency of effects on the absolute risk scale in particular is in line with epidemiological literature showing the limitations of single risk factors of modest strength of association to discriminate risk.^32^ Although this underscores our message of insufficiency of blood pressure and cardiovascular disease status for making decisions about treatment, we caution against using the reported absolute risk differences from clinical trials for making policy decisions. The strength of randomised evidence lies in reporting unbiased relative effects. Measures of absolute risk are better obtained from representative populations, which can then be combined with relative effects from our randomised comparisons to model population-specific absolute risk reductions, as has been shown previously.^26^, ^27^

This study had also some limitations. We stratified analyses based on two variables, which is often insufficient for identifying distinct groups of individuals.^33^ Nonetheless, the phenotypes selected in this study were those with substantial clinical uncertainty and the absence of any meaningful heterogeneity should inform clinical practice as well as future research that seeks to assess the potential heterogeneity of treatment effects based on other important phenotypes, including novel approaches to multivariable and high-dimensional participant stratification.^34^ Relatedly, although we did not exclude any particular participant group other than those with diagnosis of heart failure, some types of groups are likely to have been under-represented or excluded. Thus, we caution against generalising our findings to suggest that treatment is equally effective across all possible patient groups. However, our sensitivity analyses support the robustness of the findings and do not support concerns that inclusion or exclusion of particular trials or alternative assumptions could have changed our conclusions. We did not investigate potential treatment harms. However, as described in the introduction, concerns raised about blood pressure reduction in low blood pressure ranges has been largely due to their disputed unfavourable effects on major cardiovascular outcomes, which our study provides reassurance about. Moreover, our definition of major cardiovascular events did not include other outcomes such as valvular heart disease,^35^, ^36^, ^37^ peripheral vascular disease,^38^ atrial fibrillation^39^, ^40^ or diabetes,^41^ which might also be partially preventable with blood pressure-lowering treatment. Several ongoing and planned BPLTTC studies are investigating such effects which might further help refine treatment recommendations.

This study provides evidence against the widely held view that an individual's blood pressure or previous diagnosis of cardiovascular disease per se are key factors for selecting or deselecting participants for blood pressure-lowering treatment. These findings call for revision of clinical guideline recommendations globally and suggest that antihypertensive medications are better viewed as treatment options for prevention of cardiovascular disease regardless of an individual's blood pressure level and their previous history of cardiovascular disease. For people at risk of cardiovascular disease, pharmacological blood pressure-lowering treatment should become a cornerstone of risk prevention irrespective of cardiovascular disease status or blood pressure.

Correspondence to: Prof Kazem Rahimi, Deep Medicine, Nuffield Department of Women's and Reproductive Health, University of Oxford, Oxford OX1 2BQ, UK kazem.rahimi@wrh.ox.ac.uk

## Data sharing

The governance of the Blood Pressure Lowering Treatment Trialists' Collaboration (BPLTTC) has been reported previously.^11^ The BPLTTC is governed by the University of Oxford's (Oxford, UK) policies on research integrity and codes of practice, and follows the university's policy on the management of research data and records. Scientific activities based on BPLTT datasets are overseen by the BPLTT Steering Committee. All data shared with the BPLTTC will be considered confidential and will not be provided to any third party. Requests for data should be made directly to the data custodians of individual trials. Information about individual projects is posted at https://www.bplltc.org.

## Declaration of interests

MW reports personal fees from Amgen, Kyowa Kirin, and Freeline. MN and DC report grants from the British Heart Foundation (BHF). KR reports grants from BHF, the UK Research and Innovation Global Challenges Research Fund, Oxford Martin School (University of Oxford, Oxford, UK), and National Institute for Health Research Oxford Biomedical Research Centre (University of Oxford); and personal fees from *British Medical Journal Heart* and *Public Library of Science Medicine*. JC reports grants from the National Health and Medical Research Council of Australia. EC, ZB, RR, KKT, CJP, A-CP-G, and BRD declare no competing interests.
