# Department of Error

**DOI:** 10.1016/S0140-6736(21)01069-2

**Published:** 2021-05-22

*The Blood Pressure Lowering Treatment Trialists' Collaboration. Pharmacological blood pressure lowering for primary and secondary prevention of cardiovascular disease across different levels of blood pressure: an individual participant-level data meta-analysis.* Lancet *2021;* **397:** 1625–36—For this Article, The Blood Pressure Lowering Treatment Trialists' Collaboration group list has been updated. This correction has been made to the online version as of May 20, 2021.

