# Supplementary material for: Pharmacological blood pressure lowering for primary and secondary prevention of cardiovascular disease across different levels of blood pressure: an individual participant-level data meta-analysis
Source: Lancet. 2021 May 1;397(10285):1625–36. doi: 10.1016/S0140-6736(21)00590-0 (PMC8102467; doi:10.1016/S0140-6736(21)00590-0)
Supplement: Supplementary appendix [file mmc1.pdf]

THE LANCET

Supplementary appendix

This appendix formed part of the original submission and has been peer reviewed. We post it as supplied by the authors.

Supplement to: The Blood Pressure Lowering Treatment Trialists' Collaboration. Pharmacological blood pressure lowering for primary and secondary prevention of cardiovascular disease across different levels of blood pressure: an individual participant-level data meta-analysis. *Lancet* 2021; **397:** 1625–36.

WEB-ONLY SUPPLEMENT

Pharmacological blood pressure lowering for primary and secondary prevention of cardiovascular disease across different levels of blood pressure: an individual participant-level data meta-analysis

The Blood Pressure Lowering Treatment Trialists' Collaboration

Table of Contents

Trial acronym legend 3

Method S1. Standardisation of proportional effects 4

Table S1. Characteristics of randomised clinical trials included in the analysis. 5

Table S2. Risk of bias assessment of each trial. 9

Table S3. Prevalence of comorbidity by cardiovascular disease status and systolic blood pressure at baseline. 10

Table S4. Leave-one-out sensitivity analysis per 5 mmHg systolic blood pressure reduction for the effects of blood pressure-lowering treatment on primary and secondary outcomes and systolic blood pressure at baseline. 11

Figure S1. Kaplan–Meier rates of stroke per 5 mmHg reduction in systolic blood pressure, stratified by treatment allocation and cardiovascular disease status at baseline. 14

Figure S2. Kaplan–Meier rates of ischaemic heart disease per 5 mmHg reduction in systolic blood pressure, stratified by treatment allocation and cardiovascular disease status at baseline. 15

Figure S3. Kaplan–Meier rates of heart failure per 5 mmHg reduction in systolic blood pressure, stratified by treatment allocation and cardiovascular disease status at baseline. 16

Figure S4. Kaplan–Meier rates of cardiovascular death per 5 mmHg reduction in systolic blood pressure, stratified by treatment allocation and cardiovascular disease status at baseline. 17

Figure S5. Kaplan–Meier rates of all-cause death per 5 mmHg reduction in systolic blood pressure, stratified by treatment allocation and cardiovascular disease status at baseline. 18

Figure S6. Effects of blood pressure-lowering treatment on primary and secondary outcomes by systolic blood pressure at baseline. 19

Figure S7. The unstandardised effects of blood pressure-lowering treatment on primary and secondary outcomes by systolic blood pressure at baseline. 20

Figure S8. Effects of blood pressure-lowering treatment on primary and secondary outcomes, by cardiovascular disease status at baseline, excluding drug classes comparison trials. 21

Figure S9. Effects of blood pressure-lowering treatment on primary and secondary outcomes, by cardiovascular disease status and systolic blood pressure at baseline, excluding drug classes comparison trials. 22

Figure S10. Absolute risk reduction for the effect of blood pressure-lowering treatment on primary and secondary outcomes, by cardiovascular disease status at baseline. 23

Figure S11. Absolute risk reduction for the effect of blood pressure-lowering treatment on primary and secondary outcomes, by cardiovascular disease status and systolic blood pressure at baseline. 24

Table S12. Sensitivity analysis for the effect of blood pressure-lowering treatment on primary and secondary outcomes, stratified by cardiovascular disease status and systolic blood pressure at baseline, excluding the trials with risk of bias. 25

Figure S13. Funnel plot for assessment of publication (acquisition) bias on the effect of blood pressure reduction and risk of major cardiovascular event. 26

References 27

Trial acronym legend

Trial acronym	Full name/Description
**AASK**	African American Study of Kidney Disease and Hypertension
**ABCD**	Appropriate Blood Pressure Control in Diabetes
**ACCORD**	Action to Control Cardiovascular Risk in Diabetes blood pressure trial
**ACTIVE I**	Atrial Fibrillation Clopidogrel Trial with Irbesartan for Prevention of Vascular Events
**ADVANCE**	Action in Diabetes and Vascular disease: preterAx and diamicroN-MR Controlled Evaluation
**ALLHAT**	Antihypertensive and Lipid Lowering Treatment to Prevent Heart Attack Trial
**ANBP**	Australian National Blood Pressure Study
**ANBP2**	Second Australian National Blood Pressure Study
**ASCOT-BPLA**	Anglo-Scandinavian Cardiac Outcomes Trial-Blood Pressure Lowering Arm
**BENEDICT**	BErgamo NEphrologic DIabetes Complications Trial
**CAMELOT**	Comparison of Amlodipine vs Enalapril to Limit Occurrences of Thrombosis
**CAPPP**	Captopril Prevention Project
**Cardio-Sis**	Studio Italiano Sugli Effetti Cardiovascolari del Controllo della Pressione Arteriosa Sistolica
**CASE-J**	Candesartan Antihypertensive Survival Evaluation in Japan Trial
**COLM**	Combination of OLMesartan study
**CONVINCE**	Controlled ONset Verapamil INvestigation of Cardiovascular Endpoints trial
**COPE**	Combination Therapy of Hypertension to Prevent Cardiovascular Events
**DIABHYCAR**	Noninsulin-dependent diabetes, hypertension, microalbuminuria or proteinuria, cardiovascular events, and ramipril
**Dutch TIA Trial**	Dutch Transient Ischemic Attack Trial
**E-COST**	Efficacy of Candesartan on Outcome in Saitama Trial
**ELSA**	European Lacidipine Study on Atherosclerosis
**EUROPA**	EUropean trial on Reduction Of cardiac events with Perindopril in patients with stable coronary Artery disease
**EWPHE**	European Working Party on High Blood Pressure in the Elderly
**HIJ-CREATE**	Heart Institute of Japan Candesartan Randomized Trial for Evaluation in Coronary Artery Disease
**HOMED-BP**	Hypertension Objective Treatment Based on Measurement by Electrical Devices of Blood Pressure
**HOPE**	Heart Outcomes Prevention Evaluation
**HYVET**	Hypertension in the Very Elderly Trial
**IDNT**	Irbesartan Diabetic Nephropathy Trial
**INSIGHT**	International Nifedipine GITS study: Intervention as a Goal in Hypertension Treatment
**INVEST**	International Verapamil-Trandolapril Study
**JMIC-B**	Japan Multicenter Investigation for Cardiovascular Diseases-B
**LIFE**	Losartan Intervention For Endpoint reduction
**MOSES**	Morbidity and Mortality After Stroke, Eprosartan Compared With Nitrendipine for Secondary Prevention
**NICS-EH**	National Intervention Cooperative Study in Elderly Hypertensives
**NORDIL**	Nordic Diltiazem Study
**ONTARGET**	Ongoing Telmisartan Alone and in Combination with Ramipril Global Endpoint Trial
**PART 2**	Prevention of Atherosclerosis with Ramipril Trial
**PEACE**	Prevention of Events with Angiotensin Converting Enzyme Inhibition
**PREVEND IT**	Prevention of Renal and Vascular Endstage Disease Intervention Trial
**PREVENT**	Prospective Randomized Evaluation of the Vascular Effects of Norvasc Trial
**PROFESS**	Prevention Regimen for Effectively Avoiding Second Strokes
**PROGRESS**	Perindopril Protection Against Recurrent Stroke Study
**SHEP**	Systolic Hypertension in the Elderly Program
**SPRINT**	Systolic Blood Pressure Intervention Trial
**STOP Hypertension-2**	Swedish Trial in Old Patients with Hypertension-2
**Syst-Eur**	Systolic Hypertension in Europe
**TRANSCEND**	Telmisartan Randomized Assessment Study in ACE Intolerant Subjects with Cardiovascular Disease
**UKPDS**	UK Prospective Diabetes Study
**VALISH**	Valsartan in Elderly Isolated Systolic Hypertension
**VALUE**	Valsartan Antihypertensive Long-term Use Evaluation
**VHAS**	Verapamil in Hypertension and Atherosclerosis Study

Method S1. Standardisation of proportional effects

Standardisation of effect sizes is appropriate when the aim is pooling the effect of blood pressure-lowering treatment on cardiovascular outcomes and expressing the effect for a fixed level of blood pressure reduction.[1–4] This approach is also underpinned by the fact that relative risk reductions for all major cardiovascular outcomes have been shown to be linearly associated with the magnitude of the blood pressure reductions achieved at a trial level, as evidenced in the meta-regression in this paper and previous work.[3] The added advantage of standardisation is that it enables the inclusion of a wide range of trials of pharmacological blood pressure management without setting an arbitrary threshold for the achieved trial-level of blood pressure reduction.

To determine the level of risk reduction for a uniform change in blood pressure, it is essential to standardise the effect sizes for a predefined and clinically meaningful blood pressure level. The average systolic blood pressure reduction between randomised groups, excluding the first 12 months, amongst all included trials was 6.3 mmHg (95% confidence interval [CI] 6.4 to 6.1).[5] We, therefore, standardised the proportional effect sizes to a 5 mmHg difference in systolic blood pressure between treatment arms, as a convenient round number close to the average blood pressure reduction. More specifically, we used the Cox proportional hazard models, including the following terms:

Treatment*: a binary variable for treatment (0 comparator, 1 intervention);* ***delta****: systolic blood pressure reduction for each trial as continues variable,* ***CVD****: previous cardiovascular disease status (0 non CVD at baseline, 1 CVD at baseline),* ***SBP_cat****: categories of systolic blood pressure at baseline coded as 0 to 7 (<120, 120-129, 130-139, 140-149, 150-159, 160-169, and ≥170 mmHg).*

- Overall model: Treatment + delta + (Treatment × delta)
- Model for prior cardiovascular disease status as interaction: Treatment + delta + (Treatment × delta) + (Treatment × CVD) + (Treatment × delta × CVD)
- Model for systolic blood pressure categories at baseline, in patients with and without prior cardiovascular disease: For this model, we first split the dataset into independent datasets based on previous cardiovascular disease at baseline (CVD). Then we ran the model: Treatment + delta + (Treatment × delta) + (Treatment × SBP_cat) + (Treatment × delta × SBP_cat)

Although other methods have been used to standardise estimates for the intensity of blood pressure reduction in aggregate level, [1–4] in one-stage individual participant data meta-analysis models, the more appropriate approach is including standardisation as part of the main model. In a sensitivity analysis, the standardised estimate of hazard ratio from the two-stage meta-analysis was the same with our chosen one-stage method, supporting the robustness of our one-stage modelling approach.

Table S1. Characteristics of randomised clinical trials included in the analysis.

Trial	Type of trial	Setting	Age (years)	Follow-up duration (years)	Intervention	Comparator	Previous CVD At baseline (CVD/non-CVD)	Definition of Primary outcome	SBP difference (mmHg) excluding first 12 months	Inclusion criteria	Exclusion criteria
			mean (SD)		No. of participants	No. of participants	No. of participants				
**AASK**[6]	Intensive	USA	54 (11)	4.8	More intensive (540)	Less intensive (554)	564/530	MI, Stroke, HF, CVD death	13.0	Age 18-70 years, African-American, hypertension, renal disease (GFR=20-65 ml/min per 1.73m^2)	DBP <95 mmHg, diabetes, urine protein:creatinine ratio >25, recent malignant or hypertension, non-blood pressure-related CKD, serious systemic disease, heart failure
**ABCD**[7]	Intensive	USA	58 (8)	4.7	More intensive (474)	Less intensive (476)	89/861	MI or IHD, Stroke, HF, CVD death	7.7	Age 0-74 years, with T2D, DBP ≥80 mmHg, not on antihypertensive treatment	Recent CAD or CeVD, heart failure, renal disease
**ACCORD**[8]	Intensive	USA and Canada	63 (7)	4.7	More intensive (2362)	Less intensive (2371)	900/3833	IHD or non-fatal MI, Stroke, HF, CVD death	13.9	Age ≥40y years with CVD or ≥50 years with substantial atherosclerosis, T2D, HbA1c ≥7.5%, albuminuria, LVH or ≥2 CVD risk factors (dyslipidaemia, hypertension, smoking, obesity); SBP 130-180 mmHg and taking ≤3 antihypertensive drugs, 24-hour protein excretion rate <1g	Body mass index ≥45 kg/m^2, serum creatinine ≥132.6 μmol/l and other serious illness
**ACTIVE I** [9]	Placebo-controlled	Multi-country	70 (10)	4.1	ARB (3058)	Placebo (3076)	1890/4244	MI, Stroke, HF, CVD death	2.6	Atrial fibrillation, ≥1 risk factor (age ≥75 years, on antihypertensive treatment, history of stroke, TIA or non-CNS embolism, LVEF <45%, PVD, or age 55-74 years with either CAD or diabetes)	Use of anticoagulant, peptic ulcer disease in past 6 months, history of intracerebral haemorrhage, thrombocytopaenia or mitral stenosis
**ADVANCE**[10]	Placebo-controlled	Multi-country	66 (6)	4.2	ACEI and Diuretic (5569)	Placebo (5571)	3461/7679	MI, Stroke, HF, CVD death	5.4	Age ≥55 years T2D (diagnosed aged ≥30y), ≥1 major CVD or ≥1 CVD risk factor (microvascular disease, smoking, dyslipidaemia, microalbuminuria, T2D for ≥10 years, age ≥65 years)	HbA1c target (≤6.5%), definite indication for long-term insulin therapy
**ALLHAT**[11]	Drug classes comparison	Multi-country	67 (8)	4.8	Diuretic (15255)	ACEI, CCB and Alpha-blockers (27163)	5470/36948	MI or IHD, Stroke, HF, CVD death	2.0	Age ≥55y years stage 1 or 2 hypertension plus ≥1 risk factor (MI or stroke >6 months previously, left ventricular hypertrophy, T2D, smoking, HDL <0.91 mmol/l), other atherosclerotic CVD	Symptomatic or hospitalisation for heart failure, LVEF <35%
**ANBP**[12]	Placebo-controlled	Australia	50 (9)	3.6	Diuretic (1721)	Placebo (1706)	16/3411	MI or IHD, Stroke, HF, CVD death	7.5	Age 30-69 years with mild hypertension (DBP 95-110 mmHg and SBP <200 mmHg)	Antihypertensive treatment in past 3 months, recent angina or MI, stroke, hormone therapy, asthma, diabetes, gout, serious disease, tricyclic antidepressant use
**ANBP2**[13]	Drug classes comparison	Australia	73 (5)	4.1	Diuretic (3039)	ACEI (3044)	474/5609	MI or IHD, Stroke, HF, CVD death	0.9	Age 65-84 years, SBP ≥160 mmHg or DBP ≥90 mmHg (if SBP≥140 mmHg), no recent CVD	Serious illness, plasma creatinine >221 μmol/l, malignant hypertension, dementia
**ASCOT-BPLA**[14]	Drug classes comparison	Multi-country	63 (9)	5.3	CCB-based (9639)	Beta-blocker based (9618)	7008/12249	MI or IHD, Stroke, HF, CVD death	2.2	Age 40-79 years, untreated (SBP ≥160 or DBP ≥100 mmHg) or treated hypertension (SBP ≥140 or DBP ≥90 mmHg), ≥3 CVD risk factors (documented LVH, abnormal ECG, T2D, PAD, previous stroke or TIA, male sex, age ≥55 years, microalbuminuria or proteinuria, smoking, TC:HDL ≥6, family history of premature coronary heart disease	Previous MI, current treatment for angina, recent CeVD, fasting triglycerides >4.5 mmol/l, heart failure, arrhythmia, haematological or biochemical abnormality at screening
**BENEDICT**[15]	Placebo-controlled	Italy	62 (8)	3.1	ACEI, CCB and ACEI/CCB (904)	Placebo (300)	Not available	MI or IHD, Stroke, HF, CVD death	1.3	Age ≥40 years, untreated SBP ≥130 / DBP ≥85 mmHg or needing treatment to attain below these levels, T2D for <25 years, urinary albumin excretion rate <20 μg/min, serum creatinine ≤133 μmol/l	HbA1c ≥11%, nondiabetic renal disease
**CAMELOT**[16]	Placebo-controlled	Multi-country	58 (10)	1.6	CCB and ACEI (1340)	Placebo (657)	1858/139	MI, non-fatal Stroke, HF, CVD death	5.3	Age 30-79 years, coronary artery stenosis >20% by angiography, DBP <100 mmHg	Left middle coronary artery obstruction >50%, LVEF <40%, heart failure
**CAPPP**[17]	Drug classes comparison	Sweden and Finland	52 (8)	5.8	Beta-blocker and/or Diuretic (5493)	ACEI (5492)	351/10634	MI or IHD, Stroke, CVD death	2.2	Age 25-66 years, DBP ≥100 mmHg on two occasions	Secondary hypertension, serum creatinine >150 μmol/, condition requiring β-blocker treatment
**CARDIO-SIS**[18]	Intensive	Italy	67 (7)	4.7	More intensive (558)	Less intensive (553)	202/909	MI, Stroke, HF	3.8	Age ≥55 years, SBP ≥150 mmHg, taking antihypertensive drug ≥12 weeks, ≥1 CV risk factor (smoking, dyslipidaemia, family history of premature CVD, prior TIA or stroke, established CAD or PAD	Fasting blood glucose ≥7 mmol/l, diabetes, serious conditions, renal disease, valvular heart disease, left ventricular hypertrophy, atrial fibrillation, substance misuse.
**CASE-J**[19]	Drug classes comparison	Japan	64 (11)	3.1	CCB (2349)	ARB (2354)	1028/3675	MI, Stroke, HF, CVD death	1.7	Age 20-85 years, ≥1 high-risk factor: SBP ≥180 or DBP ≥110 mmHg, T2D, history of angina pectoris, MI, stroke, TIA >6 months prior to screening, LVH, proteinuria or serum creatinine ≥1.3 mg/100 ml, peripheral artery obstruction	BP ≥200/120 mmHg, T1D, heart failure, ejection fraction <40%, atrial fibrillation, cancer
**COLM**[20]	Drug classes comparison	Japan	74 (5)	3.0	ARB and Diuretic (2573)	ARB and CCB (2568)	1225/3916	MI, Stroke, HF, CVD death	0.3	Age 65-84 years, hypertension (treated: BP ≥140/90 mmHg; untreated: BP ≥160/100 mmHg), CVD history or CVD risk factors (diabetes, dyslipidaemia)	Secondary/malignant hypertension, recent major CVD, revascularisation, angina pectoris hospitalisation or severe heart failure, atrial fibrillation, hepatic or renal dysfunction

**CONVINCE**[21]	Drug classes comparison	Multi-country	66 (7)	2.8	CCB (8179)	Beta-blocker or Diuretic (8297)	4458/12018	MI or IHD, Stroke, HF, CVD death	0.0	Age ≥55 years, hypertension, ≥1 CVD risk factor (e.g., diabetes, smoking)	Heart failure, dysrhythmia, secondary hypertension, recent MI or stroke, renal disease, other serious disease, BP ≥190/110 mmHg without treatment
**COPE**[22]	Drug classes comparison	Japan	64 (11)	3.6	CCB/Diuretic and CCB/ Beta-blocker (2183)	CCB and ARB (1110)	219/3074	MI, Stroke, HF, CVD death	0.4	Age 40-85 years, BP ≥140/90 mmHg	SBP ≥200 or DBP ≥120 mmHg, secondary hypertension, diabetes, recent CVD or revascularisation, heart failure, atrial fibrillation/flutter, hepatic or renal dysfunction, congenital or rheumatic heart disease, cancer
**DIABHYCAR**[23]	Placebo-controlled	Multi-country	65 (8)	3.9	ACEI (2443)	Placebo (2469)	739/4173	MI, Stroke, HF, CVD death	0.9	Age ≥50 years, T2D, urinary albumin excretion ≥20 mg/l in two consecutive urine samples	Serum creatinine >150 μmol/l, use of insulin, ACEI or ARB, heart failure, recent MI, urinary tract infection
**Dutch TIA Trial**[24]	Placebo-controlled	The Netherlands	64 (10)	2.3	Beta-blocker (732)	Placebo (741)	1473/0	non-fatal MI or IHD, Stroke, CVD death	3.1	TIA or non-disabling ischaemic stroke (Rankin Scale ≤3) in past 3 months	Cerebral ischaemia from identifiable causes other than arterial thrombosis or embolism
**E-COST**[25]	Drug classes comparison	Japan	64 (11)	3.1	ARB (1053)	Conventional (995)	213/1835	Not available	Not available	Age 35-79 years, BP 140-180/90-110 mmHg	Diabetes, dysglycemia, secondary hypertension, recent MI or stroke, angina pectoris requiring β-blocker treatment, heart failure, left ventricular ejection fraction <40%
**ELSA**[26]	Drug classes comparison	Multi-country	57 (7)	3.4	CCB (1177)	Beta-blocker (1157)	305/2029	MI or IHD, Stroke, HF, CVD death	0.8	Age 45-79 years, BP 150-210/95-115 mmHg	Recent MI or stroke, and T2D
**EUROPA**[27]	Placebo-controlled	Multi-country (Europe)	61 (9)	4.2	ACEI (6110)	Placebo (6108)	12218/0	MI, Stroke, HF	4.6	Age ≥18 years, documented MI >3 months before screening, revascularisation >6 months before screening, >70% coronary obstruction	Heart failure, hypotension, uncontrolled hypertension, renal insufficiency, serum potassium >5.5 mmol/L
**EWPHE**[28]	Placebo-controlled	Multi-country	71 (8)	4.6	Diuretic (416)	Placebo (424)	124/716	MI, Stroke, HF, CVD death	22.4	Age ≥60 years, BP 160-239/90-119 mmHg	Curable causes of high BP, retinopathy, heart failure, stroke history, hepatitis/cirrhosis, gout, malignancy, diabetes requiring insulin treatment
**HIJ-CREATE**[29]	Drug classes comparison	Japan	65 (9)	4.0	ARB (1024)	non-ARB (1025)	2049/0	MI, Stroke, HF, CVD death	0.4	Age 20-80 years, CAD hospitalisation and hypertension (BP ≥140/90 mmHg or antihypertensive treatment use)	Secondary hypertension, recent AMI or CeVD, severe aortic valve stenosis, cardiomyopathy, serum creatinine >2 mg/dl, serum potassium >5 mmol/l, hepatic dysfunction, malignancy
**HOMED-BP**[30]	Intensive	Japan	60 (10)	4.9	More intensive (1759)	Less intensive (1759)	106/3412	MI, Stroke, HF, CVD death	2.0	Self-measured SBP 135-179 mmHg or DBP 85-119 mmHg, but not if DBP <65 or SBP <110 mmHg (clinic SBP <220 mmHg and DBP <125 mmHg)	None specified
**HOPE**[31]	Placebo-controlled	Multi-country	66 (7)	4.5	ACEI (4645)	Placebo (4652)	7477/1820	MI, Stroke	3.0	Age ≥55 years, CAD, stroke, PVD or diabetes, plus ≥1 risk factor (hypertension, dyslipidaemia, smoking, or documented microalbuminuria)	Heart failure, left ejection fraction <40%, using ACEI or Vitamin E, uncontrolled hypertension, nephropathy, or recent MI or stroke
**HYVET**[32]	Placebo-controlled	Multi-country	84 (3)	2.1	Diuretic (1933)	Placebo (1912)	374/3471	MI, Stroke, HF, CVD death	13.1	Age ≥80y years, sustained SBP ≥160 mmHg	Accelerated or secondary hypertension, recent haemorrhagic stroke, heart failure, serum creatinine >150 μmol/L, serum potassium <3.5 or >5.5 mmol/L, gout, and dementia
**IDNT**[33]	Placebo-controlled	USA	59 (8)	2.6	ARB and CCB (1146)	Placebo (569)	Not available	Not available	2.8	Age 30-70 years, T2D, hypertension (BP ≥135/85 mmHg or taking anti-hypertensive drug), proteinuria, serum creatinine (μmol/l): 88 to 265 (women) or 106 to 265 (men)	None specified
**INSIGHT**[34]	Drug classes comparison	Multi-country	65 (6)	2.8	Diuretic (3164)	CCB (3157)	671/5650	MI, Stroke, fatal HF, CVD death	1.1	Age 55-80 years, hypertensive (SBP ≥150 or DBP ≥95 mmHg, or SBP ≥160 mmHg), ≥1 other risk factor (TC ≥6.43 mmol/l, smoking, family history of premature MI, CAD, other CVD	None specified
**INVEST**[35]	Drug classes comparison	Multi-country	66 (10)	2.8	CCB (10648)	non-CCB (10672)	21320/0	MI or IHD, Stroke, HF, CVD death	0.1	Age ≥50 years, documented CAD, essential hypertension requiring drug therapy, heart failure Class I-III[b]	Patients taking β-blocker within two weeks of randomization or for recent MI
**JMIC-B**[36]	Drug classes comparison	Japan	65 (85)	2.3	CCB (828)	ACEI (822)	1650/0	MI or IHD, Stroke, HF, CVD death	2.0	Age <75 years, hypertension (BP ≥160/≥95 mmHg or both SBP ≥150 and DBP ≥90 mmHg, or antihypertensive treatment), CAD or meeting both criteria: history of >2 anginal attacks per week with stable frequency and ST-segment depression of ≥1 mm on stress test (or detection of MI with myocardial scintigraphy)	MI, unstable angina, DBP ≥120 mmHg, secondary hypertension, symptomatic CeVD, heart failure, atrial fibrillation/arrhythmias, renal or hepatic dysfunction, uncontrollable diabetes and familial hypercholesterolaemia
**LIFE**[37]	Drug classes comparison	Multi-country	67 (7)	4.9	ARB (4605)	Beta-blocker (4588)	1771/7422	MI or IHD, Stroke, HF, CVD death	1.2	Age 55-80 years, hypertension (SBP 160-200 mmHg; DBP 95-115 mmHg), electrocardiogram signs of LVH	Secondary hypertension, recent MI or stroke, angina pectoris requiring treatment, heart failure or left ejection fraction ≤40%
**MOSES**[38]	Drug classes comparison	Germany and Austria	68 (10)	3.3	CCB (671)	ARB (681)	1352/0	MI or IHD, Stroke, HF, CVD death	1.5	Hypertension requiring treatment, documented TIA, ischaemic stroke or cerebral haemorrhage	Internal carotid artery occlusion or stenosis >70%, heart failure, age >85 years, on anticoagulant for cardiac arrhythmia, high-grade aortic or mitral valve stenosis, unstable angina
**NICS-EH**[39]	Drug classes comparison	Japan	70 (7)	3.2	Diuretic (214)	CCB (215)	16/401	MI or IHD, Stroke, HF, CVD death	0.3	Age ≥60 years, SBP 160-220 mmHg and DBP <115 mmHg and no cardiovascular complications	None specified

**NORDIL**[40]	Drug classes comparison	Norway and Sweden	60 (7)	4.2	Beta-blocker and/or Diuretic (5471)	CCB (5410)	740/10141	MI or IHD, Stroke, CVD death	3.3	Age 50-74 years, untreated hypertension (DBP ≥100 mmHg on two occasions); if previously treated, DBP ≥100 mmHg on two consecutive visits at one week apart during run-in period and no treatment was given	Age <50 or ≥70y, bradycardia, secondary hypertension, atrial fibrillation, recent CeVD or MI, heart failure
**ONTARGET**[41]	Drug classes comparison	Multi-country	67 (7)	4.8	ARB/ACEI (8502)	ACEI and ARB (17118)	22315/3301	MI or IHD, Stroke, HF, CVD death	1.9	CAD, PAD, CeVD or diabetes with end-organ damage	Heart failure, pericarditis, congenital heart disease, unexplained syncope , planned revascularisation <3 months of consent, uncontrolled hypertension, heart transplant, subarachnoid haemorrhage, renal artery disease, proteinuria, hepatic dysfunction, volume or sodium depletion, primary hyper-aldosteronism, hereditary fructose intolerance, other serious conditions
**PART 2**[42]	Placebo-controlled	New Zealand	60 (8)	4.6	ACEI (308)	Placebo (309)	457/160	MI or IHD, Stroke, HF, CVD death	6.5	Age ≤75 years, diagnosis (in past 5 year) of MI, documented CAD, TIA or intermittent claudication	Heart failure, serious nonvascular disease, SBP >160 mmHg, DBP >100 mm Hg, DBP <100 mmHg during pre-randomization run-in period
**PEACE**[43]	Placebo-controlled	Multi-country (USA, Puerto Rico, Canada and Italy)	64 (8)	4.7	ACEI (4158)	Placebo (4132)	8290/0	non-fatal MI, non-fatal stroke, HF, CVD death	3.0	Age ≥50 years, documented CAD	Unstable angina, severe valvular heart disease, recent revascularisation, planned elective revascularisation, limited 5-year survival, serum creatinine >177 μmol/l, serum potassium >5.5 mmol/l
**PREVEND IT**[44]	Placebo-controlled	The Netherlands	51 (12)	3.8	ACEI (431)	Placebo (433)	24/840	MI, Stroke, HF, CVD death	5.6	Microalbuminuria, SBP <160/100 mmHg (no previous antihypertension treatment)	Creatinine clearance <60% of normal age-adjusted value
**PREVENT**[45]	Placebo-controlled	USA and Canada	57 (10)	3.0	CCB (417)	Placebo (408)	825/0	MI, Stroke, HF, CVD death	6.1	Age 30-80 years, documented CAD, DBP <95 mmHg, cholesterol <325 mg/dl, fasting blood glucose <200 mg/dl	Contraindication for dihydropyridines, uncontrolled hypertension, diabetes and other major illness
**PROFESS**[46]	Placebo-controlled	Multi-country	66 (8)	2.5	ARB (9873)	Placebo (9925)	19798/0	MI or IHD, Stroke, HF, CVD death	3.4	Age ≥55 years with ischaemic stroke <90 days before randomization (later modified to include age 50 to 54 years or had stroke 90 to 120 days before randomisation if with ≥2 additional risk factors: diabetes, hypertension, smoker, obesity previous CVD, end-organ damage or hyperlipidaemia) and remained stable	Haemorrhagic stroke, severe disability after the qualifying stroke, contraindication to treatments
**PROGRESS**[47]	Placebo-controlled	Multi-country (Asia, Australasia, Europe)	64 (10)	3.9	ACEI and/or Diuretic (3051)	Placebo (3054)	6105/0	MI or IHD, Stroke, HF, CVD death	9.2	Stroke or TIA in past 5 years	Indication or contraindication for ACEI
**SHEP**[48]	Placebo-controlled	USA	72 (7)	5.0	Beta-blocker and Diuretic (2365)	Placebo (2371)	284/4441	non-fatal MI, non-fatal Stroke, CVD death	12.8	Age ≥60 years, isolated systolic hypertension (BP 160-219/<90 mmHg, not on treatment)	Major CVD, cancer, alcoholic liver disease, renal dysfunction, competing risk of SHEP primary endpoint or presence of medical management exclusions
**SPRINT**[49]	Intensive	USA and Puerto Rico	68 (9)	3.0	More intensive (4678)	Less intensive (4683)	1877/7484	MI or IHD, Stroke, HF, CVD death	14.9	Age ≥50y years, SBP 130-180 mmHg, increased CVD risk (clinical/subclinical CVD other than stroke, CKD excluding polycystic kidney disease and with eGFR of 20-60 ml/min/1.73m^2 body surface area, 10-year Framingham CVD risk ≥15%, age ≥75y)	Diabetes or prior stroke
**STOP Hypertension-2**[50]	Drug classes comparison	Sweden	76 (4)	4.5	Beta-blocker and/or Diuretic (2213)	ACEI and CCB (4401)	1072/5542	MI or IHD, Stroke, HF, CVD death	2.1	Aged 70-84 years, SBP ≥180 mmHg and/or DBP ≥105 mmHg	Not specified
**SYST-EUR**[51]	Placebo-controlled	Multi-country	70 (7)	2.6	CCB (2398)	Placebo (2297)	286/4409	MI or IHD, Stroke, HF, CVD death	10.1	Age ≥60 years, sitting SBP 160-219 mmHg, sitting DBP <95 mmHg, and standing SBP ≥140 mmHg	Secondary hypertension, retinal haemorrhage/papilloedema, heart failure, dissecting aortic aneurysm, serum creatinine ≥180 μmol/l, recent severe nosebleeds, stroke or MI, dementia, disorders prohibiting standing position, severe CVD/non-CVD
**TRANSCEND**[52]	Placebo-controlled	Multi-country	68 (7)	4.9	ARB (2954)	Placebo (2972)	5222/701	MI or IHD, Stroke, HF, CVD death	4.5	Intolerant to ACEI and with established CAD, PVD, CeVD or diabetes with end-organ damage	Heart failure, valvular/cardiac outflow tract obstruction, pericarditis, congenital heart disease, unexplained syncope, recent revascularisation, SBP >160 mmHg, heart transplantation, subarachnoid haemorrhage, significant renal stenosis, renal or hepatic dysfunction
**UKPDS**[53]	Intensive	UK	56 (8)	7.9	More intensive (758)	Less intensive (390)	35/1113	MI or IHD, Stroke	11.2	Age 25-65 years, newly diagnosed diabetes, and hypertension (untreated: SBP ≥160 mmHg and/or DBP ≥90 mmHg; treated: SBP ≥150 mmHg and/or DBP ≥85 mmHg)	Ketonuria, recent MI, angina, heart failure, >1 major vascular episode, serum creatinine >15 μmol/, retinopathy, malignant hypertension, uncorrected endocrine abnormality, severe concurrent illness
**VALISH**[54]	Intensive	Japan	76 (4)	2.6	More intensive (1545)	Less intensive (1534)	371/2708	MI, Stroke, HF, CVD death	5.0	Age ≥70 to <85 years, isolated hypertension (SBP >160 mmHg and DBP <90 mmHg)	Secondary or malignant hypertension, BP ≥200/≥90 mmHg, recent CeVD or MI, recent/planned revascularisation, heart failure, aortic stenosis, valvular heart disease, atrial fibrillation/flutter, serious arrhythmia, renal/liver dysfunction

**VALUE**[55]	Drug classes comparison	Multi-country	67 (8)	4.2	CCB-based (7596)	ARB-based (7649)	9169/6076	MI, Stroke, HF, CVD death	1.6	Age ≥50 years, hypertension, CVD, CVD risk factors (male sex, age >50 years, diabetes, current smoking, high cholesterol, LVH, proteinuria, serum creatinine 150 to 265 μmol/l)	Renal artery stenosis, recent CAD or CeVD, severe hepatic disease or chronic renal failure, heart failure, on monotherapy with β-blocker for CAD and hypertension
**VHAS**[56]	Drug classes comparison	Italy	54 (7)	1.7	Diuretic (707)	CCB (707)	0/1249	MI or IHD, Stroke, HF, CVD death	1.7	Age 40-65 years, BP ≥160/95 mmHg	Secondary hypertension, recent stroke or TIA, CAD, PAD, bradycardia, arrhythmias, heart failure, renal or hepatic dysfunction, hyperuricaemia, hypokalemia, T1D, familial dyslipidemia, serious concomitant disease

CVD: Cardiovascular disease; MI: myocardial infraction; IHD: ischaemic heart disease; HF: heart failure; SD: Standard deviation; ACEI, angiotensin-converting enzyme inhibitor; ARB, angiotensin receptor blocker; BB, beta-blocker; CCB, calcium channel blocker.

Table S2. Risk of bias assessment of each trial.						
**Trial**	**Risk of bias arising from randomisation**	**Risk of bias due to effect of assignment to intervention**	**Risk of bias due to missing outcome data**	**Risk of bias due to measurement of outcome**	**Risk of bias due to reporting of result**	**Overall risk of bias**
AASK	Low	Low	Low	Low	Low	Low
ABCD	Low	Low	Low	Low	Low	Low
ACCORD	Low	Some	Low	Low	Low	Low
ACTIVE I	Low	Low	Low	Low	Low	Low
ADVANCE	Low	Low	Low	Low	Low	Low
ALLHAT	Low	Low	Low	Low	Low	Low
ANBP	Low	Low	Low	Low	Low	Low
ANBP2	Low	Some	Low	Low	Low	Low
ASCOT-BPLA	Low	Some	Low	Low	Low	Low
CAMELOT	Low	Low	Low	Low	Low	Low
CAPPP	Low	Some	Low	Low	Low	Some
CARDIO-SIS	Low	Some	Some	Low	Low	Some
CASE-J	Low	Some	Low	Low	Low	Low
COLM	Low	Some	Low	Low	Low	Low
CONVINCE	Low	Low	Low	Low	Low	Low
COPE	Low	Some	Low	Low	Low	Low
DIABHYCAR	Low	Low	Low	Low	Low	Low
Dutch TIA Trial	Low	Low	Low	Low	Low	Low
ELSA	Low	Low	Low	Low	Low	Low
EUROPA	Low	Low	Low	Low	Low	Low
EWPHE	Low	Some	Low	Low	Low	Some
HIJ-CREATE	Low	Some	Low	Low	Low	Low
HOMED-BP	Low	Some	Low	Low	Low	Low
HOPE	Low	Some	Low	Low	Low	Low
HYVET	Low	Low	Low	Low	Low	Low
INSIGHT	Low	Low	Low	Low	Low	Low
INVEST	Low	Some	Low	Low	Low	Low
JMIC-B	Low	Some	Low	Low	Low	Low
LIFE	Low	Low	Low	Low	Low	Low
MOSES	Low	Some	Low	Low	Low	Low
NICS-EH	Low	Some	Low	Low	Low	Some
NORDIL	Low	Some	Low	Low	Low	Low
ONTARGET	Low	Some	Low	Low	Low	Low
PART 2	Low	Low	Low	Low	Low	Low
PEACE	Low	Low	Low	Low	Low	Low
PREVEND IT	Low	Low	Low	Low	Low	Low
PREVENT	Low	Low	Low	Low	Low	Low
PROFESS	Low	Low	Low	Low	Low	Low
PROGRESS	Low	Low	Low	Low	Low	Low
SHEP	Low	Low	Low	Low	Low	Low
SPRINT	Low	Some	Low	Low	Low	Low
STOP HYPERTENSION-2	Low	Some	Low	Low	Low	Low
SYST-EUR	Low	Some	Low	Low	Low	Low
TRANSCEND	Low	Low	Low	Low	Low	Low
UKPDS	Low	Some	Low	Low	Low	Low
VALISH	Low	Low	Low	Low	Low	Low
VALUE	Low	Low	Low	Low	Low	Low
VHAS	Low	Low	Low	Low	Low	Low

Trial name acronyms are described in full in the Trial acronym legend in the Supplement.

Table S3. Prevalence of comorbidity by cardiovascular disease status and systolic blood pressure at baseline.

	No previous cardiovascular disease at baseline							Previous cardiovascular disease at baseline						
	Baseline systolic blood pressure							Baseline systolic blood pressure						
**Comorbidity**	**<120**	**120-129**	**130-139**	**140-149**	**150-159**	**160-169**	**≥170**	**<120**	**120-129**	**130-139**	**140-149**	**150-159**	**160-169**	**≥170**
	n (%)	n (%)	n (%)	n (%)	n (%)	n (%)	n (%)	n (%)	n (%)	n (%)	n (%)	n (%)	n (%)	n (%)
**Peripheral vascular disease**	78 (7.6)	188 (9.9)	332 (9.7)	590 (7.2)	645 (6.3)	825 (5.3)	1275 (5.9)	640 (13.3)	1063 (13.2)	1484 (13.0)	1731 (11.9)	1466 (11.9)	1259 (12.2)	1314 (11.9)
**Atrial fibrillation**	465 (9.7)	866 (8.5)	1077 (5.7)	1117 (3.6)	765 (2.4)	608 (1.6)	905 (1.8)	451 (3.8)	753 (3.9)	937 (3.4)	927 (2.9)	713 (2.7)	446 (2.2)	449 (2.3)
**Diabetes**	2163 (45.1)	4255 (42.0)	7747 (40.9)	11514 (36.8)	10485 (32.9)	9214 (23.7)	9812 (19.3)	2691 (25.4)	4310 (25.6)	6825 (27.7)	8485 (28.7)	7219 (28.6)	5113 (26.5)	5063 (26.2)
**Chronic kidney disease**	501 (40.0)	569 (23.4)	1205 (19.7)	2847 (21.5)	2926 (19.3)	3537 (17.3)	4574 (17.6)	134 (4.4)	214 (3.8)	472 (5.7)	1135 (9.5)	1156 (11.3)	1307 (14.6)	1673 (18.8)
**Cerebrovascular disease**	0 (0.0)	0 (0.0)	0 (0.0)	0 (0.0)	0 (0.0)	0 (0.0)	0 (0.0)	2326 (22.8)	5445 (31.8)	8468 (34.6)	10437 (36.3)	8991 (37.5)	7339 (40.2)	7390 (41.1)
**Ischaemic heart disease**	0 (0.0)	0 (0.0)	0 (0.0)	0 (0.0)	0 (0.0)	0 (0.0)	0 (0.0)	10264 (86.7)	15328 (79.0)	21144 (76.8)	24286 (75.4)	19989 (74.7)	14464 (71.7)	13851 (70.4)
**Heart failure**	0 (0.0)	0 (0.0)	0 (0.0)	0 (0.0)	0 (0.0)	0 (0.0)	0 (0.0)	0 (0.0)	0 (0.0)	0 (0.0)	0 (0.0)	0 (0.0)	0 (0.0)	0 (0.0)

n: number of participants

Table S4. Leave-one-out sensitivity analysis per 5 mmHg systolic blood pressure reduction for the effects of blood pressure-lowering treatment on primary and secondary outcomes and systolic blood pressure at baseline.

	Trial excluded from analysis															
Baseline systolic blood pressure	AASK	ABCD	ACCORD	ACTIVE I	ADVANCE	ALLHAT	ANBP	ANBP2	ASCOT-BPLA	CAMELOT	CAPPP	CARDIO-SIS	CASE-J	COLM	CONVINCE	COPE
**Major cardiovascular events**	**HR 95%CI**	**HR 95%CI**	**HR 95%CI**	**HR 95%CI**	**HR 95%CI**	**HR 95%CI**	**HR 95%CI**	**HR 95%CI**	**HR 95%CI**	**HR 95%CI**	**HR 95%CI**	**HR 95%CI**	**HR 95%CI**	**HR 95%CI**	**HR 95%CI**	**HR 95%CI**
<120	0.79 (0.72 to 0.87)	0.79 (0.72 to 0.87)	0.79 (0.71 to 0.89)	0.78 (0.71 to 0.86)	0.77 (0.67 to 0.85)	0.78 (0.70 to 0.86)	0.79 (0.72 to 0.87)	0.79 (0.72 to 0.87)	0.79 (0.72 to 0.87)	0.79 (0.72 to 0.87)	0.79 (0.72 to 0.87)	0.79 (0.72 to 0.87)	0.79 (0.72 to 0.87)	0.79 (0.72 to 0.87)	0.79 (0.72 to 0.87)	0.79 (0.72 to 0.87)
120 to 129	0.91 (0.85 to 0.98)	0.91 (0.85 to 0.98)	0.91 (0.84 to 0.98)	0.92 (0.85 to 0.99)	0.90 (0.83 to 0.97)	0.91 (0.84 to 0.98)	0.91 (0.85 to 0.98)	0.91 (0.85 to 0.98)	0.91 (0.85 to 0.98)	0.92 (0.85 to 0.99)	0.91 (0.85 to 0.98)	0.91 (0.85 to 0.98)	0.91 (0.85 to 0.98)	0.91 (0.85 to 0.98)	0.92 (0.85 to 0.99)	0.91 (0.85 to 0.98)
130 to 139	0.94 (0.89 to 1.00)	0.94 (0.89 to 0.99)	0.91 (0.85 to 0.97)	0.93 (0.88 to 0.97)	0.93 (0.88 to 0.98)	0.97 (0.91 to 1.03)	0.94 (0.89 to 0.99)	0.94 (0.89 to 0.99)	0.94 (0.89 to 0.99)	0.94 (0.89 to 1.00)	0.94 (0.89 to 1.00)	0.94 (0.89 to 1.00)	0.94 (0.89 to 1.00)	0.94 (0.89 to 1.00)	0.94 (0.89 to 1.00)	0.94 (0.89 to 0.99)
140 to 149	0.92 (0.88 to 0.98)	0.93 (0.88 to 0.98)	0.90 (0.85 to 0.96)	0.93 (0.88 to 0.98)	0.94 (0.89 to 0.99)	0.91 (0.86 to 0.96)	0.93 (0.88 to 0.98)	0.93 (0.88 to 0.98)	0.94 (0.89 to 0.99)	0.93 (0.88 to 0.98)	0.93 (0.88 to 0.98)	0.93 (0.88 to 0.98)	0.93 (0.88 to 0.98)	0.93 (0.88 to 0.98)	0.93 (0.88 to 0.98)	0.93 (0.88 to 0.98)
150 to 159	0.88 (0.83 to 0.93)	0.88 (0.83 to 0.94)	0.89 (0.83 to 0.95)	0.89 (0.83 to 0.94)	0.87 (0.83 to 0.92)	0.88 (0.83 to 0.94)	0.88 (0.84 to 0.94)	0.88 (0.83 to 0.94)	0.88 (0.83 to 0.94)	0.88 (0.83 to 0.94)	0.89 (0.84 to 0.94)	0.89 (0.84 to 0.94)	0.89 (0.84 to 0.94)	0.89 (0.83 to 0.94)	0.89 (0.83 to 0.94)	0.89 (0.83 to 0.94)
160 to 169	0.87 (0.82 to 0.92)	0.87 (0.82 to 0.92)	0.87 (0.83 to 0.92)	0.87 (0.83 to 0.92)	0.86 (0.82 to 0.91)	0.87 (0.82 to 0.92)	0.87 (0.82 to 0.92)	0.87 (0.82 to 0.92)	0.89 (0.85 to 0.94)	0.87 (0.83 to 0.92)	0.87 (0.82 to 0.92)	0.87 (0.83 to 0.92)	0.87 (0.83 to 0.92)	0.87 (0.82 to 0.92)	0.87 (0.82 to 0.91)	0.87 (0.83 to 0.92)
≥170	0.90 (0.86 to 0.94)	0.90 (0.86 to 0.94)	0.91 (0.87 to 0.95)	0.90 (0.86 to 0.95)	0.90 (0.86 to 0.95)	0.90 (0.86 to 0.94)	0.90 (0.87 to 0.95)	0.90 (0.86 to 0.94)	0.92 (0.88 to 0.97)	0.90 (0.86 to 0.94)	0.90 (0.86 to 0.94)	0.90 (0.86 to 0.94)	0.90 (0.86 to 0.94)	0.90 (0.87 to 0.95)	0.90 (0.86 to 0.94)	0.90 (0.86 to 0.94)
p for interaction	P adjusted=0.35	P adjusted=0.35	P adjusted=0.77	P adjusted=0.28	P adjusted=0.14	P adjusted=0.14	P adjusted=0.28	P adjusted=0.27	P adjusted=0.28	P adjusted=0.35	P adjusted=0.35	P adjusted=0.35	P adjusted=0.35	P adjusted=0.28	P adjusted=0.35	P adjusted=0.28
	P unadjusted=0.05	P unadjusted=0.05	P unadjusted=0.11	P unadjusted=0.04	P unadjusted=0.02	P unadjusted=0.02	P unadjusted=0.04	P unadjusted=0.03	P unadjusted=0.04	P unadjusted=0.05	P unadjusted=0.05	P unadjusted=0.05	P unadjusted=0.05	P unadjusted=0.04	P unadjusted=0.05	P unadjusted=0.04
**Stroke**	**HR 95%CI**	**HR 95%CI**	**HR 95%CI**	**HR 95%CI**	**HR 95%CI**	**HR 95%CI**	**HR 95%CI**	**HR 95%CI**	**HR 95%CI**	**HR 95%CI**	**HR 95%CI**	**HR 95%CI**	**HR 95%CI**	**HR 95%CI**	**HR 95%CI**	**HR 95%CI**
<120	0.81 (0.66 to 1.00)	0.79 (0.64 to 0.96)	0.83 (0.67 to 1.03)	0.77 (0.63 to 0.95)	0.75 (0.60 to 0.92)	0.79 (0.64 to 0.96)	0.79 (0.65 to 0.97)	0.79 (0.65 to 0.97)	0.80 (0.65 to 0.97)	0.80 (0.66 to 0.98)	0.80 (0.66 to 0.98)	0.80 (0.65 to 0.97)	0.80 (0.65 to 0.97)	0.79 (0.65 to 0.97)	0.79 (0.65 to 0.97)	0.79 (0.65 to 0.97)
120 to 129	0.87 (0.76 to 1.01)	0.86 (0.74 to 0.99)	0.86 (0.74 to 0.99)	0.88 (0.76 to 1.01)	0.85 (0.73 to 0.98)	0.86 (0.74 to 0.99)	0.86 (0.74 to 0.99)	0.86 (0.74 to 0.99)	0.86 (0.74 to 0.99)	0.86 (0.74 to 0.99)	0.85 (0.74 to 0.98)	0.86 (0.74 to 0.99)	0.86 (0.74 to 0.99)	0.86 (0.74 to 0.99)	0.86 (0.74 to 0.99)	0.86 (0.74 to 0.99)
130 to 139	0.92 (0.83 to 1.03)	0.93 (0.84 to 1.03)	0.94 (0.84 to 1.05)	0.91 (0.81 to 1.02)	0.92 (0.82 to 1.02)	0.93 (0.84 to 1.03)	0.93 (0.83 to 1.03)	0.92 (0.83 to 1.03)	0.92 (0.83 to 1.02)	0.92 (0.83 to 1.03)	0.92 (0.83 to 1.03)	0.92 (0.83 to 1.02)	0.92 (0.83 to 1.02)	0.92 (0.83 to 1.02)	0.93 (0.83 to 1.03)	0.93 (0.83 to 1.03)
140 to 149	0.90 (0.82 to 0.99)	0.90 (0.82 to 1.00)	0.89 (0.81 to 0.99)	0.89 (0.81 to 0.99)	0.90 (0.81 to 1.00)	0.90 (0.82 to 1.00)	0.90 (0.82 to 0.99)	0.90 (0.82 to 0.99)	0.91 (0.82 to 1.00)	0.90 (0.82 to 1.00)	0.90 (0.82 to 1.00)	0.90 (0.82 to 0.99)	0.90 (0.82 to 0.99)	0.90 (0.81 to 0.99)	0.90 (0.81 to 0.99)	0.90 (0.82 to 0.99)
150 to 159	0.77 (0.69 to 0.85)	0.78 (0.70 to 0.87)	0.77 (0.69 to 0.87)	0.77 (0.69 to 0.86)	0.77 (0.68 to 0.84)	0.78 (0.70 to 0.87)	0.77 (0.70 to 0.86)	0.77 (0.70 to 0.86)	0.77 (0.70 to 0.86)	0.78 (0.70 to 0.86)	0.78 (0.70 to 0.86)	0.78 (0.68 to 0.86)	0.77 (0.70 to 0.86)	0.77 (0.70 to 0.86)	0.77 (0.70 to 0.86)	0.77 (0.70 to 0.86)
160 to 169	0.86 (0.78 to 0.93)	0.87 (0.79 to 0.94)	0.87 (0.79 to 0.94)	0.87 (0.79 to 0.94)	0.86 (0.79 to 0.94)	0.87 (0.79 to 0.94)	0.86 (0.79 to 0.94)	0.86 (0.79 to 0.94)	0.88 (0.80 to 0.96)	0.86 (0.79 to 0.94)	0.87 (0.80 to 0.96)	0.86 (0.80 to 0.94)	0.86 (0.80 to 0.94)	0.86 (0.79 to 0.94)	0.86 (0.79 to 0.94)	0.86 (0.79 to 0.94)
≥170	0.90 (0.84 to 0.97)	0.90 (0.84 to 0.97)	0.91 (0.85 to 0.97)	0.91 (0.85 to 0.97)	0.90 (0.84 to 0.97)	0.90 (0.84 to 0.97)	0.91 (0.85 to 0.97)	0.90 (0.84 to 0.97)	0.92 (0.86 to 0.99)	0.90 (0.84 to 0.97)	0.90 (0.84 to 0.97)	0.90 (0.85 to 0.97)	0.90 (0.85 to 0.97)	0.91 (0.85 to 0.97)	0.90 (0.84 to 0.96)	0.90 (0.84 to 0.97)
p for interaction	P adjusted=0.70	P adjusted=0.77	P adjusted=0.77	P adjusted=0.63	P adjusted=0.42	P adjusted=0.42	P adjusted=0.70	P adjusted=0.70	P adjusted=0.49	P adjusted=0.84	P adjusted=0.91	P adjusted=0.77	P adjusted=0.70	P adjusted=0.70	P adjusted=0.70	P adjusted=0.77
	P unadjusted=0.10	P unadjusted=0.11	P unadjusted=0.11	P unadjusted=0.09	P unadjusted=0.06	P unadjusted=0.06	P unadjusted=0.10	P unadjusted=0.10	P unadjusted=0.07	P unadjusted=0.12	P unadjusted=0.13	P unadjusted=0.11	P unadjusted=0.10	P unadjusted=0.10	P unadjusted=0.10	P unadjusted=0.11
**Ischaemic heart disease**	**HR 95%CI**	**HR 95%CI**	**HR 95%CI**	**HR 95%CI**	**HR 95%CI**	**HR 95%CI**	**HR 95%CI**	**HR 95%CI**	**HR 95%CI**	**HR 95%CI**	**HR 95%CI**	**HR 95%CI**	**HR 95%CI**	**HR 95%CI**	**HR 95%CI**	**HR 95%CI**
<120	0.84 (0.73 to 0.96)	0.84 (0.73 to 0.96)	0.87 (0.73 to 1.04)	0.83 (0.73 to 0.96)	0.81 (0.70 to 0.93)	0.78 (0.66 to 0.90)	0.83 (0.73 to 0.96)	0.84 (0.73 to 0.96)	0.83 (0.73 to 0.96)	0.83 (0.72 to 0.95)	0.84 (0.73 to 0.96)	0.83 (0.73 to 0.96)	0.83 (0.73 to 0.96)	0.83 (0.73 to 0.96)	0.84 (0.73 to 0.96)	0.83 (0.73 to 0.96)
120 to 129	0.95 (0.85 to 1.05)	0.95 (0.86 to 1.06)	0.94 (0.83 to 1.06)	0.95 (0.85 to 1.05)	0.94 (0.85 to 1.05)	0.93 (0.82 to 1.05)	0.95 (0.86 to 1.05)	0.95 (0.86 to 1.06)	0.95 (0.85 to 1.05)	0.96 (0.87 to 1.06)	0.95 (0.86 to 1.06)	0.95 (0.85 to 1.05)	0.95 (0.86 to 1.05)	0.95 (0.86 to 1.05)	0.96 (0.86 to 1.06)	0.95 (0.86 to 1.05)
130 to 139	0.97 (0.89 to 1.05)	0.96 (0.88 to 1.04)	0.90 (0.82 to 1.00)	0.96 (0.89 to 1.05)	0.97 (0.90 to 1.06)	0.96 (0.87 to 1.06)	0.96 (0.89 to 1.05)	0.96 (0.89 to 1.05)	0.96 (0.89 to 1.05)	0.97 (0.89 to 1.05)	0.96 (0.89 to 1.05)	0.96 (0.89 to 1.05)	0.96 (0.89 to 1.05)	0.96 (0.89 to 1.05)	0.97 (0.89 to 1.05)	0.96 (0.89 to 1.05)
140 to 149	0.94 (0.87 to 1.01)	0.94 (0.87 to 1.01)	0.90 (0.82 to 0.99)	0.94 (0.87 to 1.02)	0.95 (0.88 to 1.03)	0.89 (0.83 to 0.97)	0.95 (0.88 to 1.02)	0.94 (0.87 to 1.02)	0.95 (0.88 to 1.03)	0.95 (0.88 to 1.02)	0.94 (0.87 to 1.01)	0.94 (0.87 to 1.02)	0.94 (0.87 to 1.02)	0.94 (0.87 to 1.02)	0.94 (0.87 to 1.02)	0.94 (0.87 to 1.02)
150 to 159	0.90 (0.83 to 0.98)	0.91 (0.83 to 0.99)	0.91 (0.83 to 1.00)	0.90 (0.83 to 0.98)	0.91 (0.83 to 0.99)	0.91 (0.83 to 1.00)	0.91 (0.84 to 0.99)	0.91 (0.83 to 0.99)	0.91 (0.84 to 0.99)	0.90 (0.83 to 0.98)	0.91 (0.84 to 0.99)	0.91 (0.84 to 0.99)	0.91 (0.84 to 0.99)	0.91 (0.84 to 0.99)	0.91 (0.83 to 0.99)	0.91 (0.84 to 0.99)
160 to 169	0.87 (0.80 to 0.95)	0.87 (0.80 to 0.94)	0.87 (0.80 to 0.94)	0.87 (0.80 to 0.95)	0.87 (0.80 to 0.94)	0.86 (0.78 to 0.94)	0.86 (0.80 to 0.94)	0.86 (0.80 to 0.94)	0.90 (0.83 to 0.98)	0.87 (0.80 to 0.94)	0.87 (0.80 to 0.94)	0.87 (0.80 to 0.94)	0.87 (0.80 to 0.94)	0.87 (0.80 to 0.94)	0.87 (0.80 to 0.94)	0.87 (0.80 to 0.95)
≥170	0.94 (0.88 to 1.01)	0.94 (0.87 to 1.01)	0.95 (0.88 to 1.02)	0.94 (0.87 to 1.00)	0.94 (0.88 to 1.01)	0.92 (0.85 to 0.99)	0.94 (0.87 to 1.01)	0.93 (0.87 to 1.00)	0.93 (0.87 to 1.01)	0.94 (0.87 to 1.01)	0.93 (0.87 to 1.00)	0.93 (0.87 to 1.00)	0.93 (0.87 to 1.00)	0.94 (0.87 to 1.00)	0.94 (0.87 to 1.01)	0.94 (0.87 to 1.00)
p for interaction	P adjusted=1.00	P adjusted=1.00	P adjusted=1.00	P adjusted=1.00	P adjusted=1.00	P adjusted=1.00	P adjusted=1.00	P adjusted=1.00	P adjusted=1.00	P adjusted=1.00	P adjusted=1.00	P adjusted=1.00	P adjusted=1.00	P adjusted=1.00	P adjusted=1.00	P adjusted=1.00
	P unadjusted=0.73	P unadjusted=0.73	P unadjusted=0.85	P unadjusted=0.73	P unadjusted=0.50	P unadjusted=0.34	P unadjusted=0.65	P unadjusted=0.60	P unadjusted=0.78	P unadjusted=0.65	P unadjusted=0.67	P unadjusted=0.70	P unadjusted=0.71	P unadjusted=0.70	P unadjusted=0.69	P unadjusted=0.71
**Heart failure**	**HR 95%CI**	**HR 95%CI**	**HR 95%CI**	**HR 95%CI**	**HR 95%CI**	**HR 95%CI**	**HR 95%CI**	**HR 95%CI**	**HR 95%CI**	**HR 95%CI**	**HR 95%CI**	**HR 95%CI**	**HR 95%CI**	**HR 95%CI**	**HR 95%CI**	**HR 95%CI**
<120	0.79 (0.65 to 0.97)	0.79 (0.65 to 0.97)	0.75 (0.60 to 0.95)	0.77 (0.63 to 0.95)	0.79 (0.65 to 0.99)	0.79 (0.64 to 0.98)	0.78 (0.64 to 0.96)	0.78 (0.64 to 0.96)	0.78 (0.64 to 0.96)	0.79 (0.65 to 0.97)	0.78 (0.64 to 0.96)	0.78 (0.64 to 0.96)	0.78 (0.64 to 0.96)	0.78 (0.64 to 0.96)	0.78 (0.64 to 0.96)	0.78 (0.64 to 0.96)
120 to 129	0.99 (0.85 to 1.16)	0.99 (0.85 to 1.16)	1.00 (0.84 to 1.19)	1.00 (0.85 to 1.16)	0.94 (0.80 to 1.10)	0.99 (0.84 to 1.17)	0.99 (0.85 to 1.15)	0.99 (0.85 to 1.15)	0.99 (0.85 to 1.15)	0.99 (0.85 to 1.16)	0.99 (0.85 to 1.15)	0.99 (0.85 to 1.15)	0.99 (0.85 to 1.15)	0.99 (0.85 to 1.15)	0.99 (0.85 to 1.16)	0.99 (0.85 to 1.15)
130 to 139	0.84 (0.74 to 0.96)	0.84 (0.74 to 0.96)	0.78 (0.68 to 0.91)	0.83 (0.73 to 0.95)	0.84 (0.74 to 0.96)	0.90 (0.78 to 1.04)	0.86 (0.74 to 0.96)	0.85 (0.74 to 0.96)	0.85 (0.74 to 0.96)	0.84 (0.74 to 0.96)	0.85 (0.74 to 0.96)	0.85 (0.74 to 0.96)	0.85 (0.74 to 0.96)	0.85 (0.74 to 0.96)	0.85 (0.74 to 0.96)	0.85 (0.74 to 0.96)
140 to 149	0.93 (0.83 to 1.05)	0.96 (0.86 to 1.08)	0.92 (0.80 to 1.05)	0.96 (0.85 to 1.08)	0.95 (0.85 to 1.08)	0.99 (0.87 to 1.12)	0.95 (0.85 to 1.07)	0.95 (0.85 to 1.07)	0.95 (0.85 to 1.07)	0.95 (0.85 to 1.07)	0.95 (0.85 to 1.07)	0.95 (0.85 to 1.07)	0.95 (0.85 to 1.07)	0.95 (0.85 to 1.07)	0.95 (0.85 to 1.07)	0.95 (0.85 to 1.07)
150 to 159	0.87 (0.76 to 0.99)	0.87 (0.76 to 0.99)	0.88 (0.76 to 1.02)	0.89 (0.78 to 1.01)	0.90 (0.79 to 1.02)	0.92 (0.80 to 1.06)	0.88 (0.77 to 1.00)	0.88 (0.78 to 1.00)	0.89 (0.78 to 1.01)	0.89 (0.78 to 1.00)	0.88 (0.78 to 1.00)	0.88 (0.78 to 1.00)	0.88 (0.78 to 1.00)	0.88 (0.78 to 1.00)	0.88 (0.78 to 1.00)	0.88 (0.78 to 1.00)
160 to 169	0.70 (0.61 to 0.81)	0.69 (0.60 to 0.79)	0.70 (0.61 to 0.81)	0.71 (0.61 to 0.82)	0.67 (0.58 to 0.78)	0.76 (0.64 to 0.87)	0.70 (0.61 to 0.81)	0.69 (0.60 to 0.80)	0.70 (0.61 to 0.82)	0.69 (0.60 to 0.80)	0.70 (0.61 to 0.80)	0.70 (0.61 to 0.80)	0.70 (0.60 to 0.80)	0.70 (0.61 to 0.80)	0.70 (0.60 to 0.80)	0.70 (0.60 to 0.80)
≥170	0.88 (0.78 to 0.99)	0.89 (0.79 to 1.00)	0.89 (0.79 to 1.00)	0.89 (0.79 to 1.00)	0.88 (0.78 to 0.99)	0.91 (0.80 to 1.03)	0.89 (0.79 to 1.00)	0.89 (0.79 to 1.00)	0.90 (0.79 to 1.02)	0.89 (0.79 to 1.00)	0.90 (0.79 to 1.00)	0.90 (0.79 to 1.00)	0.89 (0.79 to 1.00)	0.90 (0.80 to 1.01)	0.88 (0.79 to 1.00)	0.89 (0.80 to 1.00)
p for interaction	P adjusted=0.56	P adjusted=0.35	P adjusted=0.49	P adjusted=0.49	P adjusted=0.56	P adjusted=1.00	P adjusted=0.49	P adjusted=0.28	P adjusted=0.77	P adjusted=0.35	P adjusted=0.42	P adjusted=0.42	P adjusted=0.35	P adjusted=0.42	P adjusted=0.27	P adjusted=0.35
	P unadjusted=0.08	P unadjusted=0.05	P unadjusted=0.07	P unadjusted=0.07	P unadjusted=0.08	P unadjusted=0.33	P unadjusted=0.07	P unadjusted=0.04	P unadjusted=0.11	P unadjusted=0.05	P unadjusted=0.06	P unadjusted=0.06	P unadjusted=0.05	P unadjusted=0.06	P unadjusted=0.03	P unadjusted=0.05
**Cardiovascular death**	**HR 95%CI**	**HR 95%CI**	**HR 95%CI**	**HR 95%CI**	**HR 95%CI**	**HR 95%CI**	**HR 95%CI**	**HR 95%CI**	**HR 95%CI**	**HR 95%CI**	**HR 95%CI**	**HR 95%CI**	**HR 95%CI**	**HR 95%CI**	**HR 95%CI**	**HR 95%CI**
<120	0.90 (0.71 to 1.13)	0.90 (0.72 to 1.13)	0.86 (0.66 to 1.12)	0.87 (0.69 to 1.09)	0.85 (0.66 to 1.08)	0.87 (0.69 to 1.10)	0.90 (0.72 to 1.12)	0.90 (0.72 to 1.12)	0.90 (0.72 to 1.12)	0.90 (0.72 to 1.12)	0.90 (0.72 to 1.12)	0.90 (0.72 to 1.12)	0.90 (0.72 to 1.12)	0.90 (0.72 to 1.12)	0.90 (0.72 to 1.12)	0.90 (0.72 to 1.12)
120 to 129	0.89 (0.75 to 1.05)	0.88 (0.75 to 1.04)	0.89 (0.74 to 1.07)	0.91 (0.77 to 1.08)	0.88 (0.74 to 1.06)	0.90 (0.75 to 1.07)	0.88 (0.75 to 1.04)	0.89 (0.75 to 1.04)	0.89 (0.75 to 1.05)	0.89 (0.75 to 1.05)	0.88 (0.75 to 1.04)	0.88 (0.75 to 1.04)	0.89 (0.75 to 1.04)	0.89 (0.75 to 1.04)	0.89 (0.75 to 1.04)	0.89 (0.75 to 1.04)
130 to 139	0.97 (0.86 to 1.10)	0.98 (0.87 to 1.11)	0.96 (0.84 to 1.10)	0.96 (0.85 to 1.09)	1.02 (0.90 to 1.16)	1.04 (0.90 to 1.19)	0.99 (0.87 to 1.11)	0.98 (0.87 to 1.11)	0.97 (0.86 to 1.10)	0.98 (0.87 to 1.11)	0.98 (0.87 to 1.11)	0.98 (0.87 to 1.11)	0.98 (0.87 to 1.11)	0.98 (0.87 to 1.11)	0.98 (0.87 to 1.11)	0.98 (0.87 to 1.11)
140 to 149	0.97 (0.86 to 1.09)	0.98 (0.87 to 1.10)	0.92 (0.80 to 1.06)	0.98 (0.87 to 1.03)	1.02 (0.90 to 1.15)	0.93 (0.82 to 1.06)	0.98 (0.87 to 1.10)	0.97 (0.86 to 1.10)	0.99 (0.87 to 1.11)	0.97 (0.86 to 1.09)	0.97 (0.87 to 1.10)	0.97 (0.87 to 1.10)	0.97 (0.87 to 1.10)	0.97 (0.87 to 1.10)	0.97 (0.87 to 1.10)	0.97 (0.87 to 1.10)
150 to 159	1.00 (0.88 to 1.14)	1.01 (0.89 to 1.14)	1.01 (0.89 to 1.14)	0.99 (0.87 to 1.12)	0.99 (0.88 to 1.13)	1.01 (0.88 to 1.16)	1.00 (0.88 to 1.13)	1.00 (0.89 to 1.14)	1.01 (0.89 to 1.15)	1.00 (0.88 to 1.13)	0.99 (0.88 to 1.13)	1.00 (0.88 to 1.13)	1.00 (0.88 to 1.13)	1.00 (0.88 to 1.13)	1.00 (0.88 to 1.13)	1.00 (0.88 to 1.14)
160 to 169	0.90 (0.81 to 0.99)	0.90 (0.81 to 0.99)	0.99 (0.86 to 1.13)	0.89 (0.80 to 0.98)	0.90 (0.82 to 1.00)	0.90 (0.80 to 1.00)	0.90 (0.82 to 1.00)	0.90 (0.81 to 0.99)	0.93 (0.83 to 1.03)	0.90 (0.82 to 0.99)	0.90 (0.81 to 0.99)	0.90 (0.82 to 0.99)	0.90 (0.81 to 0.99)	0.90 (0.82 to 0.99)	0.90 (0.81 to 0.99)	0.90 (0.82 to 0.99)
≥170	0.95 (0.88 to 1.02)	0.95 (0.88 to 1.02)	0.90 (0.82 to 1.00)	0.94 (0.87 to 1.01)	0.95 (0.88 to 1.02)	0.92 (0.85 to 1.00)	0.95 (0.88 to 1.02)	0.95 (0.88 to 1.02)	0.97 (0.90 to 1.05)	0.95 (0.88 to 1.01)	0.94 (0.87 to 1.01)	0.94 (0.88 to 1.02)	0.95 (0.88 to 1.02)	0.95 (0.88 to 1.02)	0.94 (0.87 to 1.01)	0.94 (0.88 to 1.02)
p for interaction	P adjusted=0.49	P adjusted=0.42	P adjusted=0.42	P adjusted=1.00	P adjusted=0.27	P adjusted=1.00	P adjusted=0.49	P adjusted=0.42	P adjusted=0.77	P adjusted=0.56	P adjusted=0.42	P adjusted=0.49	P adjusted=0.42	P adjusted=0.49	P adjusted=0.42	P adjusted=0.49
	P unadjusted=0.07	P unadjusted=0.06	P unadjusted=0.06	P unadjusted=0.19	P unadjusted=0.03	P unadjusted=0.15	P unadjusted=0.07	P unadjusted=0.06	P unadjusted=0.11	P unadjusted=0.08	P unadjusted=0.06	P unadjusted=0.07	P unadjusted=0.06	P unadjusted=0.07	P unadjusted=0.06	P unadjusted=0.07
**All-cause death**	**HR 95%CI**	**HR 95%CI**	**HR 95%CI**	**HR 95%CI**	**HR 95%CI**	**HR 95%CI**	**HR 95%CI**	**HR 95%CI**	**HR 95%CI**	**HR 95%CI**	**HR 95%CI**	**HR 95%CI**	**HR 95%CI**	**HR 95%CI**	**HR 95%CI**	**HR 95%CI**
<120	0.90 (0.80 to 1.03)	0.88 (0.78 to 1.00)	0.88 (0.76 to 1.00)	0.87 (0.77 to 0.99)	0.87 (0.77 to 0.99)	0.86 (0.75 to 0.97)	0.88 (0.78 to 1.00)	0.88 (0.78 to 1.00)	0.88 (0.78 to 1.00)	0.88 (0.78 to 1.00)	0.88 (0.78 to 1.00)	0.88 (0.78 to 1.00)	0.88 (0.78 to 1.00)	0.88 (0.78 to 1.00)	0.88 (0.78 to 1.00)	0.88 (0.78 to 1.00)
120 to 129	1.00 (0.91 to 1.10)	1.00 (0.90 to 1.10)	0.99 (0.89 to 1.10)	1.02 (0.92 to 1.12)	0.99 (0.90 to 1.10)	0.99 (0.90 to 1.09)	1.00 (0.91 to 1.10)	1.00 (0.91 to 1.10)	1.00 (0.91 to 1.10)	1.00 (0.90 to 1.10)	1.00 (0.90 to 1.10)	1.00 (0.90 to 1.10)	1.00 (0.90 to 1.10)	1.00 (0.91 to 1.10)	1.00 (0.91 to 1.10)	1.00 (0.91 to 1.10)
130 to 139	0.97 (0.90 to 1.05)	0.98 (0.91 to 1.06)	0.95 (0.88 to 1.03)	0.97 (0.90 to 1.05)	0.99 (0.92 to 1.07)	0.97 (0.89 to 1.06)	0.98 (0.91 to 1.06)	0.98 (0.91 to 1.06)	0.98 (0.91 to 1.05)	0.98 (0.91 to 1.06)	0.98 (0.91 to 1.06)	0.98 (0.91 to 1.06)	0.98 (0.91 to 1.06)	0.98 (0.91 to 1.06)	0.98 (0.91 to 1.06)	0.98 (0.91 to 1.06)
140 to 149	1.00 (0.93 to 1.07)	1.00 (0.93 to 1.07)	0.97 (0.90 to 1.05)	0.99 (0.92 to 1.06)	1.01 (0.94 to 1.09)	0.97 (0.90 to 1.04)	0.99 (0.92 to 1.06)	0.99 (0.92 to 1.06)	1.00 (0.93 to 1.07)	0.99 (0.92 to 1.06)	0.99 (0.92 to 1.06)	0.99 (0.92 to 1.06)	0.99 (0.92 to 1.06)	0.99 (0.92 to 1.06)	0.99 (0.92 to 1.07)	0.99 (0.92 to 1.06)
150 to 159	0.96 (0.89 to 1.03)	0.95 (0.88 to 1.03)	0.94 (0.87 to 1.02)	0.95 (0.88 to 1.02)	0.96 (0.88 to 1.03)	0.94 (0.87 to 1.01)	0.95 (0.88 to 1.03)	0.95 (0.89 to 1.03)	0.96 (0.88 to 1.03)	0.95 (0.88 to 1.03)	0.95 (0.88 to 1.03)	0.95 (0.88 to 1.03)	0.95 (0.88 to 1.03)	0.95 (0.88 to 1.03)	0.95 (0.88 to 1.03)	0.95 (0.88 to 1.03)
160 to 169	0.94 (0.88 to 1.00)	0.94 (0.88 to 1.00)	0.94 (0.88 to 1.00)	0.94 (0.88 to 1.00)	0.94 (0.88 to 1.00)	0.94 (0.88 to 1.01)	0.94 (0.88 to 1.00)	0.94 (0.88 to 1.00)	0.95 (0.89 to 1.00)	0.94 (0.88 to 1.00)	0.94 (0.88 to 1.00)	0.94 (0.88 to 1.00)	0.94 (0.88 to 1.01)	0.94 (0.88 to 1.00)	0.94 (0.88 to 1.00)	0.94 (0.88 to 1.00)
≥170	0.97 (0.93 to 1.02)	0.97 (0.93 to 1.02)	0.97 (0.93 to 1.02)	0.97 (0.92 to 1.02)	0.98 (0.93 to 1.03)	0.96 (0.91 to 1.01)	0.97 (0.93 to 1.02)	0.97 (0.92 to 1.02)	0.99 (0.94 to 1.04)	0.97 (0.93 to 1.02)	0.97 (0.92 to 1.02)	0.97 (0.93 to 1.02)	0.97 (0.93 to 1.02)	0.97 (0.93 to 1.02)	0.97 (0.92 to 1.01)	0.97 (0.93 to 1.02)
p for interaction	P adjusted=1.00	P adjusted=1.00	P adjusted=1.00	P adjusted=1.00	P adjusted=1.00	P adjusted=1.00	P adjusted=1.00	P adjusted=1.00	P adjusted=1.00	P adjusted=1.00	P adjusted=1.00	P adjusted=1.00	P adjusted=1.00	P adjusted=1.00	P adjusted=1.00	P adjusted=1.00
	P unadjusted=0.30	P unadjusted=0.27	P unadjusted=0.29	P unadjusted=0.32	P unadjusted=0.15	P unadjusted=0.84	P unadjusted=0.31	P unadjusted=0.23	P unadjusted=0.31	P unadjusted=0.33	P unadjusted=0.34	P unadjusted=0.31	P unadjusted=0.28	P unadjusted=0.42	P unadjusted=0.19	P unadjusted=0.35

Table S4: Continues

	Trial excluded from analysis															
Baseline systolic blood pressure	**DIABHYCAR**	**Dutch TIA**	**ELSA**	**EUROPA**	**EWPHE**	**HIJ-CREATE**	**HOMED-BP**	**HOPE**	**HYVET**	**INSIGHT**	**INVEST**	**JMIC-B**	**LIFE**	**MOSES**	**NICS-EH**	**NORDIL**
**Major cardiovascular events**	**HR 95%CI**	**HR 95%CI**	**HR 95%CI**	**HR 95%CI**	**HR 95%CI**	**HR 95%CI**	**HR 95%CI**	**HR 95%CI**	**HR 95%CI**	**HR 95%CI**	**HR 95%CI**	**HR 95%CI**	**HR 95%CI**	**HR 95%CI**	**HR 95%CI**	**HR 95%CI**
<120	0.79 (0.72 to 0.87)	0.79 (0.72 to 0.87)	0.79 (0.72 to 0.87)	0.80 (0.72 to 0.88)	0.79 (0.72 to 0.87)	0.79 (0.72 to 0.87)	0.79 (0.72 to 0.87)	0.79 (0.72 to 0.87)	0.79 (0.72 to 0.87)	0.79 (0.72 to 0.87)	0.79 (0.72 to 0.87)	0.79 (0.72 to 0.87)	0.79 (0.72 to 0.87)	0.79 (0.72 to 0.87)	0.79 (0.72 to 0.87)	0.79 (0.72 to 0.87)
120 to 129	0.91 (0.85 to 0.98)	0.91 (0.85 to 0.98)	0.91 (0.85 to 0.98)	0.92 (0.85 to 0.99)	0.91 (0.85 to 0.98)	0.91 (0.85 to 0.98)	0.91 (0.85 to 0.98)	0.92 (0.86 to 0.99)	0.91 (0.85 to 0.98)	0.91 (0.85 to 0.98)	0.91 (0.84 to 0.98)	0.91 (0.85 to 0.98)	0.91 (0.85 to 0.98)	0.91 (0.85 to 0.98)	0.91 (0.85 to 0.98)	0.91 (0.85 to 0.98)
130 to 139	0.94 (0.89 to 0.99)	0.94 (0.89 to 0.99)	0.94 (0.89 to 0.99)	0.95 (0.90 to 1.01)	0.94 (0.89 to 0.99)	0.94 (0.89 to 0.99)	0.94 (0.89 to 0.99)	0.95 (0.89 to 1.00)	0.94 (0.89 to 0.99)	0.94 (0.89 to 0.99)	0.94 (0.89 to 1.00)	0.94 (0.89 to 0.99)	0.94 (0.89 to 0.99)	0.94 (0.89 to 0.99)	0.94 (0.89 to 0.99)	0.94 (0.89 to 0.99)
140 to 149	0.93 (0.88 to 0.98)	0.93 (0.88 to 0.98)	0.93 (0.88 to 0.98)	0.94 (0.89 to 0.99)	0.93 (0.88 to 0.98)	0.93 (0.88 to 0.98)	0.93 (0.88 to 0.98)	0.94 (0.89 to 0.99)	0.93 (0.88 to 0.98)	0.93 (0.88 to 0.98)	0.93 (0.88 to 0.98)	0.93 (0.88 to 0.98)	0.93 (0.88 to 0.98)	0.93 (0.88 to 0.98)	0.93 (0.88 to 0.98)	0.93 (0.88 to 0.98)
150 to 159	0.88 (0.83 to 0.94)	0.88 (0.83 to 0.94)	0.88 (0.83 to 0.94)	0.89 (0.84 to 0.94)	0.88 (0.83 to 0.94)	0.88 (0.83 to 0.94)	0.88 (0.83 to 0.94)	0.89 (0.84 to 0.94)	0.88 (0.83 to 0.94)	0.88 (0.83 to 0.94)	0.88 (0.83 to 0.94)	0.88 (0.83 to 0.94)	0.88 (0.83 to 0.94)	0.88 (0.83 to 0.94)	0.88 (0.83 to 0.94)	0.88 (0.83 to 0.94)
160 to 169	0.87 (0.82 to 0.92)	0.87 (0.82 to 0.92)	0.87 (0.83 to 0.92)	0.87 (0.82 to 0.92)	0.87 (0.83 to 0.92)	0.87 (0.83 to 0.92)	0.87 (0.83 to 0.92)	0.87 (0.83 to 0.92)	0.88 (0.83 to 0.93)	0.87 (0.83 to 0.92)	0.88 (0.83 to 0.92)	0.87 (0.83 to 0.92)	0.87 (0.82 to 0.92)	0.87 (0.82 to 0.92)	0.87 (0.82 to 0.92)	0.87 (0.82 to 0.92)
≥170	0.90 (0.86 to 0.94)	0.90 (0.86 to 0.94)	0.90 (0.86 to 0.94)	0.90 (0.87 to 0.94)	0.90 (0.86 to 0.94)	0.90 (0.86 to 0.94)	0.90 (0.86 to 0.94)	0.91 (0.87 to 0.95)	0.91 (0.87 to 0.95)	0.90 (0.86 to 0.94)	0.90 (0.86 to 0.94)	0.90 (0.86 to 0.94)	0.91 (0.87 to 0.95)	0.90 (0.86 to 0.94)	0.90 (0.86 to 0.94)	0.90 (0.86 to 0.94)
p for interaction	P adjusted=0.35	P adjusted=0.28	P adjusted=0.35	P adjusted=0.28	P adjusted=0.35	P adjusted=0.42	P adjusted=0.35	P adjusted=0.28	P adjusted=0.42	P adjusted=0.42	P adjusted=0.56	P adjusted=0.35	P adjusted=0.28	P adjusted=0.28	P adjusted=0.28	P adjusted=0.28
	P unadjusted=0.05	P unadjusted=0.04	P unadjusted=0.05	P unadjusted=0.04	P unadjusted=0.05	P unadjusted=0.06	P unadjusted=0.05	P unadjusted=0.04	P unadjusted=0.06	P unadjusted=0.06	P unadjusted=0.08	P unadjusted=0.05	P unadjusted=0.04	P unadjusted=0.04	P unadjusted=0.04	P unadjusted=0.04
**Stroke**	**HR 95%CI**	**HR 95%CI**	**HR 95%CI**	**HR 95%CI**	**HR 95%CI**	**HR 95%CI**	**HR 95%CI**	**HR 95%CI**	**HR 95%CI**	**HR 95%CI**	**HR 95%CI**	**HR 95%CI**	**HR 95%CI**	**HR 95%CI**	**HR 95%CI**	**HR 95%CI**
<120	0.79 (0.65 to 0.97)	0.79 (0.65 to 0.97)	0.79 (0.65 to 0.97)	0.80 (0.65 to 0.98)	0.79 (0.65 to 0.97)	0.79 (0.65 to 0.97)	0.79 (0.65 to 0.97)	0.83 (0.68 to 1.02)	0.79 (0.65 to 0.97)	0.79 (0.65 to 0.97)	0.80 (0.65 to 0.97)	0.79 (0.65 to 0.97)	0.79 (0.65 to 0.97)	0.79 (0.65 to 0.97)	0.79 (0.65 to 0.97)	0.79 (0.65 to 0.97)
120 to 129	0.86 (0.75 to 0.99)	0.86 (0.75 to 1.00)	0.86 (0.74 to 0.99)	0.86 (0.74 to 0.99)	0.86 (0.74 to 0.99)	0.86 (0.74 to 0.99)	0.86 (0.74 to 0.99)	0.86 (0.75 to 1.00)	0.86 (0.74 to 0.99)	0.86 (0.74 to 0.99)	0.85 (0.74 to 0.98)	0.85 (0.74 to 0.98)	0.86 (0.74 to 0.99)	0.85 (0.74 to 0.98)	0.85 (0.74 to 0.99)	0.86 (0.74 to 0.99)
130 to 139	0.92 (0.83 to 1.03)	0.93 (0.83 to 1.03)	0.92 (0.83 to 1.03)	0.92 (0.83 to 1.03)	0.92 (0.83 to 1.03)	0.93 (0.83 to 1.03)	0.93 (0.83 to 1.03)	0.94 (0.84 to 1.04)	0.92 (0.83 to 1.03)	0.92 (0.83 to 1.03)	0.93 (0.84 to 1.03)	0.93 (0.83 to 1.03)	0.93 (0.83 to 1.03)	0.93 (0.83 to 1.03)	0.92 (0.83 to 1.03)	0.93 (0.83 to 1.03)
140 to 149	0.90 (0.81 to 0.99)	0.90 (0.82 to 0.99)	0.90 (0.82 to 0.99)	0.90 (0.81 to 0.99)	0.90 (0.82 to 0.99)	0.90 (0.81 to 0.99)	0.90 (0.81 to 0.99)	0.91 (0.82 to 1.00)	0.90 (0.82 to 0.99)	0.90 (0.82 to 0.99)	0.90 (0.82 to 0.99)	0.90 (0.81 to 0.99)	0.90 (0.82 to 0.99)	0.90 (0.81 to 0.99)	0.90 (0.82 to 0.99)	0.90 (0.82 to 0.99)
150 to 159	0.77 (0.70 to 0.86)	0.77 (0.70 to 0.86)	0.77 (0.70 to 0.86)	0.77 (0.70 to 0.86)	0.77 (0.70 to 0.86)	0.77 (0.70 to 0.86)	0.77 (0.70 to 0.86)	0.77 (0.70 to 0.86)	0.77 (0.70 to 0.86)	0.77 (0.70 to 0.86)	0.77 (0.70 to 0.86)	0.77 (0.70 to 0.86)	0.77 (0.70 to 0.86)	0.77 (0.70 to 0.86)	0.77 (0.70 to 0.86)	0.77 (0.70 to 0.86)
160 to 169	0.86 (0.79 to 0.94)	0.86 (0.79 to 0.94)	0.86 (0.79 to 0.94)	0.86 (0.79 to 0.94)	0.86 (0.79 to 0.94)	0.86 (0.79 to 0.94)	0.86 (0.79 to 0.94)	0.86 (0.79 to 0.94)	0.86 (0.79 to 0.94)	0.87 (0.80 to 0.95)	0.87 (0.80 to 0.96)	0.86 (0.79 to 0.93)	0.86 (0.79 to 0.94)	0.86 (0.79 to 0.94)	0.86 (0.79 to 0.94)	0.86 (0.79 to 0.94)
≥170	0.90 (0.84 to 0.97)	0.91 (0.84 to 0.97)	0.91 (0.85 to 0.97)	0.90 (0.84 to 0.97)	0.90 (0.84 to 0.97)	0.90 (0.84 to 0.97)	0.90 (0.84 to 0.97)	0.90 (0.84 to 0.97)	0.92 (0.85 to 0.98)	0.90 (0.84 to 0.97)	0.90 (0.84 to 0.97)	0.90 (0.84 to 0.97)	0.93 (0.87 to 1.00)	0.90 (0.84 to 0.97)	0.90 (0.84 to 0.97)	0.88 (0.82 to 0.94)
p for interaction	P adjusted=0.77	P adjusted=0.70	P adjusted=0.77	P adjusted=0.91	P adjusted=0.70	P adjusted=0.98	P adjusted=0.70	P adjusted=0.77	P adjusted=0.63	P adjusted=0.63	P adjusted=0.63	P adjusted=0.77	P adjusted=0.35	P adjusted=0.77	P adjusted=0.77	P adjusted=1.00
	P unadjusted=0.11	P unadjusted=0.10	P unadjusted=0.11	P unadjusted=0.13	P unadjusted=0.10	P unadjusted=0.14	P unadjusted=0.10	P unadjusted=0.11	P unadjusted=0.09	P unadjusted=0.09	P unadjusted=0.09	P unadjusted=0.11	P unadjusted=0.05	P unadjusted=0.11	P unadjusted=0.11	P unadjusted=0.15
**Ischaemic heart disease**	**HR 95%CI**	**HR 95%CI**	**HR 95%CI**	**HR 95%CI**	**HR 95%CI**	**HR 95%CI**	**HR 95%CI**	**HR 95%CI**	**HR 95%CI**	**HR 95%CI**	**HR 95%CI**	**HR 95%CI**	**HR 95%CI**	**HR 95%CI**	**HR 95%CI**	**HR 95%CI**
<120	0.83 (0.73 to 0.96)	0.83 (0.73 to 0.96)	0.83 (0.73 to 0.96)	0.85 (0.74 to 0.98)	0.83 (0.73 to 0.96)	0.83 (0.73 to 0.96)	0.83 (0.73 to 0.96)	0.83 (0.72 to 0.96)	0.83 (0.73 to 0.96)	0.83 (0.73 to 0.96)	0.84 (0.73 to 0.96)	0.83 (0.73 to 0.96)	0.83 (0.73 to 0.96)	0.83 (0.73 to 0.96)	0.83 (0.73 to 0.96)	0.83 (0.73 to 0.96)
120 to 129	0.95 (0.86 to 1.05)	0.95 (0.86 to 1.05)	0.95 (0.86 to 1.05)	0.97 (0.87 to 1.07)	0.95 (0.86 to 1.05)	0.95 (0.86 to 1.05)	0.95 (0.86 to 1.05)	0.97 (0.87 to 1.08)	0.95 (0.86 to 1.05)	0.95 (0.86 to 1.05)	0.95 (0.86 to 1.05)	0.95 (0.86 to 1.05)	0.95 (0.86 to 1.05)	0.95 (0.86 to 1.05)	0.95 (0.86 to 1.05)	0.95 (0.86 to 1.05)
130 to 139	0.96 (0.89 to 1.05)	0.96 (0.89 to 1.05)	0.97 (0.89 to 1.05)	0.99 (0.91 to 1.07)	0.96 (0.89 to 1.05)	0.96 (0.89 to 1.05)	0.96 (0.89 to 1.05)	0.97 (0.89 to 1.07)	0.96 (0.89 to 1.05)	0.96 (0.89 to 1.05)	0.96 (0.88 to 1.04)	0.96 (0.89 to 1.05)	0.97 (0.89 to 1.05)	0.97 (0.89 to 1.05)	0.96 (0.89 to 1.05)	0.97 (0.89 to 1.05)
140 to 149	0.94 (0.87 to 1.02)	0.94 (0.87 to 1.02)	0.94 (0.87 to 1.02)	0.96 (0.87 to 1.02)	0.94 (0.87 to 1.02)	0.94 (0.87 to 1.02)	0.94 (0.87 to 1.02)	0.95 (0.88 to 1.03)	0.94 (0.87 to 1.02)	0.94 (0.87 to 1.02)	0.94 (0.87 to 1.01)	0.94 (0.87 to 1.02)	0.94 (0.87 to 1.02)	0.94 (0.87 to 1.02)	0.94 (0.87 to 1.02)	0.94 (0.87 to 1.02)
150 to 159	0.91 (0.83 to 0.99)	0.90 (0.83 to 0.99)	0.91 (0.83 to 0.99)	0.91 (0.84 to 1.00)	0.90 (0.83 to 0.99)	0.91 (0.83 to 0.99)	0.91 (0.83 to 0.99)	0.91 (0.83 to 0.99)	0.91 (0.83 to 0.99)	0.91 (0.83 to 0.99)	0.91 (0.83 to 0.99)	0.91 (0.83 to 0.99)	0.90 (0.83 to 0.98)	0.91 (0.83 to 0.99)	0.91 (0.83 to 0.99)	0.90 (0.83 to 0.98)
160 to 169	0.87 (0.80 to 0.94)	0.87 (0.80 to 0.94)	0.87 (0.80 to 0.94)	0.87 (0.80 to 0.95)	0.87 (0.80 to 0.95)	0.87 (0.80 to 0.95)	0.87 (0.80 to 0.95)	0.87 (0.80 to 0.95)	0.87 (0.80 to 0.95)	0.87 (0.80 to 0.95)	0.88 (0.81 to 0.95)	0.87 (0.80 to 0.94)	0.87 (0.80 to 0.94)	0.87 (0.80 to 0.95)	0.87 (0.80 to 0.95)	0.87 (0.80 to 0.95)
≥170	0.94 (0.87 to 1.00)	0.94 (0.87 to 1.00)	0.93 (0.87 to 1.00)	0.94 (0.87 to 1.00)	0.93 (0.86 to 1.00)	0.94 (0.87 to 1.00)	0.94 (0.87 to 1.00)	0.95 (0.89 to 1.02)	0.94 (0.87 to 1.00)	0.94 (0.87 to 1.00)	0.94 (0.87 to 1.00)	0.94 (0.87 to 1.00)	0.93 (0.87 to 1.00)	0.93 (0.87 to 1.00)	0.94 (0.87 to 1.00)	0.95 (0.87 to 1.01)
p for interaction	P adjusted=1.00	P adjusted=1.00	P adjusted=1.00	P adjusted=1.00	P adjusted=1.00	P adjusted=1.00	P adjusted=1.00	P adjusted=1.00	P adjusted=1.00	P adjusted=1.00	P adjusted=1.00	P adjusted=1.00	P adjusted=1.00	P adjusted=1.00	P adjusted=1.00	P adjusted=1.00
	P unadjusted=0.70	P unadjusted=0.79	P unadjusted=0.70	P unadjusted=0.68	P unadjusted=0.72	P unadjusted=0.71	P unadjusted=0.72	P unadjusted=0.61	P unadjusted=0.72	P unadjusted=0.74	P unadjusted=0.87	P unadjusted=0.70	P unadjusted=0.69	P unadjusted=0.70	P unadjusted=0.72	P unadjusted=0.73
**Heart failure**	**HR 95%CI**	**HR 95%CI**	**HR 95%CI**	**HR 95%CI**	**HR 95%CI**	**HR 95%CI**	**HR 95%CI**	**HR 95%CI**	**HR 95%CI**	**HR 95%CI**	**HR 95%CI**	**HR 95%CI**	**HR 95%CI**	**HR 95%CI**	**HR 95%CI**	**HR 95%CI**
<120	0.78 (0.64 to 0.96)	0.78 (0.64 to 0.96)	0.78 (0.64 to 0.96)	0.79 (0.65 to 0.97)	0.78 (0.64 to 0.96)	0.78 (0.64 to 0.95)	0.78 (0.64 to 0.96)	0.78 (0.64 to 0.96)	0.78 (0.64 to 0.96)	0.78 (0.64 to 0.96)	0.78 (0.64 to 0.96)	0.78 (0.64 to 0.96)	0.78 (0.64 to 0.96)	0.78 (0.64 to 0.96)	0.78 (0.64 to 0.96)	0.78 (0.64 to 0.96)
120 to 129	0.99 (0.85 to 1.15)	0.99 (0.85 to 1.15)	0.99 (0.85 to 1.15)	1.01 (0.87 to 1.18)	0.99 (0.85 to 1.15)	0.99 (0.85 to 1.15)	0.99 (0.85 to 1.15)	0.99 (0.85 to 1.15)	0.99 (0.85 to 1.15)	0.99 (0.85 to 1.15)	0.99 (0.85 to 1.15)	0.99 (0.85 to 1.16)	0.99 (0.85 to 1.16)	0.99 (0.85 to 1.16)	0.99 (0.85 to 1.16)	0.99 (0.85 to 1.15)
130 to 139	0.85 (0.75 to 0.97)	0.85 (0.75 to 0.97)	0.85 (0.74 to 0.96)	0.87 (0.76 to 0.99)	0.85 (0.74 to 0.96)	0.84 (0.74 to 0.96)	0.85 (0.74 to 0.96)	0.85 (0.74 to 0.96)	0.85 (0.74 to 0.96)	0.85 (0.74 to 0.96)	0.85 (0.74 to 0.96)	0.85 (0.74 to 0.96)	0.85 (0.74 to 0.96)	0.84 (0.74 to 0.96)	0.85 (0.74 to 0.96)	0.85 (0.74 to 0.96)
140 to 149	0.95 (0.84 to 1.07)	0.95 (0.84 to 1.07)	0.95 (0.85 to 1.07)	0.97 (0.86 to 1.09)	0.95 (0.85 to 1.07)	0.95 (0.85 to 1.07)	0.95 (0.85 to 1.07)	0.95 (0.85 to 1.07)	0.95 (0.85 to 1.07)	0.95 (0.85 to 1.07)	0.95 (0.85 to 1.07)	0.95 (0.85 to 1.07)	0.95 (0.85 to 1.07)	0.95 (0.85 to 1.07)	0.95 (0.85 to 1.07)	0.95 (0.85 to 1.07)
150 to 159	0.89 (0.78 to 1.00)	0.88 (0.78 to 1.00)	0.89 (0.78 to 1.00)	0.89 (0.78 to 1.00)	0.89 (0.78 to 1.00)	0.89 (0.78 to 1.00)	0.88 (0.78 to 1.00)	0.88 (0.78 to 1.00)	0.88 (0.78 to 1.00)	0.88 (0.78 to 1.00)	0.88 (0.78 to 1.00)	0.88 (0.78 to 1.00)	0.88 (0.78 to 1.00)	0.88 (0.77 to 0.99)	0.88 (0.78 to 1.00)	0.88 (0.78 to 1.00)
160 to 169	0.70 (0.61 to 0.80)	0.70 (0.61 to 0.80)	0.70 (0.61 to 0.80)	0.70 (0.60 to 0.80)	0.69 (0.60 to 0.80)	0.70 (0.61 to 0.81)	0.70 (0.61 to 0.81)	0.70 (0.61 to 0.80)	0.75 (0.65 to 0.88)	0.70 (0.61 to 0.80)	0.70 (0.61 to 0.80)	0.70 (0.61 to 0.80)	0.70 (0.61 to 0.81)	0.70 (0.60 to 0.80)	0.70 (0.60 to 0.80)	0.70 (0.61 to 0.80)
≥170	0.90 (0.80 to 1.01)	0.90 (0.80 to 1.00)	0.90 (0.80 to 1.00)	0.90 (0.80 to 1.01)	0.87 (0.76 to 0.99)	0.90 (0.80 to 1.00)	0.90 (0.80 to 1.00)	0.90 (0.80 to 1.00)	0.92 (0.81 to 1.04)	0.90 (0.80 to 1.00)	0.90 (0.80 to 1.01)	0.90 (0.80 to 1.00)	0.89 (0.80 to 1.00)	0.89 (0.79 to 1.00)	0.89 (0.79 to 1.00)	0.89 (0.79 to 1.00)
p for interaction	P adjusted=0.49	P adjusted=0.42	P adjusted=0.42	P adjusted=0.28	P adjusted=0.42	P adjusted=0.42	P adjusted=0.49	P adjusted=0.42	P adjusted=0.91	P adjusted=0.42	P adjusted=0.42	P adjusted=0.42	P adjusted=0.49	P adjusted=0.35	P adjusted=0.35	P adjusted=0.42
	P unadjusted=0.07	P unadjusted=0.06	P unadjusted=0.06	P unadjusted=0.04	P unadjusted=0.06	P unadjusted=0.06	P unadjusted=0.07	P unadjusted=0.06	P unadjusted=0.13	P unadjusted=0.06	P unadjusted=0.06	P unadjusted=0.06	P unadjusted=0.07	P unadjusted=0.05	P unadjusted=0.05	P unadjusted=0.06
**Cardiovascular death**	**HR 95%CI**	**HR 95%CI**	**HR 95%CI**	**HR 95%CI**	**HR 95%CI**	**HR 95%CI**	**HR 95%CI**	**HR 95%CI**	**HR 95%CI**	**HR 95%CI**	**HR 95%CI**	**HR 95%CI**	**HR 95%CI**	**HR 95%CI**	**HR 95%CI**	**HR 95%CI**
<120	0.90 (0.72 to 1.12)	0.90 (0.72 to 1.12)	0.90 (0.72 to 1.12)	0.90 (0.72 to 1.12)	0.90 (0.72 to 1.12)	0.90 (0.72 to 1.12)	0.90 (0.72 to 1.12)	0.90 (0.72 to 1.12)	0.90 (0.72 to 1.12)	0.90 (0.72 to 1.12)	0.90 (0.72 to 1.13)	0.90 (0.72 to 1.12)	0.90 (0.72 to 1.12)	0.89 (0.71 to 1.12)	0.89 (0.72 to 1.12)	0.90 (0.72 to 1.12)
120 to 129	0.89 (0.75 to 1.04)	0.88 (0.75 to 1.04)	0.89 (0.75 to 1.04)	0.89 (0.75 to 1.04)	0.89 (0.75 to 1.04)	0.89 (0.75 to 1.05)	0.89 (0.75 to 1.05)	0.89 (0.75 to 1.04)	0.89 (0.75 to 1.04)	0.89 (0.75 to 1.04)	0.87 (0.74 to 1.03)	0.89 (0.75 to 1.04)	0.89 (0.75 to 1.04)	0.89 (0.76 to 1.05)	0.89 (0.75 to 1.04)	0.89 (0.75 to 1.04)
130 to 139	0.99 (0.87 to 1.11)	0.98 (0.86 to 1.10)	0.99 (0.87 to 1.11)	0.98 (0.87 to 1.11)	0.98 (0.87 to 1.11)	0.98 (0.87 to 1.11)	0.98 (0.87 to 1.11)	0.98 (0.87 to 1.11)	0.98 (0.87 to 1.10)	0.98 (0.87 to 1.11)	0.98 (0.87 to 1.11)	0.98 (0.87 to 1.11)	0.98 (0.87 to 1.11)	0.98 (0.87 to 1.11)	0.98 (0.87 to 1.11)	0.98 (0.87 to 1.11)
140 to 149	0.98 (0.87 to 1.10)	0.98 (0.87 to 1.10)	0.97 (0.86 to 1.10)	0.97 (0.86 to 1.10)	0.97 (0.86 to 1.10)	0.97 (0.86 to 1.10)	0.97 (0.86 to 1.10)	0.97 (0.86 to 1.10)	0.97 (0.86 to 1.10)	0.97 (0.86 to 1.10)	0.98 (0.87 to 1.10)	0.97 (0.86 to 1.10)	0.97 (0.86 to 1.10)	0.97 (0.86 to 1.10)	0.97 (0.86 to 1.10)	0.98 (0.87 to 1.10)
150 to 159	1.00 (0.88 to 1.13)	1.00 (0.88 to 1.13)	1.00 (0.88 to 1.13)	1.00 (0.88 to 1.14)	1.00 (0.88 to 1.14)	1.00 (0.88 to 1.13)	1.00 (0.88 to 1.14)	1.00 (0.88 to 1.14)	1.00 (0.88 to 1.14)	1.00 (0.88 to 1.14)	1.00 (0.88 to 1.14)	1.00 (0.88 to 1.14)	1.00 (0.88 to 1.13)	1.00 (0.88 to 1.14)	1.00 (0.88 to 1.14)	1.00 (0.88 to 1.14)
160 to 169	0.90 (0.81 to 0.99)	0.90 (0.81 to 0.99)	0.90 (0.81 to 0.99)	0.90 (0.82 to 0.99)	0.91 (0.82 to 1.01)	0.90 (0.82 to 0.99)	0.90 (0.82 to 0.99)	0.90 (0.82 to 0.99)	0.91 (0.82 to 1.00)	0.91 (0.83 to 1.01)	0.91 (0.83 to 1.01)	0.90 (0.82 to 0.99)	0.90 (0.82 to 0.99)	0.91 (0.82 to 1.00)	0.90 (0.82 to 0.99)	0.90 (0.81 to 0.99)
≥170	0.95 (0.88 to 1.02)	0.95 (0.88 to 1.02)	0.95 (0.88 to 1.02)	0.95 (0.88 to 1.02)	0.95 (0.88 to 1.03)	0.94 (0.88 to 1.02)	0.95 (0.88 to 1.02)	0.95 (0.88 to 1.02)	0.95 (0.88 to 1.03)	0.95 (0.88 to 1.02)	0.94 (0.88 to 1.01)	0.94 (0.88 to 1.02)	0.96 (0.89 to 1.03)	0.94 (0.88 to 1.01)	0.95 (0.88 to 1.02)	0.95 (0.88 to 1.03)
p for interaction	P adjusted=0.49	P adjusted=0.42	P adjusted=0.49	P adjusted=0.49	P adjusted=0.49	P adjusted=0.56	P adjusted=0.49	P adjusted=0.49	P adjusted=0.63	P adjusted=0.63	P adjusted=0.42	P adjusted=0.56	P adjusted=0.56	P adjusted=0.70	P adjusted=0.49	P adjusted=0.42
	P unadjusted=0.07	P unadjusted=0.06	P unadjusted=0.07	P unadjusted=0.07	P unadjusted=0.07	P unadjusted=0.08	P unadjusted=0.07	P unadjusted=0.07	P unadjusted=0.09	P unadjusted=0.09	P unadjusted=0.06	P unadjusted=0.08	P unadjusted=0.08	P unadjusted=0.10	P unadjusted=0.07	P unadjusted=0.06
**All-cause death**	**HR 95%CI**	**HR 95%CI**	**HR 95%CI**	**HR 95%CI**	**HR 95%CI**	**HR 95%CI**	**HR 95%CI**	**HR 95%CI**	**HR 95%CI**	**HR 95%CI**	**HR 95%CI**	**HR 95%CI**	**HR 95%CI**	**HR 95%CI**	**HR 95%CI**	**HR 95%CI**
<120	0.88 (0.78 to 1.00)	0.88 (0.78 to 1.00)	0.88 (0.78 to 1.00)	0.91 (0.80 to 1.03)	0.88 (0.78 to 1.00)	0.88 (0.78 to 1.00)	0.88 (0.78 to 1.00)	0.89 (0.79 to 1.02)	0.88 (0.78 to 1.00)	0.88 (0.78 to 1.00)	0.88 (0.78 to 1.00)	0.88 (0.78 to 1.00)	0.88 (0.78 to 1.00)	0.88 (0.78 to 1.00)	0.88 (0.78 to 1.00)	0.88 (0.78 to 1.00)
120 to 129	1.00 (0.91 to 1.10)	1.00 (0.91 to 1.10)	1.00 (0.91 to 1.10)	1.00 (0.91 to 1.11)	1.00 (0.91 to 1.10)	1.00 (0.91 to 1.10)	1.00 (0.91 to 1.10)	0.99 (0.90 to 1.09)	1.00 (0.91 to 1.10)	1.00 (0.91 to 1.10)	1.00 (0.91 to 1.10)	1.00 (0.91 to 1.10)	1.00 (0.91 to 1.10)	1.00 (0.91 to 1.10)	1.00 (0.91 to 1.10)	1.00 (0.91 to 1.10)
130 to 139	0.98 (0.91 to 1.06)	0.98 (0.91 to 1.05)	0.98 (0.91 to 1.05)	0.99 (0.92 to 1.07)	0.98 (0.91 to 1.05)	0.98 (0.91 to 1.06)	0.98 (0.91 to 1.06)	0.99 (0.92 to 1.06)	0.98 (0.91 to 1.06)	0.98 (0.91 to 1.05)	0.98 (0.91 to 1.06)	0.98 (0.91 to 1.05)	0.98 (0.91 to 1.05)	0.98 (0.91 to 1.05)	0.98 (0.91 to 1.05)	0.98 (0.91 to 1.05)
140 to 149	0.99 (0.92 to 1.07)	0.99 (0.92 to 1.07)	0.99 (0.92 to 1.06)	1.00 (0.93 to 1.07)	0.99 (0.92 to 1.06)	0.99 (0.92 to 1.06)	0.99 (0.92 to 1.06)	0.99 (0.92 to 1.06)	0.99 (0.92 to 1.06)	0.99 (0.92 to 1.06)	0.99 (0.93 to 1.07)	0.99 (0.92 to 1.06)	0.99 (0.92 to 1.06)	0.99 (0.92 to 1.06)	0.99 (0.92 to 1.06)	0.99 (0.92 to 1.07)
150 to 159	0.95 (0.88 to 1.03)	0.95 (0.88 to 1.03)	0.95 (0.88 to 1.03)	0.96 (0.89 to 1.04)	0.95 (0.88 to 1.03)	0.95 (0.88 to 1.03)	0.95 (0.88 to 1.03)	0.97 (0.90 to 1.04)	0.95 (0.88 to 1.03)	0.95 (0.88 to 1.03)	0.95 (0.88 to 1.03)	0.95 (0.88 to 1.03)	0.95 (0.88 to 1.03)	0.95 (0.88 to 1.03)	0.95 (0.88 to 1.03)	0.95 (0.88 to 1.03)
160 to 169	0.94 (0.88 to 1.00)	0.94 (0.88 to 1.00)	0.94 (0.88 to 1.00)	0.94 (0.88 to 1.00)	0.94 (0.88 to 1.00)	0.94 (0.88 to 1.00)	0.94 (0.88 to 1.00)	0.94 (0.89 to 1.00)	0.95 (0.89 to 1.01)	0.94 (0.89 to 1.00)	0.94 (0.89 to 1.00)	0.94 (0.88 to 1.00)	0.94 (0.89 to 1.00)	0.94 (0.89 to 1.00)	0.94 (0.88 to 1.00)	0.94 (0.88 to 1.00)
≥170	0.97 (0.92 to 1.02)	0.97 (0.92 to 1.02)	0.97 (0.93 to 1.02)	0.97 (0.92 to 1.02)	0.97 (0.92 to 1.02)	0.97 (0.92 to 1.02)	0.97 (0.93 to 1.02)	0.98 (0.93 to 1.03)	0.98 (0.93 to 1.03)	0.97 (0.93 to 1.02)	0.97 (0.92 to 1.02)	0.97 (0.93 to 1.02)	0.97 (0.93 to 1.02)	0.97 (0.93 to 1.02)	0.97 (0.93 to 1.02)	0.97 (0.92 to 1.02)
p for interaction	P adjusted=1.00	P adjusted=1.00	P adjusted=1.00	P adjusted=1.00	P adjusted=1.00	P adjusted=1.00	P adjusted=1.00	P adjusted=1.00	P adjusted=1.00	P adjusted=1.00	P adjusted=1.00	P adjusted=1.00	P adjusted=1.00	P adjusted=1.00	P adjusted=1.00	P adjusted=1.00
	P unadjusted=0.24	P unadjusted=0.27	P unadjusted=0.32	P unadjusted=0.20	P unadjusted=0.33	P unadjusted=0.37	P unadjusted=0.33	P unadjusted=0.35	P unadjusted=0.40	P unadjusted=0.37	P unadjusted=0.25	P unadjusted=0.31	P unadjusted=0.33	P unadjusted=0.37	P unadjusted=0.32	P unadjusted=0.30

Table S4: Continues

Trial excluded from analysis

Baseline systolic blood pressure	ONTARGET	PART 2	PEACE	PREVEND IT	PREVENT	PROFESS	PROGRESS	SHEP
**Major cardiovascular events**	**HR 95%CI**	**HR 95%CI**	**HR 95%CI**	**HR 95%CI**	**HR 95%CI**	**HR 95%CI**	**HR 95%CI**	**HR 95%CI**
<120	0.80 (0.72 to 0.88)	0.78 (0.71 to 0.86)	0.79 (0.72 to 0.88)	0.79 (0.72 to 0.87)	0.79 (0.72 to 0.87)	0.79 (0.72 to 0.88)	0.78 (0.71 to 0.86)	0.79 (0.72 to 0.87)
120 to 129	0.90 (0.84 to 0.97)	0.91 (0.85 to 0.98)	0.92 (0.85 to 0.99)	0.91 (0.85 to 0.98)	0.91 (0.85 to 0.98)	0.92 (0.86 to 0.99)	0.92 (0.85 to 0.99)	0.91 (0.85 to 0.98)
130 to 139	0.93 (0.88 to 0.99)	0.94 (0.89 to 0.99)	0.94 (0.89 to 1.00)	0.94 (0.89 to 1.00)	0.94 (0.89 to 1.00)	0.93 (0.88 to 0.98)	0.94 (0.86 to 0.99)	0.94 (0.89 to 0.99)
140 to 149	0.92 (0.87 to 0.97)	0.93 (0.88 to 0.98)	0.92 (0.87 to 0.97)	0.93 (0.88 to 0.98)	0.93 (0.88 to 0.98)	0.93 (0.89 to 0.98)	0.94 (0.89 to 1.00)	0.93 (0.88 to 0.98)
150 to 159	0.89 (0.84 to 0.95)	0.89 (0.84 to 0.94)	0.89 (0.83 to 0.94)	0.89 (0.83 to 0.94)	0.89 (0.84 to 0.94)	0.88 (0.83 to 0.94)	0.89 (0.83 to 0.94)	0.89 (0.83 to 0.94)
160 to 169	0.87 (0.82 to 0.92)	0.87 (0.83 to 0.92)	0.87 (0.83 to 0.92)	0.87 (0.83 to 0.92)	0.87 (0.83 to 0.92)	0.86 (0.82 to 0.91)	0.89 (0.84 to 0.94)	0.85 (0.80 to 0.90)
≥170	0.90 (0.86 to 0.94)	0.90 (0.86 to 0.94)	0.90 (0.87 to 0.95)	0.90 (0.87 to 0.94)	0.90 (0.87 to 0.94)	0.90 (0.86 to 0.94)	0.91 (0.87 to 0.95)	0.89 (0.85 to 0.93)
p for interaction	P adjusted=1.00	P adjusted=0.28	P adjusted=0.70	P adjusted=0.35	P adjusted=0.35	P adjusted=0.42	P adjusted=0.28	P adjusted=0.28
	P unadjusted=0.20	P unadjusted=0.04	P unadjusted=0.10	P unadjusted=0.05	P unadjusted=0.05	P unadjusted=0.06	P unadjusted=0.04	P unadjusted=0.04
**Stroke**	**HR 95%CI**	**HR 95%CI**	**HR 95%CI**	**HR 95%CI**	**HR 95%CI**	**HR 95%CI**	**HR 95%CI**	**HR 95%CI**
<120	0.80 (0.64 to 0.98)	0.79 (0.64 to 0.97)	0.80 (0.65 to 0.98)	0.80 (0.65 to 0.98)	0.80 (0.65 to 0.97)	0.80 (0.64 to 0.98)	0.77 (0.62 to 0.95)	0.79 (0.65 to 0.97)
120 to 129	0.85 (0.74 to 0.99)	0.86 (0.74 to 0.99)	0.87 (0.75 to 1.00)	0.86 (0.75 to 0.99)	0.85 (0.74 to 0.98)	0.84 (0.72 to 0.99)	0.87 (0.74 to 1.03)	0.86 (0.74 to 0.99)
130 to 139	0.91 (0.82 to 1.01)	0.92 (0.83 to 1.02)	0.93 (0.84 to 1.03)	0.93 (0.83 to 1.03)	0.92 (0.83 to 1.02)	0.88 (0.78 to 0.99)	0.90 (0.80 to 1.01)	0.92 (0.83 to 1.02)
140 to 149	0.90 (0.81 to 0.99)	0.90 (0.82 to 1.00)	0.90 (0.81 to 0.99)	0.90 (0.82 to 0.99)	0.90 (0.82 to 0.99)	0.91 (0.82 to 1.01)	0.92 (0.82 to 1.03)	0.90 (0.82 to 0.99)
150 to 159	0.79 (0.71 to 0.88)	0.77 (0.70 to 0.86)	0.78 (0.70 to 0.87)	0.78 (0.70 to 0.86)	0.78 (0.70 to 0.86)	0.78 (0.70 to 0.87)	0.78 (0.70 to 0.89)	0.77 (0.70 to 0.86)
160 to 169	0.85 (0.78 to 0.93)	0.86 (0.79 to 0.94)	0.86 (0.79 to 0.94)	0.86 (0.79 to 0.94)	0.86 (0.79 to 0.94)	0.85 (0.78 to 0.93)	0.90 (0.82 to 0.99)	0.84 (0.76 to 0.93)
≥170	0.90 (0.84 to 0.96)	0.90 (0.84 to 0.97)	0.90 (0.84 to 0.97)	0.91 (0.85 to 0.97)	0.91 (0.85 to 0.97)	0.90 (0.84 to 0.97)	0.91 (0.85 to 0.97)	0.89 (0.82 to 0.95)
p for *interaction*	P adjusted=1.00	P adjusted=0.70	P adjusted=0.91	P adjusted=0.84	P adjusted=0.84	P adjusted=1.00	P adjusted=0.84	P adjusted=0.84
	P unadjusted=0.38	P unadjusted=0.10	P unadjusted=0.13	P unadjusted=0.12	P unadjusted=0.12	P unadjusted=0.23	P unadjusted=0.14	P unadjusted=0.12
**Ischaemic heart disease**	**HR 95%CI**	**HR 95%CI**	**HR 95%CI**	**HR 95%CI**	**HR 95%CI**	**HR 95%CI**	**HR 95%CI**	**HR 95%CI**
<120	0.86 (0.75 to 0.99)	0.82 (0.72 to 0.94)	0.86 (0.75 to 0.99)	0.83 (0.73 to 0.96)	0.83 (0.72 to 0.95)	0.83 (0.73 to 0.96)	0.82 (0.72 to 0.95)	0.83 (0.73 to 0.96)
120 to 129	0.95 (0.86 to 1.06)	0.95 (0.86 to 1.06)	0.95 (0.86 to 1.06)	0.95 (0.86 to 1.05)	0.95 (0.86 to 1.06)	0.95 (0.86 to 1.05)	0.96 (0.87 to 1.07)	0.95 (0.86 to 1.05)
130 to 139	0.96 (0.89 to 1.04)	0.97 (0.89 to 1.05)	0.96 (0.89 to 1.04)	0.96 (0.89 to 1.05)	0.96 (0.89 to 1.05)	0.97 (0.89 to 1.05)	0.98 (0.90 to 1.07)	0.96 (0.89 to 1.05)
140 to 149	0.93 (0.86 to 1.00)	0.94 (0.87 to 1.02)	0.94 (0.87 to 1.01)	0.94 (0.87 to 1.02)	0.94 (0.87 to 1.01)	0.94 (0.87 to 1.01)	0.95 (0.88 to 1.03)	0.94 (0.87 to 1.02)
150 to 159	0.90 (0.83 to 0.98)	0.91 (0.84 to 0.99)	0.90 (0.83 to 0.98)	0.91 (0.83 to 0.99)	0.91 (0.83 to 0.99)	0.90 (0.82 to 0.98)	0.90 (0.83 to 0.98)	0.91 (0.83 to 0.99)
160 to 169	0.87 (0.80 to 0.94)	0.87 (0.80 to 0.95)	0.87 (0.80 to 0.94)	0.87 (0.80 to 0.94)	0.87 (0.80 to 0.95)	0.87 (0.80 to 0.95)	0.88 (0.81 to 0.96)	0.86 (0.79 to 0.94)
≥170	0.94 (0.87 to 1.00)	0.94 (0.87 to 1.00)	0.94 (0.87 to 1.00)	0.94 (0.87 to 1.00)	0.94 (0.87 to 1.00)	0.94 (0.87 to 1.01)	0.93 (0.86 to 0.99)	0.93 (0.87 to 1.00)
p for interaction	P adjusted=1.00	P adjusted=1.00	P adjusted=1.00	P adjusted=1.00	P adjusted=1.00	P adjusted=1.00	P adjusted=1.00	P adjusted=1.00
	P unadjusted=0.93	P unadjusted=0.65	P unadjusted=0.90	P unadjusted=0.71	P unadjusted=0.38	P unadjusted=0.77	P unadjusted=0.69	P unadjusted=0.72
**Heart failure**	**HR 95%CI**	**HR 95%CI**	**HR 95%CI**	**HR 95%CI**	**HR 95%CI**	**HR 95%CI**	**HR 95%CI**	**HR 95%CI**
<120	0.78 (0.64 to 0.96)	0.78 (0.64 to 0.95)	0.78 (0.64 to 0.96)	0.78 (0.64 to 0.96)	0.79 (0.64 to 0.96)	0.79 (0.64 to 0.96)	0.79 (0.64 to 0.96)	0.78 (0.64 to 0.96)
120 to 129	0.99 (0.85 to 1.15)	0.99 (0.85 to 1.15)	1.02 (0.87 to 1.19)	0.98 (0.84 to 1.14)	0.99 (0.85 to 1.15)	1.02 (0.87 to 1.19)	0.97 (0.83 to 1.13)	0.99 (0.85 to 1.15)
130 to 139	0.85 (0.75 to 0.97)	0.85 (0.75 to 0.97)	0.86 (0.75 to 0.98)	0.85 (0.75 to 0.97)	0.85 (0.74 to 0.97)	0.82 (0.72 to 0.94)	0.85 (0.74 to 0.97)	0.85 (0.74 to 0.96)
140 to 149	0.95 (0.84 to 1.07)	0.95 (0.85 to 1.07)	0.94 (0.84 to 1.06)	0.96 (0.85 to 1.08)	0.95 (0.85 to 1.07)	0.95 (0.84 to 1.07)	0.99 (0.87 to 1.11)	0.95 (0.85 to 1.07)
150 to 159	0.89 (0.78 to 1.00)	0.87 (0.78 to 1.00)	0.89 (0.78 to 1.01)	0.89 (0.78 to 1.01)	0.89 (0.78 to 1.01)	0.87 (0.77 to 0.99)	0.87 (0.77 to 1.00)	0.88 (0.78 to 1.00)
160 to 169	0.70 (0.61 to 0.80)	0.70 (0.60 to 0.80)	0.70 (0.61 to 0.81)	0.70 (0.61 to 0.80)	0.70 (0.61 to 0.80)	0.68 (0.59 to 0.78)	0.70 (0.59 to 0.80)	0.70 (0.60 to 0.80)
≥170	0.90 (0.80 to 1.01)	0.89 (0.79 to 1.00)	0.89 (0.80 to 1.00)	0.89 (0.79 to 1.00)	0.89 (0.79 to 1.00)	0.89 (0.80 to 1.01)	0.89 (0.79 to 1.01)	0.89 (0.80 to 1.00)
p for interaction	P adjusted=0.77	P adjusted=0.42	P adjusted=0.48	P adjusted=0.48	P adjusted=0.42	P adjusted=0.14	P adjusted=0.42	P adjusted=0.42
	P unadjusted=0.11	P unadjusted=0.06	P unadjusted=0.07	P unadjusted=0.07	P unadjusted=0.06	P unadjusted=0.02	P unadjusted=0.06	P unadjusted=0.06
**Cardiovascular death**	**HR 95%CI**	**HR 95%CI**	**HR 95%CI**	**HR 95%CI**	**HR 95%CI**	**HR 95%CI**	**HR 95%CI**	**HR 95%CI**
<120	0.92 (0.73 to 1.16)	0.88 (0.70 to 1.11)	0.90 (0.71 to 1.12)	0.90 (0.72 to 1.12)	0.91 (0.73 to 1.14)	0.94 (0.75 to 1.17)	0.89 (0.70 to 1.12)	0.90 (0.72 to 1.12)
120 to 129	0.87 (0.73 to 1.02)	0.89 (0.76 to 1.05)	0.89 (0.75 to 1.06)	0.89 (0.75 to 1.05)	0.89 (0.75 to 1.05)	0.90 (0.76 to 1.06)	0.86 (0.72 to 1.03)	0.89 (0.75 to 1.04)
130 to 139	0.98 (0.86 to 1.11)	0.99 (0.87 to 1.11)	0.98 (0.87 to 1.11)	0.98 (0.86 to 1.11)	0.98 (0.86 to 1.10)	0.95 (0.83 to 1.08)	0.98 (0.86 to 1.12)	0.98 (0.87 to 1.11)
140 to 149	0.96 (0.86 to 1.09)	0.98 (0.87 to 1.10)	0.97 (0.85 to 1.08)	0.98 (0.86 to 1.10)	0.97 (0.86 to 1.10)	0.94 (0.83 to 1.07)	1.02 (0.87 to 1.16)	0.97 (0.86 to 1.10)
150 to 159	1.00 (0.88 to 1.14)	1.00 (0.89 to 1.14)	1.00 (0.88 to 1.14)	1.00 (0.88 to 1.13)	1.01 (0.89 to 1.15)	0.99 (0.87 to 1.13)	0.97 (0.84 to 1.12)	1.00 (0.88 to 1.14)
160 to 169	0.89 (0.80 to 0.99)	0.90 (0.82 to 1.00)	0.90 (0.82 to 1.00)	0.90 (0.82 to 1.00)	0.90 (0.82 to 1.00)	0.89 (0.80 to 0.98)	0.92 (0.83 to 1.02)	0.86 (0.77 to 0.95)
≥170	0.94 (0.88 to 1.02)	0.95 (0.88 to 1.01)	0.95 (0.88 to 1.02)	0.95 (0.88 to 1.02)	0.95 (0.88 to 1.02)	0.93 (0.87 to 1.01)	0.93 (0.87 to 1.01)	0.93 (0.87 to 1.01)
p for interaction	P adjusted=0.35	P adjusted=0.48	P adjusted=0.98	P adjusted=0.48	P adjusted=0.48	P adjusted=0.98	P adjusted=0.28	P adjusted=0.28
	P unadjusted=0.05	P unadjusted=0.07	P unadjusted=0.14	P unadjusted=0.07	P unadjusted=0.07	P unadjusted=0.14	P unadjusted=0.04	P unadjusted=0.04
**All-cause death**	**HR 95%CI**	**HR 95%CI**	**HR 95%CI**	**HR 95%CI**	**HR 95%CI**	**HR 95%CI**	**HR 95%CI**	**HR 95%CI**
<120	0.87 (0.76 to 0.99)	0.87 (0.77 to 0.99)	0.90 (0.79 to 1.02)	0.88 (0.78 to 1.00)	0.88 (0.78 to 1.00)	0.90 (0.79 to 1.02)	0.86 (0.76 to 0.98)	0.88 (0.78 to 1.00)
120 to 129	1.00 (0.90 to 1.09)	1.00 (0.91 to 1.10)	1.00 (0.91 to 1.11)	1.00 (0.91 to 1.10)	1.00 (0.91 to 1.10)	1.01 (0.92 to 1.12)	0.98 (0.89 to 1.07)	1.00 (0.91 to 1.10)
130 to 139	0.97 (0.90 to 1.05)	0.98 (0.91 to 1.06)	0.98 (0.91 to 1.06)	0.98 (0.91 to 1.06)	0.98 (0.91 to 1.05)	0.96 (0.89 to 1.03)	0.98 (0.90 to 1.06)	0.98 (0.91 to 1.05)
140 to 149	0.98 (0.91 to 1.06)	0.99 (0.92 to 1.07)	0.99 (0.92 to 1.06)	0.99 (0.92 to 1.07)	0.99 (0.92 to 1.06)	0.98 (0.91 to 1.05)	1.00 (0.93 to 1.08)	0.99 (0.92 to 1.06)
150 to 159	0.95 (0.88 to 1.02)	0.96 (0.89 to 1.03)	0.95 (0.88 to 1.03)	0.95 (0.88 to 1.03)	0.96 (0.89 to 1.03)	0.95 (0.88 to 1.03)	0.93 (0.85 to 1.00)	0.95 (0.88 to 1.03)
160 to 169	0.95 (0.88 to 1.00)	0.94 (0.88 to 1.00)	0.94 (0.87 to 1.00)	0.94 (0.88 to 1.00)	0.94 (0.88 to 1.00)	0.94 (0.88 to 1.00)	0.95 (0.90 to 1.02)	0.91 (0.86 to 0.98)
≥170	0.97 (0.92 to 1.02)	0.97 (0.93 to 1.02)	0.98 (0.93 to 1.03)	0.97 (0.93 to 1.02)	0.97 (0.93 to 1.02)	0.97 (0.92 to 1.02)	0.97 (0.92 to 1.02)	0.96 (0.91 to 1.01)
p for *interaction*	P adjusted=1.00	P adjusted=1.00	P adjusted=1.00	P adjusted=1.00	P adjusted=1.00	P adjusted=1.00	P adjusted=1.00	P adjusted=1.00
	P unadjusted=0.63	P unadjusted=0.30	P unadjusted=0.44	P unadjusted=0.32	P unadjusted=0.32	P unadjusted=0.39	P unadjusted=0.34	P unadjusted=0.17

Baseline systolic blood pressure	SPRINT	STOP HYPERTENSION-2	SYST-EUR	TRANSCEND	UKPDS	VALISH	VALUE	VHAS
**Major cardiovascular events**	**HR 95%CI**	**HR 95%CI**	**HR 95%CI**	**HR 95%CI**	**HR 95%CI**	**HR 95%CI**	**HR 95%CI**	**HR 95%CI**
<120	0.81 (0.73 to 0.90)	0.79 (0.72 to 0.87)	0.79 (0.72 to 0.87)	0.79 (0.71 to 0.87)	0.79 (0.72 to 0.87)	0.79 (0.72 to 0.87)	0.79 (0.71 to 0.87)	0.79 (0.72 to 0.87)
120 to 129	0.91 (0.84 to 0.98)	0.91 (0.85 to 0.98)	0.91 (0.85 to 0.98)	0.91 (0.84 to 0.97)	0.91 (0.85 to 0.98)	0.91 (0.85 to 0.98)	0.91 (0.85 to 0.98)	0.91 (0.85 to 0.98)
130 to 139	0.97 (0.91 to 1.03)	0.94 (0.89 to 0.99)	0.94 (0.89 to 0.99)	0.94 (0.89 to 1.00)	0.94 (0.89 to 1.00)	0.94 (0.89 to 1.00)	0.93 (0.88 to 0.99)	0.94 (0.89 to 0.99)
140 to 149	0.91 (0.86 to 0.97)	0.93 (0.88 to 0.98)	0.93 (0.88 to 0.98)	0.92 (0.88 to 0.98)	0.94 (0.87 to 0.99)	0.93 (0.88 to 0.98)	0.93 (0.88 to 0.98)	0.93 (0.88 to 0.98)
150 to 159	0.88 (0.83 to 0.94)	0.88 (0.83 to 0.94)	0.88 (0.83 to 0.94)	0.89 (0.84 to 0.94)	0.87 (0.83 to 0.94)	0.88 (0.83 to 0.94)	0.88 (0.83 to 0.94)	0.88 (0.83 to 0.94)
160 to 169	0.87 (0.82 to 0.91)	0.87 (0.83 to 0.92)	0.87 (0.83 to 0.92)	0.88 (0.83 to 0.92)	0.88 (0.83 to 0.92)	0.87 (0.82 to 0.92)	0.87 (0.82 to 0.92)	0.87 (0.83 to 0.92)
≥170	0.90 (0.86 to 0.94)	0.89 (0.85 to 0.94)	0.90 (0.86 to 0.95)	0.90 (0.86 to 0.95)	0.90 (0.86 to 0.94)	0.90 (0.87 to 0.95)	0.90 (0.87 to 0.95)	0.90 (0.86 to 0.94)
p for interaction	P adjusted=0.42	P adjusted=0.28	P adjusted=0.35	P adjusted=0.42	P adjusted=0.28	P adjusted=0.35	P adjusted=0.28	P adjusted=0.35
	P unadjusted=0.06	P unadjusted=0.04	P unadjusted=0.05	P unadjusted=0.06	P unadjusted=0.04	P unadjusted=0.05	P unadjusted=0.04	P unadjusted=0.05
**Stroke**	**HR 95%CI**	**HR 95%CI**	**HR 95%CI**	**HR 95%CI**	**HR 95%CI**	**HR 95%CI**	**HR 95%CI**	**HR 95%CI**
<120	0.81 (0.65 to 1.00)	0.79 (0.65 to 0.97)	0.79 (0.65 to 0.97)	0.78 (0.64 to 0.96)	0.79 (0.65 to 0.97)	0.79 (0.65 to 0.97)	0.79 (0.64 to 0.96)	0.79 (0.65 to 0.97)
120 to 129	0.82 (0.71 to 0.96)	0.86 (0.74 to 0.99)	0.86 (0.74 to 0.99)	0.85 (0.73 to 0.98)	0.86 (0.75 to 0.99)	0.86 (0.74 to 0.99)	0.86 (0.75 to 1.00)	0.86 (0.74 to 0.99)
130 to 139	0.93 (0.83 to 1.04)	0.92 (0.83 to 1.02)	0.92 (0.83 to 1.03)	0.94 (0.85 to 1.05)	0.94 (0.84 to 1.04)	0.92 (0.83 to 1.02)	0.92 (0.83 to 1.02)	0.92 (0.83 to 1.03)
140 to 149	0.86 (0.77 to 0.96)	0.90 (0.82 to 0.99)	0.90 (0.82 to 0.99)	0.89 (0.81 to 0.98)	0.91 (0.83 to 1.00)	0.90 (0.82 to 0.99)	0.91 (0.82 to 1.00)	0.90 (0.82 to 0.99)
150 to 159	0.77 (0.68 to 0.86)	0.77 (0.70 to 0.86)	0.77 (0.70 to 0.86)	0.76 (0.68 to 0.85)	0.78 (0.70 to 0.87)	0.78 (0.70 to 0.86)	0.78 (0.70 to 0.86)	0.77 (0.70 to 0.86)
160 to 169	0.85 (0.78 to 0.93)	0.86 (0.79 to 0.94)	0.87 (0.80 to 0.95)	0.87 (0.80 to 0.95)	0.86 (0.80 to 0.94)	0.86 (0.79 to 0.94)	0.87 (0.79 to 0.94)	0.86 (0.79 to 0.94)
≥170	0.91 (0.85 to 0.98)	0.88 (0.82 to 0.95)	0.91 (0.85 to 0.98)	0.91 (0.85 to 0.97)	0.91 (0.85 to 0.97)	0.91 (0.85 to 0.97)	0.91 (0.84 to 0.97)	0.90 (0.84 to 0.97)
p for *interaction*	P adjusted=0.70	P adjusted=1.00	P adjusted=0.63	P adjusted=0.42	P adjusted=0.63	P adjusted=0.70	P adjusted=0.84	P adjusted=0.77
	P unadjusted=0.10	P unadjusted=0.15	P unadjusted=0.09	P unadjusted=0.06	P unadjusted=0.09	P unadjusted=0.10	P unadjusted=0.12	P unadjusted=0.11
**Ischaemic heart disease**	**HR 95%CI**	**HR 95%CI**	**HR 95%CI**	**HR 95%CI**	**HR 95%CI**	**HR 95%CI**	**HR 95%CI**	**HR 95%CI**
<120	0.84 (0.73 to 0.98)	0.83 (0.73 to 0.96)	0.83 (0.73 to 0.96)	0.85 (0.74 to 0.97)	0.83 (0.73 to 0.97)	0.83 (0.73 to 0.96)	0.83 (0.72 to 0.95)	0.83 (0.73 to 0.96)
120 to 129	0.94 (0.85 to 1.05)	0.95 (0.86 to 1.05)	0.95 (0.86 to 1.05)	0.95 (0.86 to 1.06)	0.95 (0.86 to 1.05)	0.95 (0.86 to 1.05)	0.96 (0.86 to 1.06)	0.95 (0.86 to 1.05)
130 to 139	0.99 (0.90 to 1.08)	0.97 (0.89 to 1.05)	0.96 (0.89 to 1.05)	0.96 (0.89 to 1.05)	0.96 (0.88 to 1.04)	0.96 (0.89 to 1.05)	0.97 (0.89 to 1.05)	0.96 (0.89 to 1.05)
140 to 149	0.93 (0.86 to 1.02)	0.94 (0.87 to 1.02)	0.94 (0.87 to 1.02)	0.94 (0.87 to 1.01)	0.95 (0.88 to 1.03)	0.94 (0.87 to 1.02)	0.95 (0.87 to 1.02)	0.94 (0.87 to 1.02)
150 to 159	0.90 (0.82 to 0.98)	0.91 (0.83 to 0.99)	0.91 (0.83 to 0.99)	0.92 (0.85 to 1.00)	0.90 (0.83 to 0.99)	0.91 (0.83 to 0.99)	0.91 (0.83 to 0.99)	0.91 (0.83 to 0.99)
160 to 169	0.86 (0.79 to 0.94)	0.87 (0.80 to 0.95)	0.87 (0.80 to 0.95)	0.87 (0.80 to 0.95)	0.89 (0.81 to 0.96)	0.87 (0.80 to 0.95)	0.87 (0.80 to 0.94)	0.87 (0.80 to 0.94)
≥170	0.92 (0.86 to 0.99)	0.94 (0.87 to 1.00)	0.94 (0.88 to 1.01)	0.94 (0.88 to 1.00)	0.93 (0.87 to 1.00)	0.93 (0.87 to 1.00)	0.95 (0.88 to 1.02)	0.94 (0.87 to 1.00)
p for interaction	P adjusted=1.00	P adjusted=1.00	P adjusted=1.00	P adjusted=1.00	P adjusted=1.00	P adjusted=1.00	P adjusted=1.00	P adjusted=1.00
	P unadjusted=0.72	P unadjusted=0.72	P unadjusted=0.74	P unadjusted=0.73	P unadjusted=0.75	P unadjusted=0.72	P unadjusted=0.53	P unadjusted=0.70
**Heart failure**	**HR 95%CI**	**HR 95%CI**	**HR 95%CI**	**HR 95%CI**	**HR 95%CI**	**HR 95%CI**	**HR 95%CI**	**HR 95%CI**
<120	0.78 (0.62 to 0.98)	0.78 (0.64 to 0.96)	0.78 (0.64 to 0.96)	0.76 (0.62 to 0.94)	0.78 (0.64 to 0.96)	0.78 (0.64 to 0.96)	0.78 (0.64 to 0.96)	0.78 (0.64 to 0.96)
120 to 129	1.03 (0.88 to 1.22)	0.99 (0.85 to 1.15)	0.99 (0.85 to 1.15)	0.96 (0.82 to 1.12)	0.99 (0.85 to 1.15)	0.99 (0.85 to 1.15)	0.98 (0.84 to 1.15)	0.99 (0.85 to 1.15)
130 to 139	0.91 (0.78 to 1.05)	0.85 (0.74 to 0.96)	0.85 (0.74 to 0.96)	0.84 (0.74 to 0.96)	0.85 (0.74 to 0.96)	0.85 (0.74 to 0.96)	0.81 (0.71 to 0.93)	0.85 (0.74 to 0.96)
140 to 149	0.96 (0.84 to 1.10)	0.95 (0.85 to 1.07)	0.95 (0.85 to 1.07)	0.94 (0.84 to 1.06)	0.95 (0.85 to 1.07)	0.95 (0.85 to 1.07)	0.95 (0.84 to 1.07)	0.95 (0.85 to 1.07)
150 to 159	0.88 (0.76 to 1.02)	0.88 (0.78 to 1.00)	0.88 (0.78 to 1.00)	0.88 (0.78 to 1.00)	0.88 (0.78 to 1.00)	0.88 (0.78 to 1.00)	0.87 (0.76 to 0.99)	0.88 (0.78 to 1.00)
160 to 169	0.70 (0.60 to 0.81)	0.70 (0.61 to 0.80)	0.69 (0.60 to 0.80)	0.70 (0.61 to 0.81)	0.70 (0.61 to 0.80)	0.70 (0.60 to 0.80)	0.69 (0.60 to 0.80)	0.70 (0.60 to 0.80)
≥170	0.90 (0.80 to 1.02)	0.90 (0.80 to 1.02)	0.90 (0.80 to 1.01)	0.89 (0.80 to 1.00)	0.89 (0.80 to 1.00)	0.90 (0.80 to 1.00)	0.86 (0.76 to 0.97)	0.90 (0.79 to 1.00)
p for interaction	P adjusted=0.42	P adjusted=0.42	P adjusted=0.35	P adjusted=0.98	P adjusted=0.42	P adjusted=0.42	P adjusted=0.28	P adjusted=0.42
	P unadjusted=0.06	P unadjusted=0.06	P unadjusted=0.05	P unadjusted=0.14	P unadjusted=0.06	P unadjusted=0.06	P unadjusted=0.04	P unadjusted=0.06
**Cardiovascular death**	**HR 95%CI**	**HR 95%CI**	**HR 95%CI**	**HR 95%CI**	**HR 95%CI**	**HR 95%CI**	**HR 95%CI**	**HR 95%CI**
<120	0.99 (0.77 to 1.27)	0.90 (0.72 to 1.12)	0.90 (0.72 to 1.12)	0.89 (0.70 to 1.12)	0.90 (0.72 to 1.12)	0.90 (0.72 to 1.13)	0.89 (0.71 to 1.11)	0.90 (0.72 to 1.12)
120 to 129	0.89 (0.74 to 1.06)	0.89 (0.75 to 1.04)	0.89 (0.75 to 1.04)	0.89 (0.75 to 1.05)	0.89 (0.75 to 1.04)	0.88 (0.75 to 1.04)	0.88 (0.75 to 1.04)	0.89 (0.75 to 1.04)
130 to 139	1.02 (0.89 to 1.16)	0.98 (0.87 to 1.11)	0.98 (0.87 to 1.11)	0.98 (0.86 to 1.11)	0.98 (0.87 to 1.11)	0.98 (0.87 to 1.11)	0.98 (0.87 to 1.11)	0.98 (0.87 to 1.11)
140 to 149	0.98 (0.86 to 1.11)	0.97 (0.86 to 1.10)	0.97 (0.86 to 1.10)	0.97 (0.86 to 1.10)	0.97 (0.86 to 1.10)	0.97 (0.86 to 1.10)	0.97 (0.86 to 1.10)	0.97 (0.86 to 1.10)
150 to 159	1.04 (0.91 to 1.19)	1.00 (0.88 to 1.13)	1.00 (0.88 to 1.14)	1.02 (0.89 to 1.15)	1.00 (0.88 to 1.14)	1.00 (0.88 to 1.14)	1.00 (0.88 to 1.14)	1.00 (0.88 to 1.14)
160 to 169	0.90 (0.82 to 1.00)	0.90 (0.82 to 1.00)	0.90 (0.82 to 1.00)	0.90 (0.82 to 1.00)	0.90 (0.82 to 1.00)	0.90 (0.81 to 0.99)	0.90 (0.81 to 0.99)	0.90 (0.81 to 0.99)
≥170	0.95 (0.88 to 1.02)	0.94 (0.87 to 1.02)	0.95 (0.88 to 1.03)	0.94 (0.88 to 1.02)	0.95 (0.88 to 1.02)	0.95 (0.88 to 1.02)	0.95 (0.88 to 1.02)	0.95 (0.88 to 1.02)
p for interaction	P adjusted=0.28	P adjusted=0.42	P adjusted=0.56	P adjusted=0.48	P adjusted=0.48	P adjusted=0.42	P adjusted=0.35	P adjusted=0.48
	P unadjusted=0.04	P unadjusted=0.06	P unadjusted=0.08	P unadjusted=0.07	P unadjusted=0.07	P unadjusted=0.06	P unadjusted=0.05	P unadjusted=0.07
**All-cause death**	**HR 95%CI**	**HR 95%CI**	**HR 95%CI**	**HR 95%CI**	**HR 95%CI**	**HR 95%CI**	**HR 95%CI**	**HR 95%CI**
<120	0.86 (0.74 to 0.99)	0.88 (0.78 to 1.00)	0.88 (0.78 to 1.00)	0.87 (0.76 to 0.99)	0.88 (0.78 to 1.00)	0.88 (0.78 to 1.00)	0.88 (0.78 to 1.00)	0.88 (0.78 to 1.00)
120 to 129	1.04 (0.93 to 1.16)	1.00 (0.91 to 1.10)	1.00 (0.91 to 1.10)	1.00 (0.91 to 1.11)	1.00 (0.91 to 1.10)	1.00 (0.91 to 1.10)	1.00 (0.91 to 1.10)	1.00 (0.91 to 1.10)
130 to 139	1.02 (0.94 to 1.11)	0.98 (0.91 to 1.05)	0.98 (0.91 to 1.05)	0.98 (0.91 to 1.06)	0.98 (0.91 to 1.05)	0.98 (0.91 to 1.05)	0.98 (0.91 to 1.05)	0.98 (0.91 to 1.05)
140 to 149	1.00 (0.93 to 1.09)	0.99 (0.92 to 1.06)	0.99 (0.92 to 1.06)	0.97 (0.91 to 1.05)	0.99 (0.92 to 1.07)	0.99 (0.92 to 1.06)	1.00 (0.93 to 1.08)	0.99 (0.92 to 1.06)
150 to 159	0.96 (0.88 to 1.05)	0.95 (0.88 to 1.03)	0.95 (0.88 to 1.03)	0.95 (0.88 to 1.03)	0.96 (0.89 to 1.04)	0.95 (0.88 to 1.02)	0.96 (0.89 to 1.03)	0.95 (0.88 to 1.02)
160 to 169	0.93 (0.88 to 0.99)	0.94 (0.89 to 1.00)	0.94 (0.89 to 1.00)	0.94 (0.88 to 1.00)	0.95 (0.89 to 1.01)	0.94 (0.89 to 1.00)	0.93 (0.87 to 0.99)	0.94 (0.89 to 1.00)
≥170	0.97 (0.93 to 1.02)	0.97 (0.92 to 1.02)	0.97 (0.92 to 1.02)	0.97 (0.93 to 1.02)	0.97 (0.92 to 1.02)	0.97 (0.93 to 1.02)	0.97 (0.93 to 1.03)	0.97 (0.93 to 1.02)
p for *interaction*	P adjusted=1.00	P adjusted=1.00	P adjusted=1.00	P adjusted=1.00	P adjusted=1.00	P adjusted=1.00	P adjusted=0.70	P adjusted=1.00
	P unadjusted=0.19	P unadjusted=0.33	P unadjusted=0.36	P unadjusted=0.42	P unadjusted=0.30	P unadjusted=0.32	P unadjusted=0.10	P unadjusted=0.31

Figure S1. Kaplan–Meier rates of stroke per 5 mmHg reduction in systolic blood pressure, stratified by treatment allocation and cardiovascular disease status at baseline.

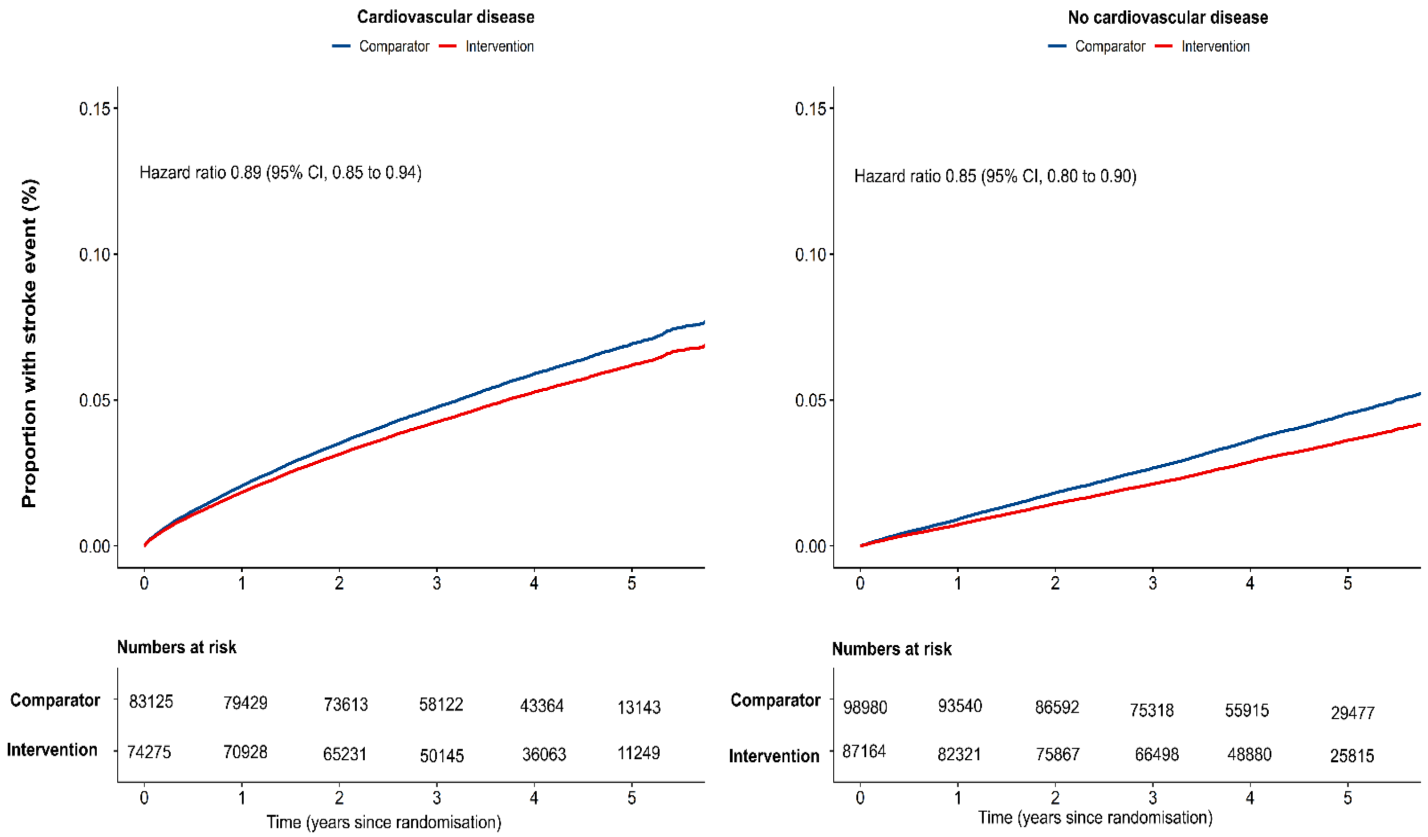

Figure S2. Kaplan–Meier rates of ischaemic heart disease per 5 mmHg reduction in systolic blood pressure, stratified by treatment allocation and cardiovascular disease status at baseline.

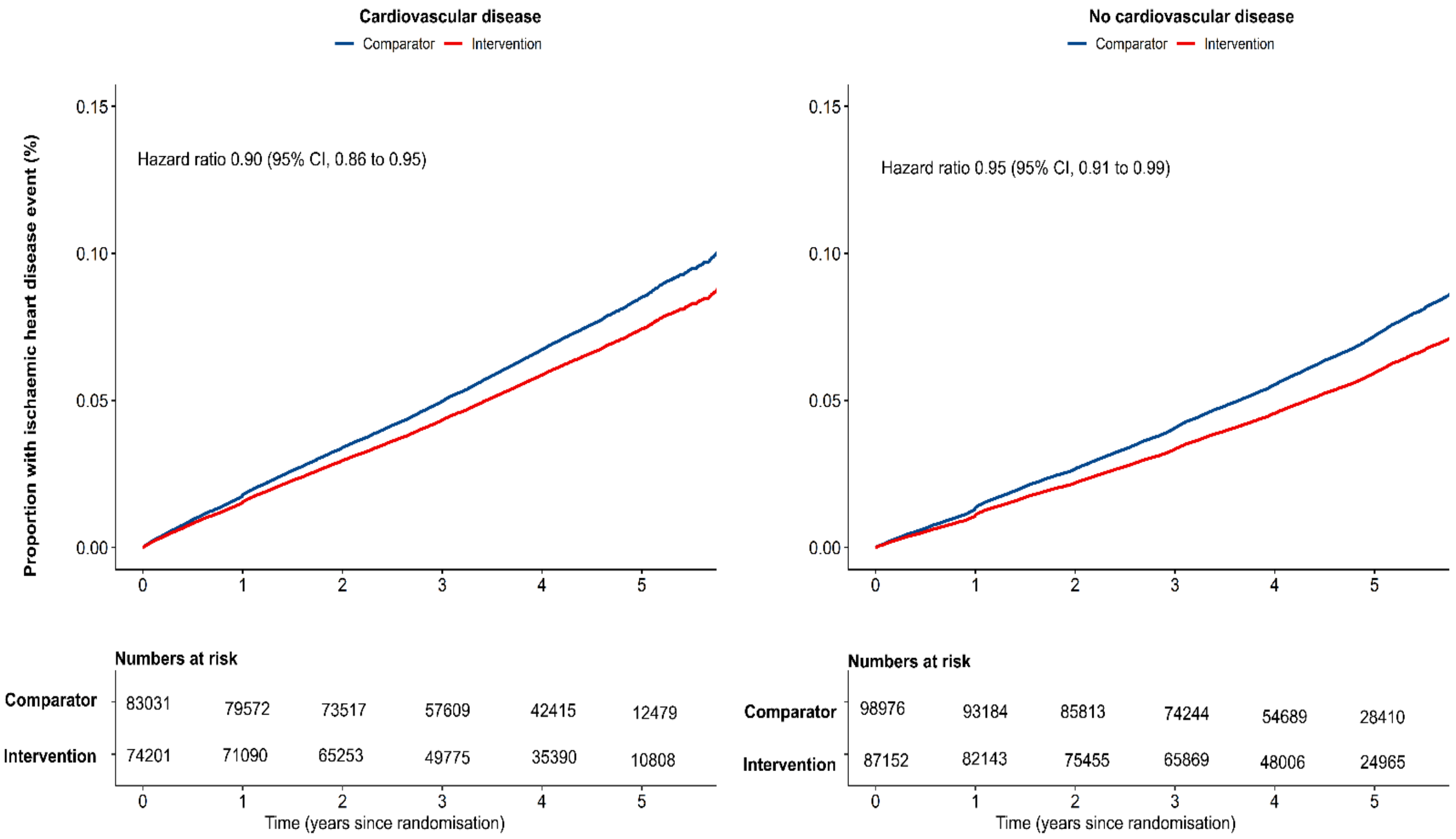

Figure S3. Kaplan–Meier rates of heart failure per 5 mmHg reduction in systolic blood pressure, stratified by treatment allocation and cardiovascular disease status at baseline.

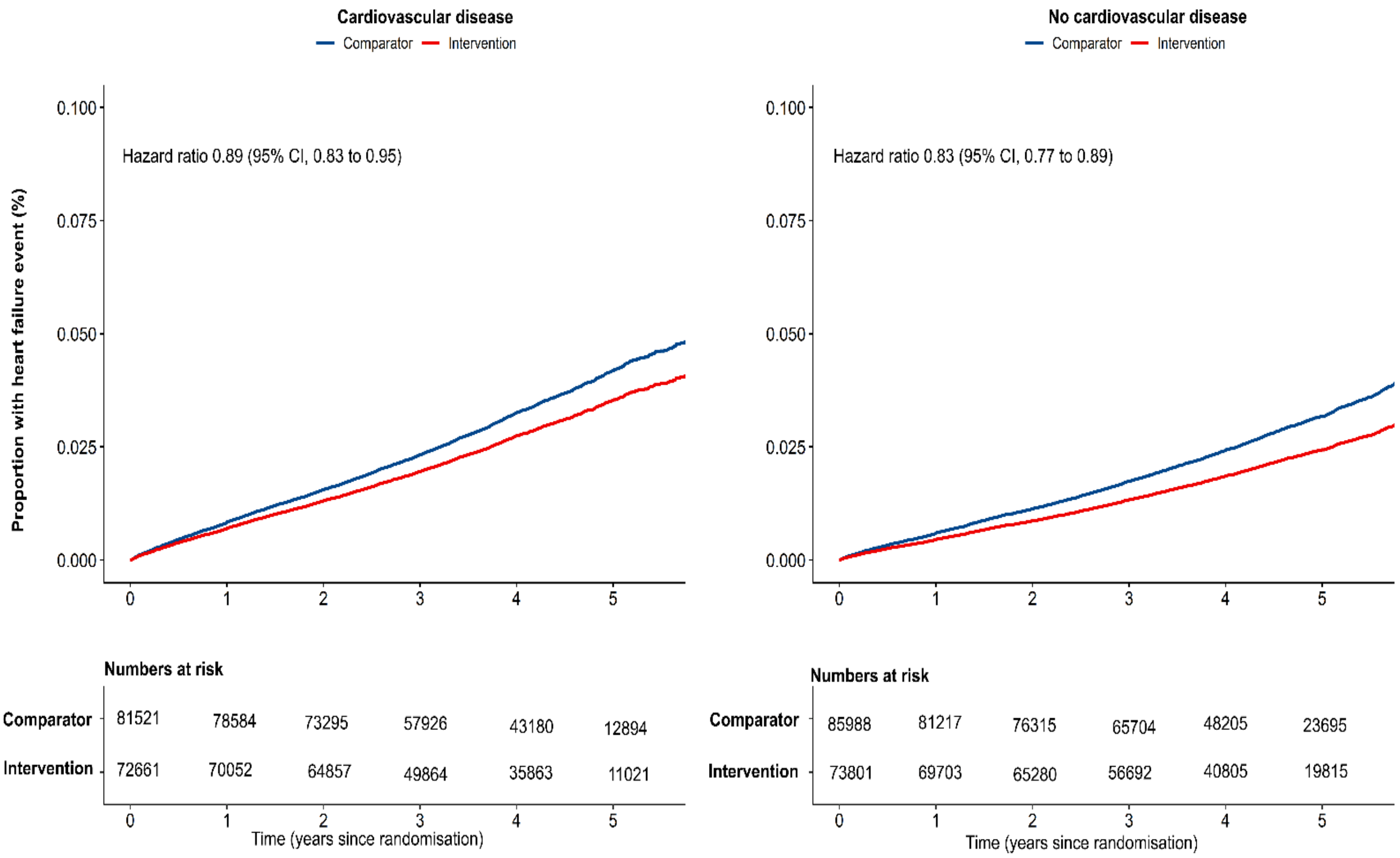

Figure S4. Kaplan–Meier rates of cardiovascular death per 5 mmHg reduction in systolic blood pressure, stratified by treatment allocation and cardiovascular disease status at baseline.

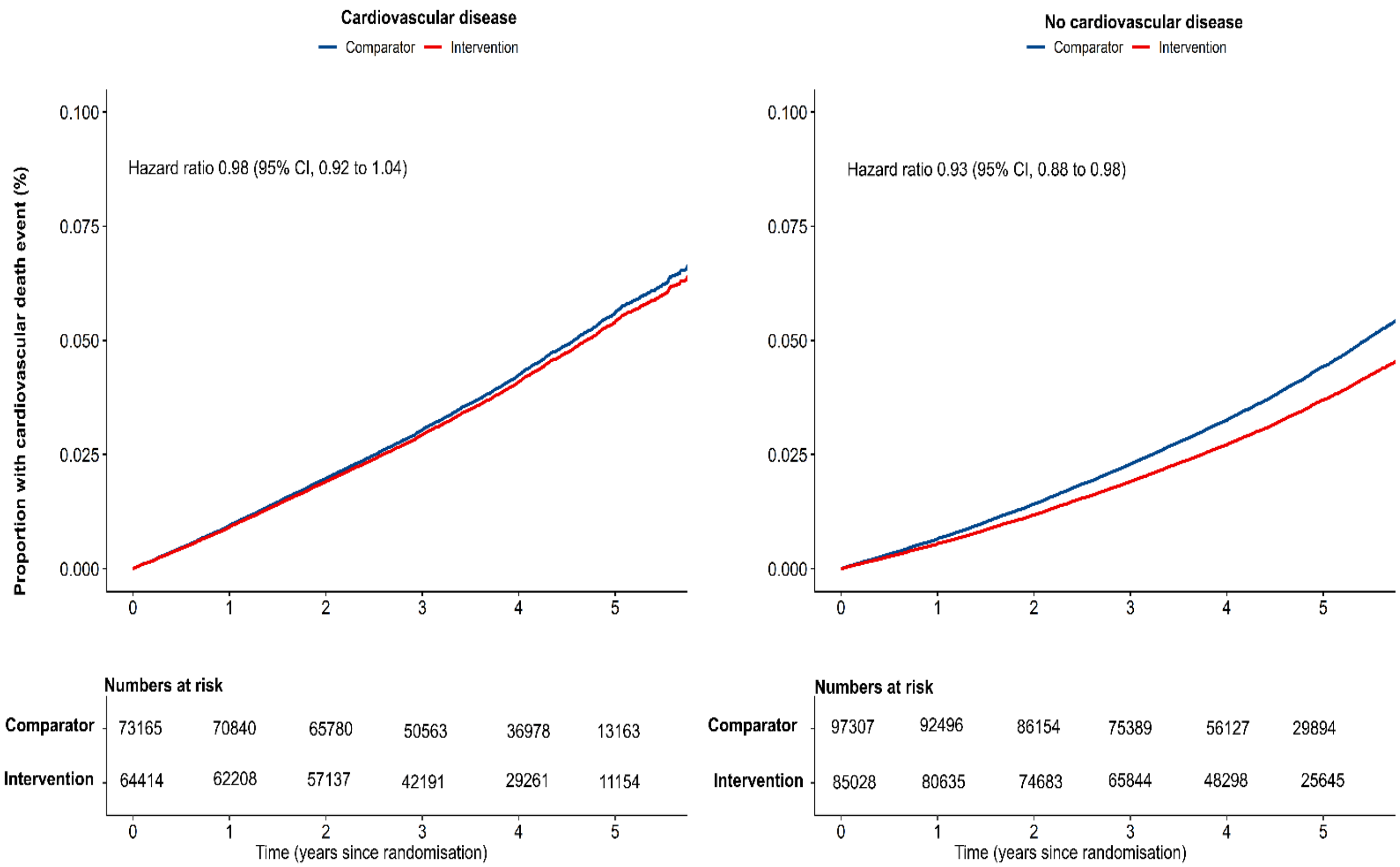

Figure S5. Kaplan–Meier rates of all-cause death per 5 mmHg reduction in systolic blood pressure, stratified by treatment allocation and cardiovascular disease status at baseline.

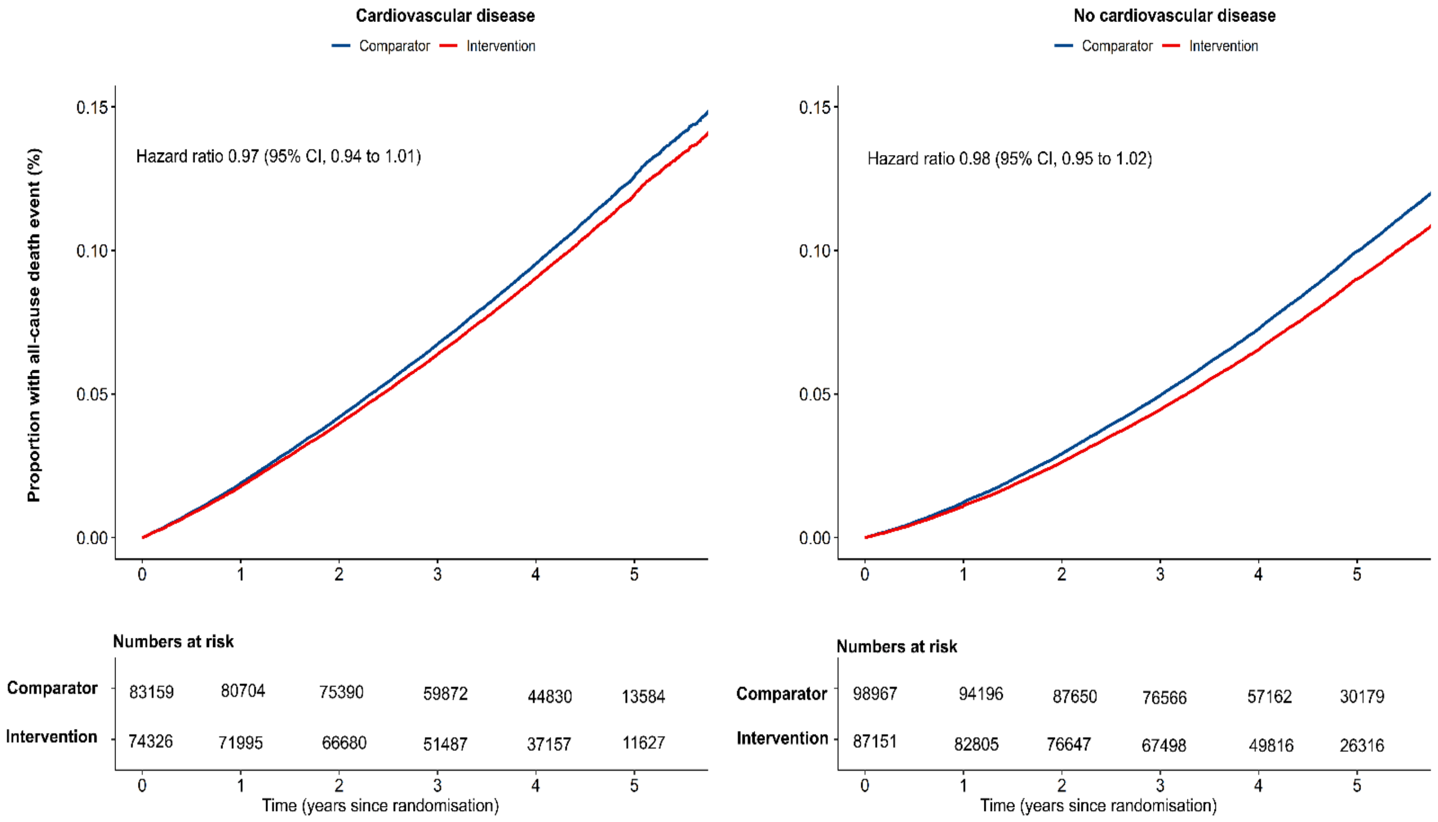

Figure S6. Effects of blood pressure-lowering treatment on primary and secondary outcomes by systolic blood pressure at baseline.

Forest plot shows the hazard ratios (HR) and 95% confidence intervals (CI) per 5 mmHg systolic blood pressure reduction, separately for each outcome; p for interaction-adj: Adjusted for multiple testing using Hommel's method; p for interaction-unadj: unadjusted for multiple testing.

Outcomes	Intervention Events	Intervention Total	Comparator Events	Comparator Total	HR	95% CI
**Major cardiovascular events**						
<120	830	7700	1216	8792	0.79	[0.72; 0.87]
120 to 129	1562	13571	2094	15786	0.91	[0.85; 0.98]
130 to 139	2536	21425	3273	24737	0.94	[0.89; 0.99]
140 to 149	3423	29295	4496	33889	0.93	[0.88; 0.98]
150 to 159	3130	27046	4264	31256	0.88	[0.83; 0.94]
160 to 169	2818	28443	3605	30265	0.87	[0.83; 0.92]
≥170	3979	33433	5080	36724	0.90	[0.86; 0.94]
p for interaction-adj=0.35						
p for interaction-unadj=0.05						
**Stroke**						
<120	192	7751	286	8842	0.79	[0.65; 0.97]
120 to 129	416	13633	546	15840	0.86	[0.74; 0.99]
130 to 139	759	21498	925	24831	0.92	[0.83; 1.02]
140 to 149	1062	29375	1326	33988	0.90	[0.82; 0.99]
150 to 159	932	27099	1350	31337	0.77	[0.65; 0.92]
160 to 169	1024	28503	1264	30357	0.86	[0.79; 0.94]
≥170	1618	33562	2068	36861	0.90	[0.84; 0.97]
p for interaction-adj=0.77						
p for interaction-unadj=0.11						
**Ischaemic heart disease**						
<120	406	7744	582	8832	0.83	[0.72; 0.95]
120 to 129	749	13627	995	15829	0.95	[0.86; 1.05]
130 to 139	1162	21476	1557	24821	0.96	[0.88; 1.04]
140 to 149	1553	29358	2155	33969	0.94	[0.87; 1.02]
150 to 159	1489	27082	2034	31310	0.91	[0.83; 0.99]
160 to 169	1226	28492	1606	30345	0.87	[0.80; 0.95]
≥170	1719	33556	2214	36853	0.94	[0.88; 1.01]
p for interaction-adj=1.00						
p for interaction-unadj=0.71						
**Heart failure**						
<120	196	7702	289	8783	0.78	[0.64; 0.96]
120 to 129	342	13369	432	15656	0.99	[0.85; 1.15]
130 to 139	462	20822	687	24295	0.85	[0.75; 0.97]
140 to 149	663	27944	912	32706	0.95	[0.85; 1.07]
150 to 159	587	25078	874	29498	0.88	[0.78; 1.00]
160 to 169	447	24371	698	26233	0.70	[0.61; 0.80]
≥170	549	27173	691	30305	0.89	[0.79; 1.00]
p for interaction-adj=0.42						
p for interaction-unadj=0.06						
**Cardiovascular death**						
<120	223	6462	275	7626	0.90	[0.72; 1.12]
120 to 129	324	11523	481	13743	0.89	[0.76; 1.05]
130 to 139	624	19034	745	22405	0.98	[0.87; 1.11]
140 to 149	842	27099	1026	31753	0.97	[0.86; 1.10]
150 to 159	778	25218	961	29449	1.00	[0.88; 1.14]
160 to 169	747	27295	956	29297	0.90	[0.82; 0.99]
≥170	1284	32803	1662	36163	0.95	[0.88; 1.02]
p for interaction-adj=0.49						
p for interaction-unadj=0.07						
**All-cause death**						
<120	646	7752	820	8846	0.88	[0.78; 1.00]
120 to 129	1054	13636	1327	15842	1.00	[0.91; 1.10]
130 to 139	1744	21513	2131	24839	0.98	[0.91; 1.05]
140 to 149	2351	29388	2817	33997	0.99	[0.92; 1.06]
150 to 159	2189	27106	2718	31336	0.95	[0.85; 1.07]
160 to 169	2000	28504	2420	30364	0.94	[0.88; 1.00]
≥170	3029	33560	3627	36853	0.97	[0.93; 1.02]
p for interaction-adj=1.00						
p for interaction-unadj=0.31						

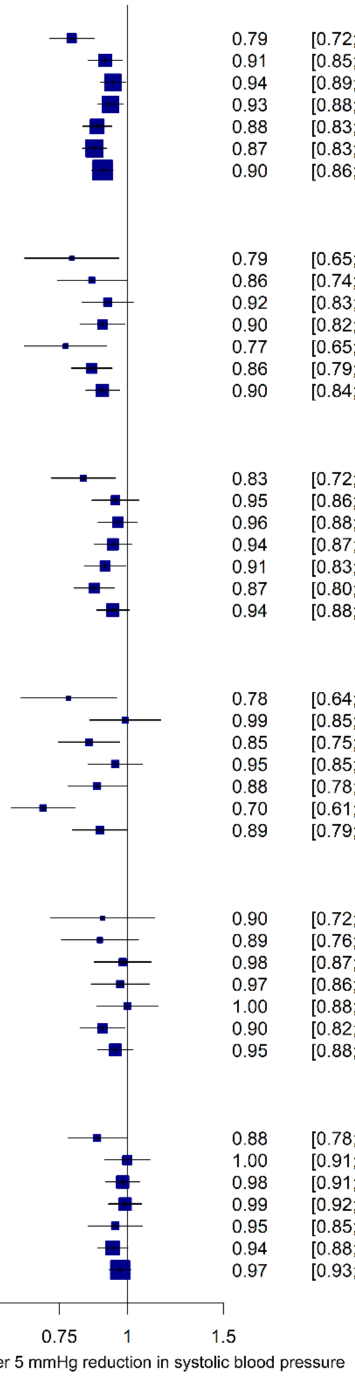

Figure S7. The unstandardised effects of blood pressure-lowering treatment on primary and secondary outcomes by systolic blood pressure at baseline.

Forest plot shows the hazard ratios (HR) and 95% confidence intervals (CI), separately for each outcome; p for interaction-adj: adjusted for multiple testing using Hommel's method; p for interaction-unadj: unadjusted for multiple testing.

	Intervention		Comparator			
Outcomes	Events	Total	Events	Total	HR	95% CI
**Major cardiovascular events**						
<120	830	7700	1216	8792	0.83	[0.76; 0.91]
120 to 129	1562	13571	2094	15786	0.92	[0.86; 0.98]
130 to 139	2536	21425	3273	24737	0.95	[0.90; 1.00]
140 to 149	3423	29295	4496	33889	0.96	[0.92; 1.00]
150 to 159	3130	27046	4264	31256	0.93	[0.89; 0.98]
160 to 169	2818	28443	3605	30265	0.90	[0.86; 0.95]
≥170	3979	33433	5080	36724	0.91	[0.87; 0.95]
p for interaction-adj=0.35						
p for interaction-unadj=0.05						
**Stroke**						
<120	192	7751	286	8842	0.80	[0.66; 0.96]
120 to 129	416	13633	546	15840	0.88	[0.78; 1.00]
130 to 139	759	21498	925	24831	0.94	[0.85; 1.03]
140 to 149	1062	29375	1326	33988	0.95	[0.87; 1.03]
150 to 159	932	27099	1350	31337	0.83	[0.76; 0.90]
160 to 169	1024	28503	1264	30357	0.89	[0.82; 0.97]
≥170	1618	33562	2068	36861	0.92	[0.86; 0.98]
p for interaction-adj=0.77						
p for interaction-unadj=0.11						
**Ischaemic heart disease**						
<120	406	7744	582	8832	0.87	[0.77; 0.99]
120 to 129	749	13627	995	15829	0.96	[0.87; 1.06]
130 to 139	1162	21476	1557	24821	0.97	[0.90; 1.05]
140 to 149	1553	29358	2155	33969	0.95	[0.89; 1.02]
150 to 159	1489	27082	2034	31310	0.96	[0.90; 1.03]
160 to 169	1226	28492	1606	30345	0.90	[0.84; 0.97]
≥170	1719	33556	2214	36853	0.92	[0.86; 0.98]
p for interaction-adj=1.00						
p for interaction-unadj=0.71						
**Heart failure**						
<120	196	7702	289	8783	0.87	[0.72; 1.05]
120 to 129	342	13369	432	15656	1.00	[0.87; 1.15]
130 to 139	462	20822	687	24295	0.86	[0.76; 0.97]
140 to 149	663	27944	912	32706	0.95	[0.86; 1.05]
150 to 159	587	25078	874	29498	0.89	[0.80; 0.99]
160 to 169	447	24371	698	26233	0.76	[0.67; 0.86]
≥170	549	27173	691	30305	0.91	[0.82; 1.01]
p for interaction-adj=0.42						
p for interaction-unadj=0.06						
**Cardiovascular death**						
<120	223	6462	275	7626	1.04	[0.87; 1.24]
120 to 129	324	11523	481	13743	0.82	[0.71; 0.94]
130 to 139	624	19034	745	22405	1.03	[0.93; 1.15]
140 to 149	842	27099	1026	31753	1.04	[0.95; 1.14]
150 to 159	778	25218	961	29449	1.02	[0.93; 1.12]
160 to 169	747	27295	956	29297	0.92	[0.84; 1.01]
≥170	1284	32803	1662	36163	0.94	[0.87; 1.01]
p for interaction-adj=0.49						
p for interaction-unadj=0.07						
**All-cause death**						
<120	646	7752	820	8846	1.00	[0.90; 1.11]
120 to 129	1054	13636	1327	15842	0.96	[0.88; 1.04]
130 to 139	1744	21513	2131	24839	1.00	[0.94; 1.07]
140 to 149	2351	29388	2817	33997	1.05	[0.99; 1.11]
150 to 159	2189	27106	2718	31336	1.01	[0.96; 1.07]
160 to 169	2000	28504	2420	30364	0.94	[0.89; 1.00]
≥170	3029	33560	3627	36853	0.97	[0.92; 1.02]
p for interaction-adj=1.00						
p for interaction-unadj=0.31						

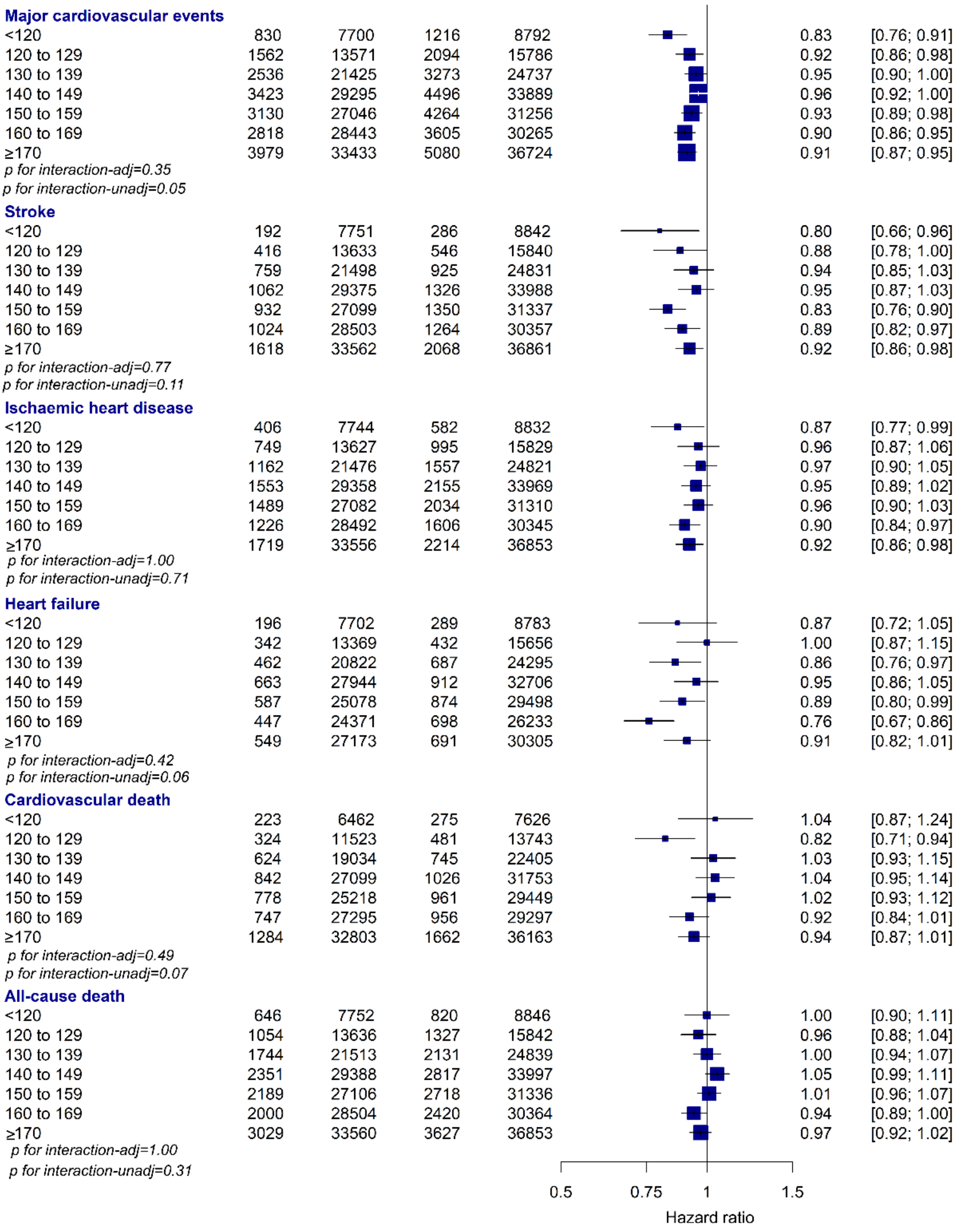

Figure S8. **Effects of blood pressure-lowering treatment on primary and secondary outcomes, by cardiovascular disease status at baseline, excluding drug classes comparison trials.**

Forest plot shows the hazard ratios (HR) and 95% confidence intervals (CI) per 5 mmHg reduction in systolic blood pressure, separately for each outcome; CVD: cardiovascular disease; p for interaction-adi: adjusted for multiple testing using Hommel’s method; p for interaction-unadi: unadjusted for multiple testing.

	Intervention		Comparator			
Outcomes	Events	Total	Events	Total	HR	95% CI
**Major cardiovascular events**						
Prior CVD	4757	37519	5358	36982	0.88	[0.85; 0.92]
No prior CVD	2636	28550	2981	27998	0.90	[0.85; 0.96]
**Overall**	**7393**	**66069**	**8339**	**64980**	**0.89**	**[0.86; 0.92]**
p for interaction-adj=1.00						
p for interaction-unadj=0.99						
**Stroke**						
Prior CVD	1979	37682	2235	37174	0.88	[0.83; 0.94]
No prior CVD	774	28621	1010	28090	0.81	[0.73; 0.90]
**Overall**	**2753**	**66303**	**3245**	**65264**	**0.86**	**[0.82; 0.91]**
p for interaction-adj=0.10						
p for interaction-unadj=0.05						
**Ischaemic heart disease**						
Prior CVD	1755	37615	2024	37100	0.86	[0.81; 0.92]
No prior CVD	926	28613	1021	28086	0.86	[0.76; 0.97]
**Overall**	**2681**	**66228**	**3045**	**65186**	**0.86**	**[0.81; 0.91]**
p for interaction-adj=1.00						
p for interaction-unadj=0.70						
**Heart failure**						
Prior CVD	485	25657	938	36149	0.87	[0.79; 0.96]
No prior CVD	817	36674	564	25461	0.89	[0.78; 1.01]
**Overall**	**1302**	**62331**	**1502**	**61610**	**0.88**	**[0.81; 0.95]**
p for interaction-adj=1.00						
p for interaction-unadj=0.97						
**Cardiovascular death**						
Prior CVD	1148	27808	1139	27196	1.03	[0.95; 1.12]
No prior CVD	798	26484	920	26415	0.92	[0.83; 1.02]
**Overall**	**1946**	**54292**	**2059**	**53611**	**0.99**	**[0.93; 1.06]**
p for interaction-adj=0.10						
p for interaction-unadj=0.05						
**All-cause death**						
Prior CVD	3245	37722	3379	37193	0.96	[0.92; 1.01]
No prior CVD	1969	28611	2108	28083	0.94	[0.88; 1.01]
**Overall**	**5214**	**66333**	**5487**	**65276**	**0.96**	**[0.92; 0.99]**
p for interaction-adj=1.00						
p for interaction-unadj=0.30						

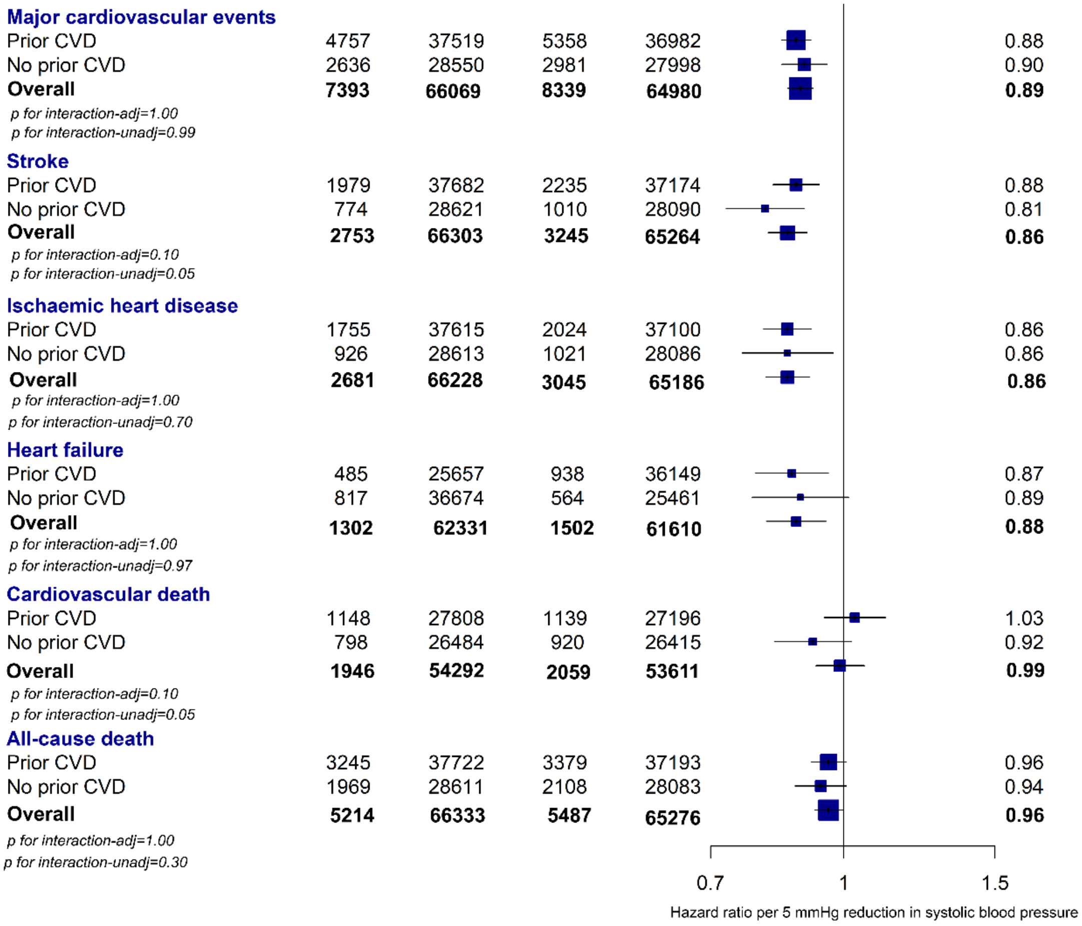

Figure S9. Effects of blood pressure-lowering treatment on primary and secondary outcomes, by cardiovascular disease status and systolic blood pressure at baseline, excluding drug classes comparison trials.

Forest plot shows the hazard ratios (HR) and 95% confidence intervals (CI) per 5 mmHg reduction in systolic blood pressure, separately for each outcome; p for interaction-adi: adjusted for multiple testing using Hommel’s method; p for interaction-unadi: unadjusted for multiple testing.

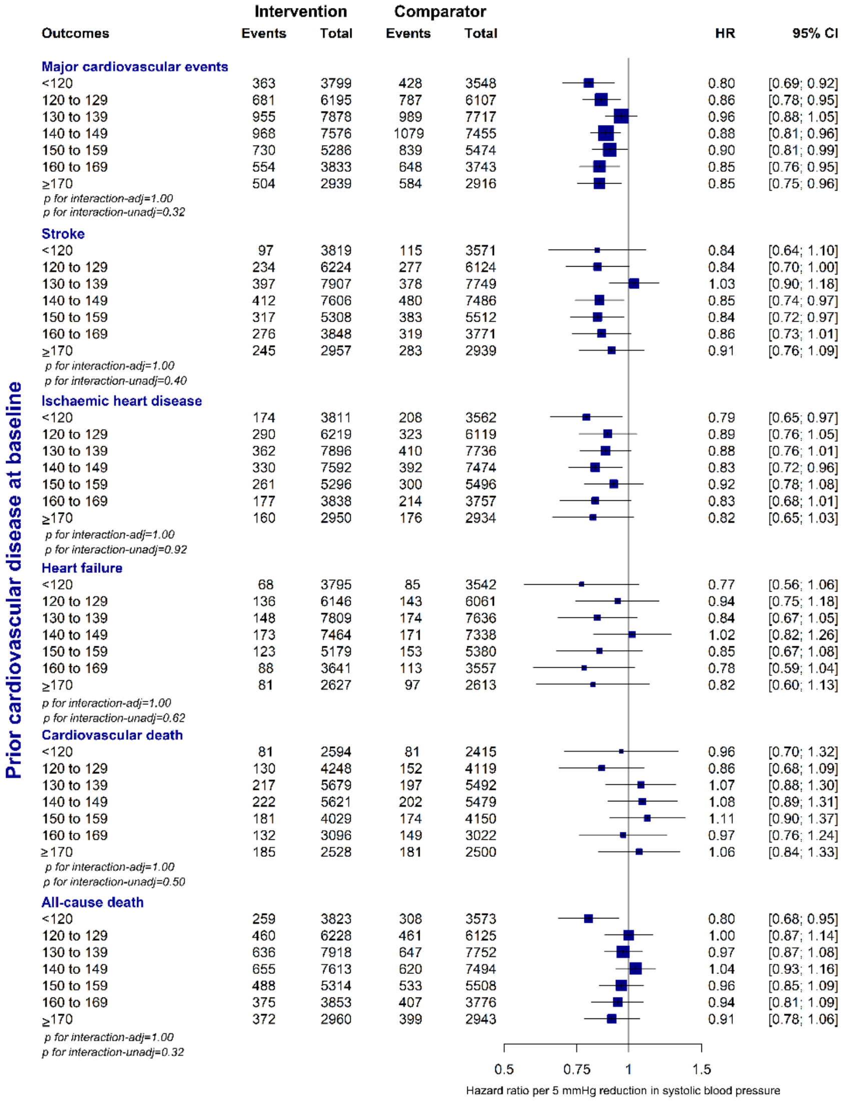

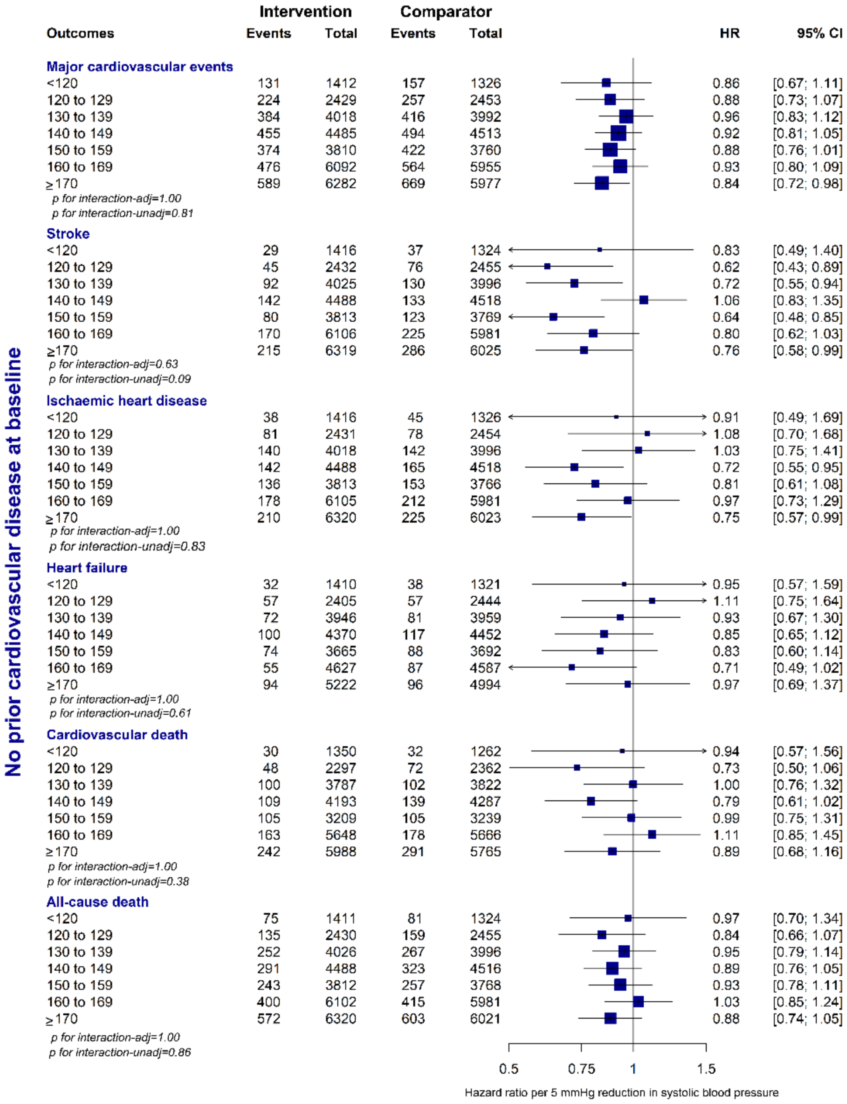

Figure S10. Absolute risk reduction for the effect of blood pressure-lowering treatment on primary and secondary outcomes, by cardiovascular disease status at baseline.

Absolute risk reduction (ARR) estimated using a Poisson regression model with identity link. The unit is absolute risk difference between treatment versus comparator groups and reflects mean of blood pressure (mmHg) reduction in BPLTTC; CVD: cardiovascular disease; CI: confidence interval; p for interaction-adi: adjusted for multiple testing using Hommel method; p for interaction-unadi: unadjusted for multiple testing.

	Intervention		Comparator			
Outcomes	Events	Total	Events	Total	ARR	95% CI
**Major cardiovascular events**						
Prior CVD	9356	73896	11718	82657	-0.016	[-0.019; -0.012]
No prior CVD	8931	87033	12319	98840	-0.022	[-0.025; -0.019]
**Overall**	**18287**	**160929**	**24037**	**181497**	**-0.019**	**[-0.021; -0.017]**
p for interaction-adj=0.02						
p for interaction-unadj=0.01						
**Stroke**						
Prior CVD	3404	74275	4167	83125	-0.004	[-0.006; -0.002]
No prior CVD	2601	87164	3600	98980	-0.007	[-0.008; -0.005]
**Overall**	**6005**	**161439**	**7767**	**182105**	**-0.005**	**[-0.007; -0.004]**
p for interaction-adj=0.22						
p for interaction-unadj=0.11						
**Ischaemic heart disease**						
Prior CVD	4042	74201	5185	83031	-0.008	[-0.011; -0.006]
No prior CVD	4265	87152	5960	98976	-0.011	[-0.013; -0.009]
**Overall**	**8307**	**161353**	**11145**	**182007**	**-0.010**	**[-0.011; -0.008]**
p for interaction-adj=0.11						
p for interaction-unadj=0.06						
**Heart failure**						
Prior CVD	1796	72661	2346	81521	-0.004	[-0.006; -0.002]
No prior CVD	1453	73801	2238	85988	-0.006	[-0.008; -0.005]
**Overall**	**3249**	**146462**	**4584**	**167509**	**-0.005**	**[-0.006; -0.004]**
p for interaction-adj=0.15						
p for interaction-unadj=0.07						
**Cardiovascular death**						
Prior CVD	2357	64414	2726	73165	-0.001	[-0.003; 0.001]
No prior CVD	2468	85028	3384	97307	-0.006	[-0.007; -0.004]
**Overall**	**4825**	**149442**	**6110**	**170472**	**-0.004**	**[-0.005; -0.002]**
p for interaction-adj<0.001						
p for interaction-unadj<0.001						
**All-cause death**						
Prior CVD	6646	74326	7905	83159	-0.006	[-0.009; -0.003]
No prior CVD	6375	87151	7969	98967	-0.007	[-0.010; -0.005]
**Overall**	**13021**	**161477**	**15874**	**182126**	**-0.007**	**[-0.009; -0.005]**
p for interaction-adj=0.91						
p for interaction-unadj=0.46						

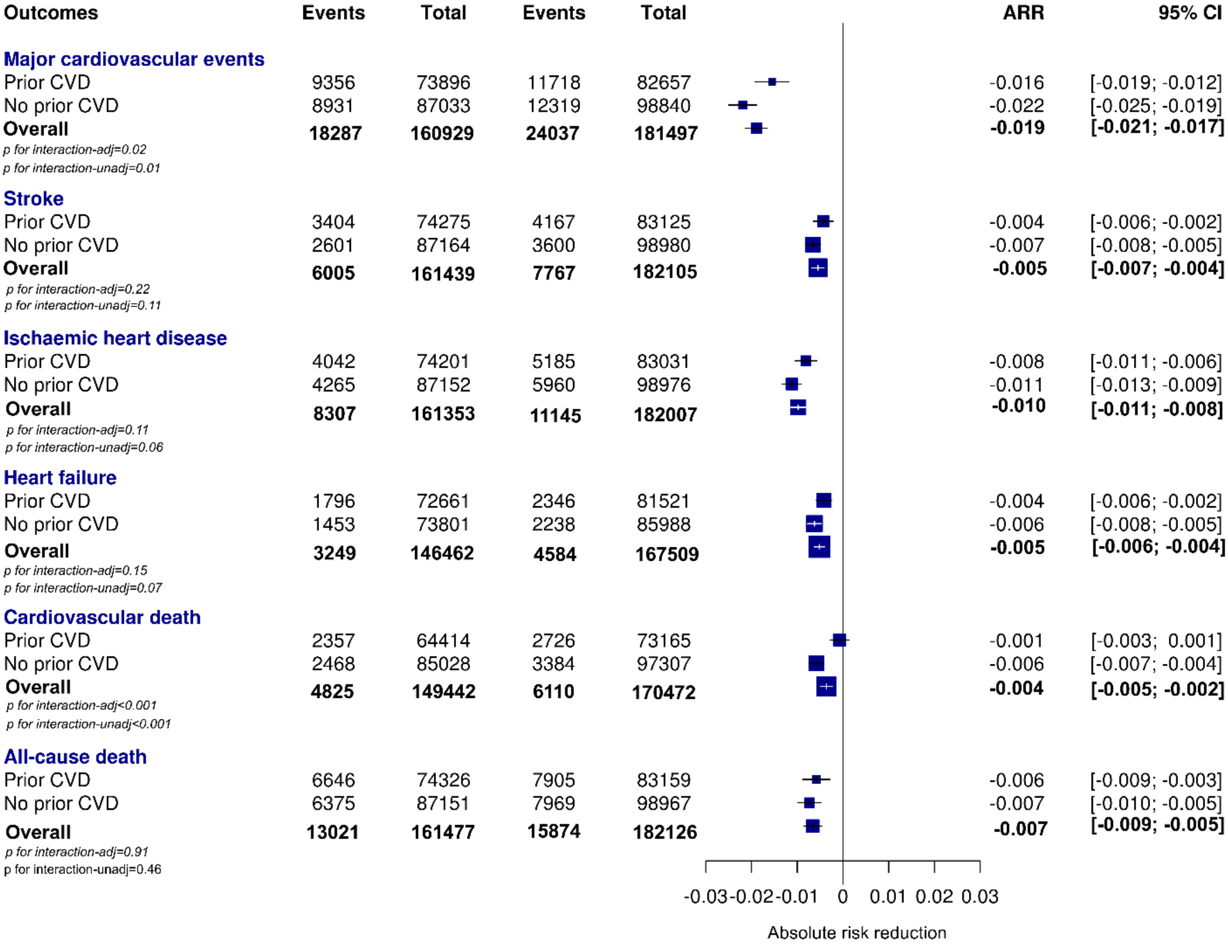

Figure S11. Absolute risk reduction for the effect of blood pressure-lowering treatment on primary and secondary outcomes, by cardiovascular disease status and systolic blood pressure at baseline.

Absolute risk reduction (ARR) estimated using a Poisson regression model with identity link. The unit is absolute risk difference between treatment versus comparator groups and reflects mean of blood pressure (mmHg) reduction in BPLTTC; CVD: cardiovascular disease; CI: confidence interval; p for interaction-adi: adjusted for multiple testing using Hommel method; p for interaction-unadi: unadjusted for multiple testing.

Prior cardiovascular disease at baseline

Outcomes	Intervention Events	Intervention Total	Comparator Events	Comparator Total	ARR	95% CI
**Major cardiovascular events**						
<120	562	5506	821	6211	-0.030	[-0.043; -0.017]
120 to 129	1020	9029	1306	10234	-0.013	[-0.023; -0.003]
130 to 139	1555	12887	1835	14424	-0.008	[-0.016; 0.001]
140 to 149	1852	15043	2321	16938	-0.014	[-0.022; -0.006]
150 to 159	1606	12306	2091	14303	-0.017	[-0.026; -0.008]
160 to 169	1247	9626	1555	10424	-0.021	[-0.031; -0.010]
≥170	1507	9454	1781	10063	-0.018	[-0.029; -0.006]
p for interaction-adj=0.91						
p for interaction-unadj=0.13						
**Stroke**						
<120	142	5553	209	6263	-0.008	[-0.014; -0.002]
120 to 129	317	9088	394	10286	-0.003	[-0.008; 0.002]
130 to 139	546	12952	591	14514	0.001	[-0.003; 0.006]
140 to 149	670	15117	836	17031	-0.005	[-0.009; 0.000]
150 to 159	562	12353	801	14372	-0.010	[-0.016; -0.005]
160 to 169	530	9656	614	10472	-0.004	[-0.011; 0.002]
≥170	634	9511	721	10126	-0.004	[-0.012; 0.003]
p for interaction-adj=0.49						
p for interaction-unadj=0.07						
**Ischaemic heart disease**						
<120	276	5546	402	6251	-0.015	[-0.024; -0.006]
120 to 129	478	9083	613	10276	-0.007	[-0.014; 0.000]
130 to 139	668	12937	844	14502	-0.006	[-0.012; -0.001]
140 to 149	773	15100	1031	17014	-0.009	[-0.015; -0.004]
150 to 159	718	12338	909	14347	-0.006	[-0.012; 0.000]
160 to 169	491	9647	626	10460	-0.009	[-0.016; -0.003]
≥170	636	9505	757	10121	-0.008	[-0.015; 0.000]
p for interaction-adj=1.00						
p for interaction-unadj=0.67						
**Heart failure**						
<120	138	5531	204	6233	-0.008	[-0.014; -0.001]
120 to 129	217	9008	269	10218	-0.001	[-0.006; 0.003]
130 to 139	292	12838	382	14392	-0.004	[-0.008; 0.000]
140 to 149	364	14941	465	16842	-0.004	[-0.007; 0.000]
150 to 159	304	12149	439	14177	-0.006	[-0.010; -0.002]
160 to 169	226	9331	304	10124	-0.007	[-0.011; -0.002]
≥170	253	8820	281	9475	-0.002	[-0.007; 0.004]
p for interaction-adj=1.00						
p for interaction-unadj=0.55						
**Cardiovascular death**						
<120	146	4330	182	5109	-0.002	[-0.009; 0.006]
120 to 129	210	7113	278	8282	-0.004	[-0.010; 0.002]
130 to 139	365	10726	386	12260	0.003	[-0.002; 0.007]
140 to 149	461	13136	497	15028	0.002	[-0.002; 0.006]
150 to 159	386	11076	448	13013	0.000	[-0.004; 0.005]
160 to 169	304	8905	390	9727	-0.006	[-0.012; -0.001]
≥170	484	9083	541	9688	-0.003	[-0.009; 0.004]
p for interaction-adj=1.00						
p for interaction-unadj=0.17						
**All-cause death**						
<120	462	5559	603	6267	-0.014	[-0.024; -0.003]
120 to 129	706	9093	854	10288	-0.005	[-0.013; 0.003]
130 to 139	1078	12965	1272	14520	-0.005	[-0.012; 0.002]
140 to 149	1303	15129	1505	17043	-0.002	[-0.009; 0.004]
150 to 159	1155	12361	1411	14371	-0.005	[-0.013; 0.002]
160 to 169	863	9660	1046	10481	-0.011	[-0.019; -0.002]
≥170	1076	9514	1205	10128	-0.006	[-0.015; 0.004]
p for interaction-adj=1.00						
p for interaction-unadj=0.62						

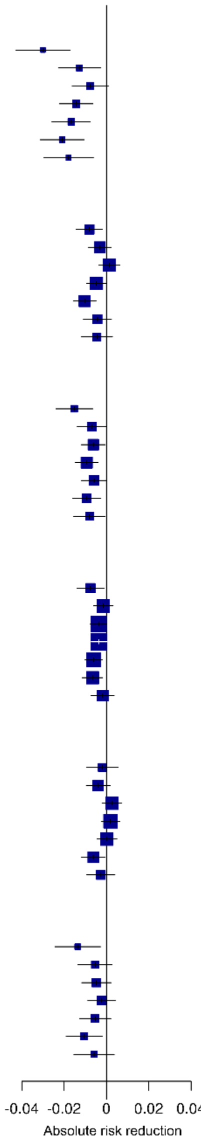

No prior cardiovascular disease at baseline

Outcomes	Intervention Events	Intervention Total	Comparator Events	Comparator Total	ARR	95% CI
**Major cardiovascular events**						
<120	268	2193	395	2581	-0.029	[-0.050; -0.008]
120 to 129	542	4542	788	5552	-0.022	[-0.036; -0.008]
130 to 139	981	8538	1438	10313	-0.024	[-0.035; -0.014]
140 to 149	1571	14249	2175	16947	-0.018	[-0.026; -0.010]
150 to 159	1524	14737	2173	16948	-0.025	[-0.033; -0.018]
160 to 169	1571	18773	2049	19811	-0.020	[-0.026; -0.014]
≥170	2470	23933	3295	26614	-0.020	[-0.026; -0.014]
p for interaction-adj=1.00						
p for interaction-unadj=0.82						
**Stroke**						
<120	50	2197	77	2579	-0.007	[-0.016; 0.002]
120 to 129	99	4545	152	5554	-0.006	[-0.012; 0.001]
130 to 139	213	8546	334	10317	-0.008	[-0.012; -0.003]
140 to 149	392	14255	490	16953	-0.001	[-0.005; 0.002]
150 to 159	370	14743	549	16960	-0.007	[-0.011; -0.004]
160 to 169	494	18803	649	19855	-0.006	[-0.010; -0.003]
≥170	982	24005	1347	26688	-0.010	[-0.013; -0.006]
p for interaction-adj=0.77						
p for interaction-unadj=0.11						
**Ischaemic heart disease**						
<120	130	2197	180	2581	-0.011	[-0.025; 0.004]
120 to 129	271	4544	382	5553	-0.009	[-0.019; 0.001]
130 to 139	494	8539	713	10319	-0.011	[-0.018; -0.003]
140 to 149	780	14255	1124	16951	-0.012	[-0.017; -0.006]
150 to 159	771	14741	1125	16958	-0.014	[-0.019; -0.009]
160 to 169	735	18801	980	19855	-0.010	[-0.014; -0.006]
≥170	1083	24005	1454	26685	-0.009	[-0.013; -0.006]
p for interaction-adj=1.00						
p for interaction-unadj=0.89						
**Heart failure**						
<120	58	2170	85	2550	-0.005	[-0.015; 0.005]
120 to 129	125	4361	163	5438	-0.001	[-0.008; 0.006]
130 to 139	170	7984	305	9903	-0.010	[-0.014; -0.005]
140 to 149	299	13000	447	15860	-0.005	[-0.009; -0.001]
150 to 159	283	12926	435	15316	-0.007	[-0.011; -0.003]
160 to 169	221	15001	394	16082	-0.010	[-0.013; -0.007]
≥170	296	18310	409	20783	-0.003	[-0.006; 0.000]
p for interaction-adj=0.21						
p for interaction-unadj=0.03						
**Cardiovascular death**						
<120	77	2131	93	2517	-0.001	[-0.012; 0.010]
120 to 129	114	4410	203	5461	-0.011	[-0.018; -0.005]
130 to 139	259	8308	359	10145	-0.004	[-0.010; 0.001]
140 to 149	381	13960	529	16721	-0.004	[-0.008; -0.001]
150 to 159	392	14139	513	16431	-0.004	[-0.007; 0.000]
160 to 169	443	18346	566	19540	-0.005	[-0.008; -0.001]
≥170	800	23674	1119	26428	-0.009	[-0.012; -0.005]
p for interaction-adj=1.00						
p for interaction-unadj=0.21						
**All-cause death**						
<120	184	2192	217	2579	0.001	[-0.015; 0.018]
120 to 129	348	4543	473	5554	-0.008	[-0.019; 0.003]
130 to 139	666	8548	859	10319	-0.005	[-0.014; 0.003]
140 to 149	1048	14256	1312	16950	-0.004	[-0.010; 0.002]
150 to 159	1034	14742	1307	16960	-0.007	[-0.013; -0.001]
160 to 169	1137	18800	1374	19853	-0.008	[-0.014; -0.003]
≥170	1953	24000	2420	26678	-0.009	[-0.014; -0.004]
p for interaction-adj=1.00						
p for interaction-unadj=0.79						

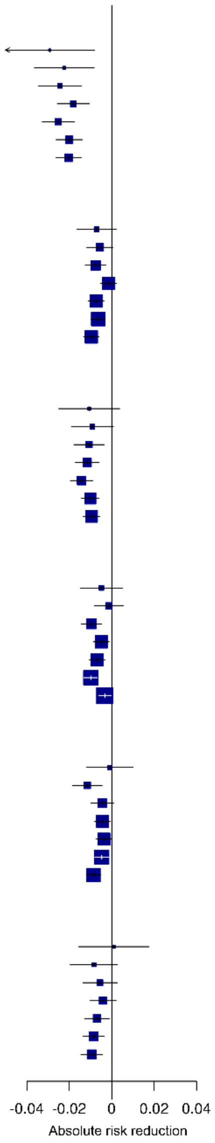

Table S12. Sensitivity analysis for the effect of blood pressure-lowering treatment on primary and secondary outcomes, stratified by cardiovascular disease status and systolic blood pressure at baseline, excluding the trials with risk of bias.

Forest plot shows the hazard ratios (HR) and 95% confidence intervals (CI) per 5 mmHg systolic blood pressure reduction, separately for each outcome; p for interaction-adi: adjusted for multiple testing using Hommel method; p for interaction-unadi: unadjusted for multiple testing.

Prior cardiovascular disease at baseline

	Intervention		Comparator			
Outcomes	Events	Total	Events	Total	HR	95% CI
**Major cardiovascular events**						
<120	562	5506	821	6211	0.77	[0.68; 0.87]
120 to 129	1019	9026	1306	10228	0.89	[0.81; 0.98]
130 to 139	1554	12872	1834	14414	0.99	[0.92; 1.06]
140 to 149	1845	15017	2318	16918	0.89	[0.83; 0.96]
150 to 159	1596	12220	2080	14212	0.90	[0.83; 0.97]
160 to 169	1236	9556	1541	10347	0.84	[0.77; 0.92]
≥170	1469	9317	1735	9919	0.89	[0.82; 0.97]
p for interaction-adj=0.28						
p for interaction-unadj=0.04						
**Stroke**						
<120	142	5553	209	6263	0.85	[0.66; 1.09]
120 to 129	316	9085	394	10280	0.88	[0.75; 1.04]
130 to 139	546	12937	590	14504	1.03	[0.90; 1.17]
140 to 149	667	15091	834	17011	0.87	[0.77; 0.98]
150 to 159	557	12267	794	14281	0.81	[0.71; 0.92]
160 to 169	526	9586	606	10395	0.84	[0.73; 0.96]
≥170	619	9374	698	9982	0.94	[0.83; 1.07]
p for interaction-adj=0.28						
p for interaction-unadj=0.04						
**Ischaemic heart disease**						
<120	276	5546	402	6251	0.78	[0.66; 0.93]
120 to 129	478	9080	613	10270	0.93	[0.81; 1.06]
130 to 139	667	12922	844	14492	0.97	[0.87; 1.09]
140 to 149	769	15074	1030	16994	0.85	[0.76; 0.95]
150 to 159	714	12252	906	14256	0.90	[0.79; 1.02]
160 to 169	486	9577	621	10383	0.84	[0.72; 0.97]
≥170	623	9368	744	9977	0.96	[0.83; 1.12]
p for interaction-adj=1.00						
p for interaction-unadj=0.35						
**Heart failure**						
<120	138	5531	204	6233	0.75	[0.58; 0.97]
120 to 129	217	9008	269	10218	0.97	[0.79; 1.19]
130 to 139	292	12838	382	14392	0.91	[0.77; 1.08]
140 to 149	364	14941	465	16842	0.95	[0.81; 1.11]
150 to 159	303	12093	438	14113	0.88	[0.73; 1.06]
160 to 169	225	9289	302	10085	0.78	[0.63; 0.97]
≥170	250	8757	277	9404	0.89	[0.73; 1.09]
p for interaction-adj=1.00						
p for interaction-unadj=0.81						
**Cardiovascular death**						
<120	146	4330	182	5109	0.86	[0.65; 1.14]
120 to 129	210	7110	278	8276	0.91	[0.74; 1.12]
130 to 139	365	10711	386	12250	1.02	[0.87; 1.20]
140 to 149	459	13110	497	15008	1.01	[0.86; 1.18]
150 to 159	384	11046	445	12986	1.04	[0.89; 1.21]
160 to 169	301	8863	384	9675	0.83	[0.70; 0.98]
≥170	460	8950	513	9562	1.02	[0.88; 1.18]
p for interaction-adj=1.00						
p for interaction-unadj=0.18						
**All-cause death**						
<120	462	5559	603	6267	0.81	[0.69; 0.95]
120 to 129	706	9090	854	10282	1.01	[0.89; 1.14]
130 to 139	1078	12950	1272	14510	0.99	[0.90; 1.09]
140 to 149	1300	15103	1504	17023	1.00	[0.91; 1.10]
150 to 159	1150	12275	1406	14280	0.98	[0.88; 1.09]
160 to 169	854	9590	1035	10404	0.91	[0.82; 1.01]
≥170	1035	9376	1164	9984	0.98	[0.89; 1.08]
p for interaction-adj=1.00						
p for interaction-unadj=0.71						

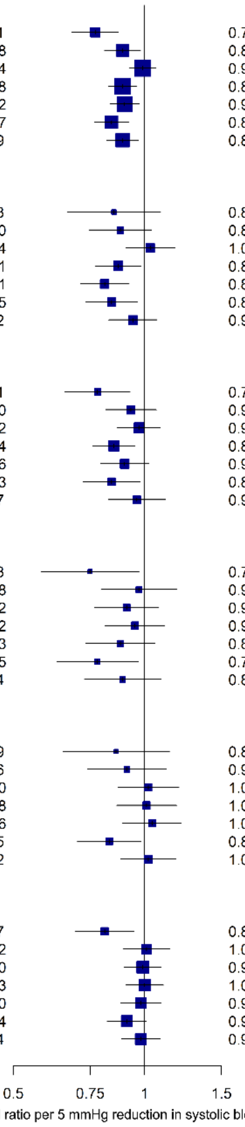

No prior cardiovascular disease at baseline

	Intervention		Comparator			
Outcomes	Events	Total	Events	Total	HR	95% CI
**Major cardiovascular events**						
<120	268	2172	392	2555	0.84	[0.71; 0.99]
120 to 129	535	4387	785	5447	0.94	[0.84; 1.06]
130 to 139	960	8089	1422	9971	0.89	[0.81; 0.97]
140 to 149	1532	13386	2137	16170	0.95	[0.88; 1.03]
150 to 159	1480	13370	2108	15726	0.87	[0.80; 0.94]
160 to 169	1492	17445	1961	18359	0.88	[0.82; 0.95]
≥170	2273	21825	3066	24256	0.89	[0.84; 0.94]
p for interaction-adj=1.00						
p for interaction-unadj=0.63						
**Stroke**						
<120	50	2176	76	2553	0.77	[0.53; 1.11]
120 to 129	95	4390	150	5449	0.79	[0.60; 1.05]
130 to 139	203	8097	323	9975	0.73	[0.60; 0.89]
140 to 149	379	13392	475	16176	0.96	[0.81; 1.13]
150 to 159	351	13376	519	15738	0.73	[0.61; 0.88]
160 to 169	463	17475	608	18403	0.85	[0.75; 0.96]
≥170	897	21897	1243	24330	0.88	[0.80; 0.96]
p for interaction-adj=1.00						
p for interaction-unadj=0.35						
**Ischaemic heart disease**						
<120	130	2176	179	2555	0.97	[0.77; 1.22]
120 to 129	269	4389	381	5448	1.00	[0.85; 1.18]
130 to 139	484	8090	707	9977	0.97	[0.86; 1.09]
140 to 149	754	13392	1104	16174	0.99	[0.89; 1.11]
150 to 159	750	13374	1094	15736	0.91	[0.81; 1.02]
160 to 169	695	17473	938	18403	0.88	[0.79; 0.98]
≥170	1002	21897	1373	24327	0.91	[0.83; 1.00]
p for interaction-adj=1.00						
p for interaction-unadj=0.84						
**Heart failure**						
<120	58	2170	85	2550	0.83	[0.59; 1.16]
120 to 129	125	4361	163	5437	1.02	[0.80; 1.30]
130 to 139	170	7984	305	9903	0.75	[0.62; 0.91]
140 to 149	299	12999	447	15857	0.93	[0.78; 1.10]
150 to 159	283	12619	431	15025	0.90	[0.75; 1.07]
160 to 169	217	14738	390	15807	0.61	[0.50; 0.74]
≥170	282	17880	395	20360	0.83	[0.70; 0.99]
p for interaction-adj=0.21						
p for interaction-unadj=0.03						
**Cardiovascular death**						
<120	77	2110	92	2491	0.96	[0.66; 1.39]
120 to 129	111	4255	203	5356	0.84	[0.64; 1.11]
130 to 139	252	7859	355	9803	0.93	[0.77; 1.12]
140 to 149	370	13097	517	15944	0.92	[0.77; 1.10]
150 to 159	378	13073	509	15495	0.94	[0.77; 1.14]
160 to 169	428	17116	545	18183	0.96	[0.85; 1.09]
≥170	719	21615	1027	24118	0.90	[0.81; 0.99]
p for interaction-adj=1.00						
p for interaction-unadj=0.34						
**All-cause death**						
<120	184	2171	216	2553	1.04	[0.84; 1.29]
120 to 129	340	4388	470	5449	0.98	[0.91; 1.06]
130 to 139	655	8099	852	9977	0.98	[0.88; 1.09]
140 to 149	1019	13393	1292	16173	0.96	[0.87; 1.06]
150 to 159	1007	13375	1282	15738	0.93	[0.83; 1.04]
160 to 169	1098	17472	1323	18401	0.96	[0.88; 1.04]
≥170	1794	21892	2244	24320	0.96	[0.90; 1.02]
p for interaction-adj=1.00						
p for interaction-unadj=0.56						

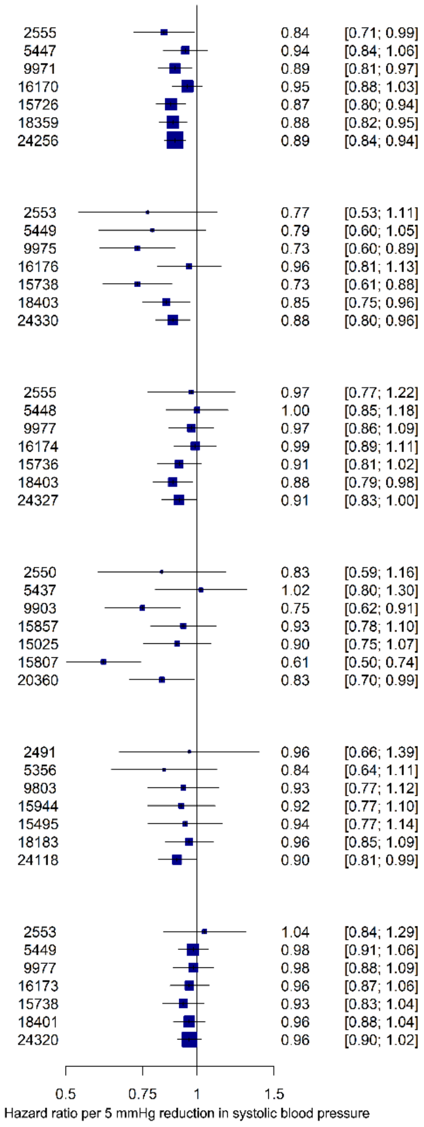

Figure S13. Funnel plot for assessment of publication (acquisition) bias on the effect of blood pressure reduction and risk of major cardiovascular event.

Linear regression test of funnel plot asymmetry: T-statistics = -1.18, df = 46, p-value = 0.24, bias coefficient -0.42, standard error 0.35.

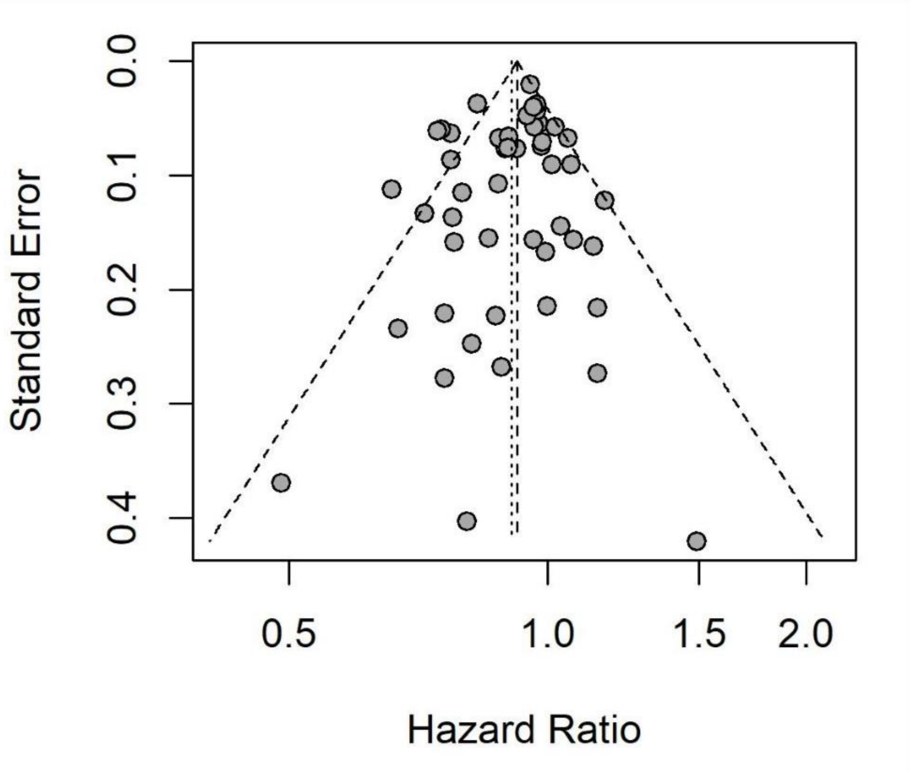

References

1 Thomopoulos C, Parati G, Zanchetti A. Effects of blood pressure lowering on outcome incidence in hypertension.1. Overview, meta-analyses, and meta-regression analyses of randomized trials. Journal of Hypertension. 2014; **32**: 2285–95.

2 Law MR, Morris JK, Wald NJ. Use of blood pressure lowering drugs in the prevention of cardiovascular disease: meta-analysis of 147 randomised trials in the context of expectations from prospective epidemiological studies. *BMJ* 2009; **338**: b1665–b1665.

3 Ettehad D, Emdin CA, Kiran A, *et al.* Blood pressure lowering for prevention of cardiovascular disease and death: a systematic review and meta-analysis. *Lancet* 2016; **387**: 957–67.

4 Emdin CA, Rahimi K, Neal B, Callender T, Perkovic V, Patel A. Blood pressure lowering in type 2 diabetes : A systematic review and meta-analysis. JAMA - Journal of the American Medical Association. 2015; **313**: 603–15.

5 Canoy D, Copland E, Nazarzadeh M, *et al.* Effect of antihypertensive drug treatment on long-term blood pressure reduction: An individual patient-level data meta-analysis of 352,744 Participants from 51 large-scale randomised clinical trials. *medRxiv* 2021; : 2021.02.19.21252066.

6 Appel LJ, Wright JT, Greene T. Intensive blood-pressure control in hypertensive chronic kidney disease. *New England Journal of Medicine* 2010; **363**: 2565–6.

7 Schrier RW, Estacio RO, Jeffers B. Appropriate Blood Pressure Control in NIDDM (ABCD) Trial. *Diabetologia* 1996; **39**: 1646–54.

8 Cushman WC, Evans GW, Byington RP, *et al.* Effects of intensive blood-pressure control in type 2 diabetes mellitus. *New England Journal of Medicine* 2010; **362**: 1575–85.

9 Yusuf S, Healey JS, Pogue J, *et al.* Irbesartan in patients with atrial fibrillation. *New England Journal of Medicine* 2011; **364**: 928–38.

10 Patel A. Effects of a fixed combination of perindopril and indapamide on macrovascular and microvascular outcomes in patients with type 2 diabetes mellitus (the ADVANCE trial): a randomised controlled trial. *Lancet* 2007; **370**: 829–40.

11 Group TAO and C for the ACR, Coordinators TAO and, Antihypertensive T, Treatment L. Major Outcomes in High-Risk Hypertensive Patients Randomized to Angiotensin-Converting Enzyme Inhibitor or Calcium Channel Blocker vs Diuretic. *JAMA: The Journal of the American Medical Association* 2002; **288**: 2981–97.

12 Doyle AE. The Australian National blood pressure study. *Trends in Pharmacological Sciences* 1981; **2**: 293–6.

13 Wing LMH, Reid CM, Ryan P, *et al.* A comparison of outcomes with angiotensin-converting-enzyme inhibitors and diuretics for hypertension in the elderly. *New England Journal of Medicine* 2003; **348**: 583–92.

14 Dahlöf B, Sever PS, Poulter NR, *et al.* Prevention of cardiovascular events with an antihypertensive regimen of amlodipine adding perindopril as required versus atenolol adding bendroflumethiazide as required, in the Anglo-Scandinavian Cardiac Outcomes Trial-Blood Pressure Lowering Arm (ASCOT-BPLA): a multicentre randomised controlled trial.

Lancet 2005; **366**: 895–906.

15 Ruggenenti P, Fassi A, Ilieva AP, *et al.* Preventing microalbuminuria in type 2 diabetes. *New England Journal of Medicine* 2004; **351**: 1941–51.

16 Park S, Yan P, Cerezo C, Jeffers BW. Effect of visit-to-visit blood pressure variability on cardiovascular events in patients with coronary artery disease and well-controlled blood pressure. *Journal of the American Society of Hypertension* 2016; **10**: 799–810.

17 Hansson L, Lindholm LH, Niskanen L, *et al.* Effect of angiotensin-converting-enzyme inhibition compared with conventional therapy on cardiovascular morbidity and mortality in hypertension: the Captopril Prevention Project (CAPPP) randomised trial. *Lancet* 1999; **353**: 611–6.

18 Verdecchia P, Staessen JA, Angeli F, *et al.* Usual versus tight control of systolic blood pressure in non-diabetic patients with hypertension (Cardio-Sis): an open-label randomised trial. *Lancet* 2009; **374**: 525–33.

19 Nakao K, Hirata M, Oba K, *et al.* Role of diabetes and obesity in outcomes of the candesartan antihypertensive survival evaluation in Japan (CASE-J) trial. *Hypertension Research* 2010; **33**: 600–6.

20 Ogihara T, Saruta T, Rakugi H, *et al.* Combinations of olmesartan and a calciumchannel blocker or a diuretic inelderly hypertensive patients: A randomized, controlled trial. *Journal of Hypertension* 2014; **32**: 2054–63.

21 Black HR, Elliott WJ, Grandits G, *et al.* Principal Results of the Controlled Onset Verapamil Investigation of Cardiovascular End Points (CONVINCE) Trial. *Journal of the American Medical Association* 2003; **289**: 2073–82.

22 Matsuzaki M, Ogihara T, Umemoto S, *et al.* Prevention of cardiovascular events with calcium channel blocker-based combination therapies in patients with hypertension: A randomized controlled trial. *Journal of Hypertension* 2011; **29**: 1649–59.

23 Marre M, Lievre M, Chatellier G, Mann JFE, Passa P, Ménard J. Effects of low dose ramipril on cardiovascular and renal outcomes in patients with type 2 diabetes and raised excretion of urinary albumin: Randomised, double blind, placebo controlled trial (the DIABHYCAR study). *BMJ* 2004; **328**: 495–9.

24 Koudstaal PJ, Algra A, Pop GA, Kappelle LJ, van Latum JC, van Gijn J. Risk of cardiac events in atypical transient ischaemic attack or minor stroke. The Dutch TIA Study Group. *Lancet* 1992; **340**: 630–3.

25 Suzuki H, Kanno Y, Kanai A, *et al.* Effects of candesartan on cardiovascular outcomes in Japanese hypertensive patients. *Hypertension Research* 2005; **28**: 307–14.

26 Zanchetti A, Bond MG, Hennig M, *et al.* Calcium antagonist lacidipine slows down progression of asymptomatic carotid atherosclerosis: Principal results of the European Lacidipine Study on Atherosclerosis (ELSA), a randomized, double-blind, long-term trial. *Circulation* 2002; **106**: 2422–7.

27 Fox KM, Bertrand M, Ferrari R, *et al.* Efficacy of perindopril in reduction of cardiovascular events among patients with stable coronary artery disease: Randomised, double-blind, placebo-controlled, multicentre trial (the EUROPA study). *Lancet* 2003; **362**: 782–8.

28 Amery A, Brixko P, Clement D, *et al.* Mortality and morbidity results from the European Working Party on High Blood Pressure in the Elderly trial. *Lancet* 1985; **325**: 1349–54.

29 Kasanuki H, Hagiwara N, Hosoda S, *et al.* Angiotensin II receptor blocker-based vs. non-angiotensin II receptor blocker-based therapy in patients with angiographically documented coronary artery disease and hypertension: The Heart Institute of Japan Candesartan Randomized Trial for Evaluation in. *European Heart Journal* 2009; **30**: 1203–12.

30 Asayama K, Ohkubo T, Metoki H, *et al.* Cardiovascular outcomes in the first trial of antihypertensive therapy guided by self-measured home blood pressure. *Hypertension Research* 2012; **35**: 1102–10.

31 Sharma AM, Pischon T, Engeli S. Effect of ramipril on cardiovascular events in high-risk patients. *New England Journal of Medicine* 2000; **343**.

32 Beckett NS, Peters R, Fletcher AE, *et al.* Treatment of hypertension in patients 80 years of age or older. *New England Journal of Medicine* 2008; **358**: 1887–98.

33 Lewis EJ, Hunsicker LG, Clarke WR, *et al.* Renoprotective effect of the angiotensin-receptor antagonist irbesartan in patients with nephropathy due to type 2 diabetes. *New England Journal of Medicine* 2001; **345**: 851–60.

34 Brown MJ, Palmer CR, Castaigne A, *et al.* Morbidity and mortality in patients randomised to double-blind treatment with a long-acting calcium-channel blocker or diuretic in the International Nifedipine GITS study: Intervention as a Goal in Hypertension Treatment (INSIGHT). *Lancet* 2000; **356**: 366–72.

35 Pepine CJ, Handberg EM, Cooper-DeHoff RM, *et al.* A Calcium Antagonist vs a Non-Calcium Antagonist Hypertension Treatment Strategy for Patients with Coronary Artery Disease the International Verapamil-Trandolapril Study (INVEST): A Randomized Controlled Trial. *Journal of the American Medical Association* 2003; **290**: 2805–16.

36 Yui Y, Sumiyoshi T, Kodama K, *et al.* Comparison of nifedipine retard with angiotensin converting enzyme inhibitors in Japanese hypertensive patients with coronary artery disease: The Japan Multicenter Investigation for Cardiovascular Diseases-B (JMIC-B) randomized trial. *Hypertension Research* 2004; **27**: 181–91.

37 Lindholm LH, Ibsen H, Dahlöf B, *et al.* Cardiovascular morbidity and mortality in patients with diabetes in the Losartan Intervention For Endpoint reduction in hypertension study (LIFE): A randomised trial against atenolol. *Lancet* 2002; **359**: 1004–10.

38 Schrader J, Lüders S, Kulschewski A, *et al.* Morbidity and mortality after stroke, eprosartan compared with nitrendipine for secondary prevention: Principal results of a prospective randomized controlled study (MOSES). *Stroke* 2005; **36**: 1218–24.

39 Randomized double-blind comparison of a calcium antagonist and a diuretic in elderly hypertensives. National Intervention Cooperative Study in Elderly Hypertensives Study Group. *Hypertension* 1999; **34**: 1129–33.

40 Hansson L, Hedner T, Lund-Johansen P, *et al.* Randomised trial of effects of calcium antagonists compared with diuretics and β-blockers on cardiovascular morbidity and mortality in hypertension: The Nordic Diltiazem (NORDIL) study. *Lancet* 2000; **356**: 359–65.

41 Yusuf S, Teo KK, Pogue J, *et al.* Telmisartan, ramipril, or both in patients at high risk for vascular events. *New England Journal of Medicine* 2008; **358**: 1547–59.

42 MacMahon S, Sharpe N, Gamble G, *et al.* Randomized, placebo-controlled trial of the angiotensin-converting enzyme inhibitor, ramipril, in patients with coronary or other occlusive arterial disease. *Journal of the American College of Cardiology* 2000; **36**: 438–43.

43 Braunwald E, Domanski MJ, Fowler SE, *et al.* Angiotensin-converting-enzyme inhibition in stable coronary artery disease. *New England Journal of Medicine* 2004; **351**. DOI:10.1056/NEJMoa042739.

44 Asselbergs FW, Diercks GFH, Hillege HL, *et al.* Effects of fosinopril and pravastatin on cardiovascular events in subjects with microalbuminuria. *Circulation* 2004; **110**: 2809–16.

45 Eleuteri E. Effect of amlodipine on the progression of atherosclerosis and the occurrence of clinical events. *Italian heart journal Supplement* 2001; **2**: 85–6.

46 Sacco RL, Diener HC, Yusuf S, *et al.* Aspirin and extended-release dipyridamole versus clopidogrel for recurrent stroke. *New England Journal of Medicine* 2008; **359**: 1238–51.

47 Randomised trial of a perindopril-based blood-pressure-lowering regimen among 6,105 individuals with previous stroke or transient ischaemic attack. *Lancet* 2001; **358**: 1033–41.

48 Ogihara T, Nakao K, Fukui T, *et al.* Effects of candesartan compared with amlodipine in hypertensive patients with high cardiovascular risks: Candesartan antihypertensive survival evaluation in Japan trial. *Hypertension* 2008; **51**: 393–8.

49 A Randomized Trial of Intensive versus Standard Blood-Pressure Control. *New England Journal of Medicine* 2015; **373**: 2103–16.

50 Hansson L, Lindholm LH, Ekbom T, *et al.* Randomised trial of old and new antihypertensive drugs in elderly patients: Cardiovascular mortality and morbidity the Swedish trial in old patients with hypertension-2 study. *Lancet* 1999; **354**: 1751–6.

51 Staessen J, Fagard R, Thijs L, *et al.* Randomised double-blind comparison of placebo and active treatment for older patients with isolated systolic hypertension. The Systolic Hypertension in Europe (Syst-Eur) Trial Investigators. Lancet. 1997; **350**: 757–64.

52 Telmisartan T, Assessment R. Effects of the angiotensin-receptor blocker telmisartan on cardiovascular events in high-risk patients intolerant to angiotensin-converting enzyme inhibitors: a randomised controlled trial. *Lancet* 2008; **372**: 1174–83.

53 Tight blood pressure control and risk of macrovascular and microvascular complications in type 2 diabetes: UKPDS 38. UK Prospective Diabetes Study Group. *BMJ* 1998; **317**: 703–13.

54 Ogihara T, Saruta T, Rakugi H, *et al.* Target blood pressure for treatment of isolated systolic hypertension in the elderly: Valsartan in elderly isolated systolic hypertension study. *Hypertension* 2010; **56**: 196–202.

55 Julius S, Kjeldsen SE, Weber M, *et al.* Outcomes in hypertensive patients at high cardiovascular risk treated with regimens based on valsartan or amlodipine: The VALUE randomised trial. *Lancet* 2004; **363**: 2022–31.

56 Zanchetti A, Agabiti Rosei E, Dal Palù C, Leonetti G, Magnani B, Pessina A. The Verapamil in Hypertension and Atherosclerosis Study (VHAS): Results of long-term randomized treatment with either verapamil or chlorthalidone on carotid intima-media thickness. *Journal of Hypertension* 1998; **16**: 1667–76.
